# A Mathematical Analysis of IPT-DMFT

**DOI:** 10.1007/s00220-026-05647-9

**Published:** 2026-06-06

**Authors:** Eric Cancès, Alfred Kirsch, Solal Perrin-Roussel

**Affiliations:** 1 CERMICS, Ecole des Ponts, 6-8 Avenue Blaise Pascal, 77455 Marne-la-Vallée, France; 2 MATHERIALS, Inria Paris, Paris, France

## Abstract

We provide a mathematical analysis of the Dynamical Mean-Field Theory (DMFT), a celebrated representative of a class of approximations in quantum mechanics known as *embedding methods*. Within the simplest setting of this approximation, which we introduce briefly at this point, we prove that, under certain assumptions, the DMFT equations admit a solution for any set of physical parameters. Moreover, we establish some properties of the solution(s). For the reader’s convenience, we provide in appendix a pedagogical and self-contained mathematical formulation of the DMFT equations for the finite Hubbard model. After recalling the definition and properties of one-body time-ordered Green’s functions and self-energies, and the mathematical structure of the Hubbard and Anderson impurity models, we describe the specific *impurity solver* we have studied in this article, namely the Iterated Perturbation Theory (IPT) solver, which can be conveniently formulated using Matsubara’s Green’s functions.

## Introduction

The Dynamical Mean-Field Theory (DMFT) is an approximation method for the quantum many-body problem, which can be regarded as a quantum analog of the mean-field solution of Ising models. It was introduced by Georges and Kotliar in 1992 and first applied to the case of the Hubbard model [26, 27, 42, 53, 62] (throughout this paper, we will only consider the fermionic version of the Hubbard model, also known as Fermi-Hubbard model). It has since been extended to other settings [70] and coupled with Density Functional Theory (DFT) within the so-called DFT+DMFT method [34]. The latter is one of the reference methods for first-principle computations of electronic structures of strongly correlated materials. DMFT belongs to the class of quantum embedding methods, and has since been joined by many other methods such as Density-Matrix Embedding Theory (DMET) [40], Rotationally-Invariant Slave Boson (RISB) method [44], Energy-weighted DMET [22], Quantum Embedding Theory [51], and related methods.

At the time of writing, the mathematical analysis of quantum embedding methods is very limited. The rigorous results we are aware of are those on DMFT contained in Lindsey’s PhD thesis [50], the ones on DMET recently obtained by the first two authors and their collaborators [15], and a few others with a numerically oriented approach such as [20, 76].

The purpose of this article is to establish a rigorous mathematical formulation of the DMFT equations and to prove, in particular, the existence of a solution to the DMFT equations for the Hubbard model within the Iterated Perturbation Theory (IPT) approximation [27]. This relies on the extension of some results from [50, Part VII] and is based upon a reformulation of the DMFT equations as a fixed-point problem in the space of probability measures on the real line.

In the language of linear algebra, solving the fermionic quantum many-body problem consists in computing some spectral properties of a Hermitian matrix $\widehat{H}\in \mathbb {C}^{M \times M}$, the Hamiltonian of the system, such as its ground-state energy (i.e. its lowest eigenvalue), or the partition function

$$\begin{aligned} Z_{\beta ,\epsilon _F}:=\exp \left( -\beta (\widehat{H}- \epsilon _F\widehat{N}) \right) , \end{aligned}$$

where $\beta =\frac{1}{k_\textrm{B}T}$ is the inverse temperature, $\epsilon _F\in \mathbb {R}$ is the chemical potential, and $\widehat{N}$ is the number operator, as well as derivatives of $Z_{\beta ,\epsilon _F}$ with respect to $\beta$, $\epsilon _F$ or parameters of $\widehat{H}$.

The difficulty is that the size *M* of the matrix $\widehat{H}$ can be huge (up to $10^{30}$ or more in some applications). Fortunately, the Hamiltonian $\widehat{H}$ has specific properties, allowing one to use taylored methods. Indeed, in most applications, $\widehat{H}$ is the matrix of a Hamiltonian operator acting on a fermionic Fock space $\mathcal {F}$, containing only one- and two-body terms, and satisfying symmetry properties (particle number conservation, spin, and possibly space, isospin, or time-reversal symmetries). Identifying the one-body state space $\mathcal {H}_{ }$ with $\mathbb {C}^{2L}$, it holds $M=2^{2L}$ and

$$\begin{aligned} \mathcal {F}= \bigoplus _{N=0}^L \mathcal {H}_{{N}}, \end{aligned}$$

where the *N*-particle sector $\mathcal {H}_{{N}}=\bigwedge ^N \mathcal {H}_{ }$ of the Fock space is of dimension $$\begin{pmatrix} 2L \\ N \end{pmatrix}$$. In this decomposition, $\widehat{N}$ is a block diagonal operator, the block corresponding to $\mathcal {H}_{{N}}$ being equal to *N* times the identity matrix. If $\widehat{H}$ is particle-number conserving, then it is also block-diagonal in this decomposition (equivalently $\widehat{H}$ and $\widehat{N}$ commute). Since it only contains one- and two-body terms, then $\widehat{H}$ has a compact representation in the second quantization formalism involving a Hermitian matrix $H^0 \in \mathbb {C}^{2L \times 2L}$ and a fourth-order tensor $V \in \mathbb {C}^{2L \times 2L \times 2L \times 2L}$. Spin, space, isospin, or time-reversal symmetries allow one to further reduce the complexity of the representation of $\widehat{H}$ and refine its block diagonal structure. Still, solving the quantum many-body problem remains extremely challenging.

Quantum embedding methods can be seen as domain decomposition methods in the Fock space, using a partition of the *L* “sites” (also called orbitals) of the model into *P* non-overlapping clusters $(\Lambda _p)_{1 \le p \le P}$ of cardinalities $L_p:=|\Lambda _p|$. Without loss of generality, we can assume that the first cluster consists of the first $L_1$ orbitals, the second cluster of the next $L_2$ orbitals, and so on. Each cluster is modeled by an impurity consisting in $L_p$ sites. The impurity is coupled with virtual sites called bath orbitals, resulting in a quantum many-body problem known as an impurity model. In DMET, the number of bath orbitals is exactly equal to $L_p$ so that the impurity quantum many-body problem is of size $M_p:=2^{4L_p}$. In practice, DMET impurity problems are solved either by brute-force diagonalization (full CI) if $L_p$ is not too large, or by low-rank tensor methods (e.g. Density Matrix Renormalization Group, DMRG [74]). In DMFT, the impurity problem can be much larger, but the impurity Hamiltonian has the relatively simple form of an Anderson Impurity Model (AIM): within each of the *P* impurity models, bath orbitals do not contribute to two-body interactions, and are coupled with impurity orbitals only via one-body interactions. It can be shown that the AIM associated with the *p*-th cluster can be completely described by the restriction of $\widehat{H}$ to the *p*-th impurity’s orbitals and a hybridization function $\Delta _p: \mathbb {C}{\setminus } \mathbb {R}\rightarrow \mathbb {C}^{L_p \times L_p}$. AIMs are usually solved in practice either by a quantum Monte Carlo method [63], or by an approximate solver such as the IPT (Iterative Perturbation Theory) solver [27] considered in this article. The IPT solver was introduced in the seminal paper [79], and is still used to study very challenging systems such as moiré heterobilayers [71].

Quantum embedding methods are self-consistent theories: the *P* impurity problems are coupled through a mean-field defined on the whole quantum system with *L* orbitals.

In DMET, the role of the mean-field is played by an approximation $D \in \mathbb {C}^{2L \times 2L}$ of the ground-state one-body density matrix (1-RDM) of the system. The matrix *D* allows one to define an impurity problem for each cluster, and the self-consistent condition is that for each cluster *p*, the diagonal block of *D* corresponding to this cluster agrees with the restriction of the exact ground-state 1-RDM of the *p*-th impurity problem to the cluster *p*. It is expected that at self-consistency the diagonal blocks of *D* corresponding to the cluster decomposition are good approximations of the diagonal blocks of the exact ground-state 1-RDM of the whole system [15].

In DMFT, the role of the mean-field is played by an approximation *G* of the exact one-body Green’s function [52] associated with some steady state, usually the grand-canonical thermodynamical equilibrium state. One-body Green’s functions can be represented by analytic functions $G: \mathbb {C}\setminus \mathbb {R}\rightarrow \mathbb {C}^{2L \times 2L}$ and are thus computationally tractable objets for values of *L* up to a few thousands. The function *G* is a particular holomorphic extension of the Fourier transform of the time-ordered Green’s function. Loosely speaking, the latter is a stationary time-correlation function obtained by creating (resp. annihilating) a particle at time $t_0=0$ (resp. at $t<0$), letting the system evolve from $t_0$ to *t* (resp. from *t* to $t_0$), and annihilating the extra particle (resp. restoring the missing particle) at time *t* (resp. at time $t_0$). The exact one-body Green’s function contains a lot of valuable information about the quantum system under investigation. In particular, the 1-RDM of the steady state, hence the expectation value of any one-body observable, can be easily extracted from it. The same holds true for the average energy, thanks to Galitski-Migdal’s formula [31, 52]. Also, the poles of the analytic continuation of *G* to the real-axis correspond to the one-particle excitation energies measured in photoemission and inverse-photoemission spectroscopies [80]. Remarkably, the exact Green’s function $G^0$ of a non-interacting system, i.e. of a many-body Hamiltonian $d\Gamma ({H^0})$ which is the second quantization of a one-body Hamiltonian is simply the resolvent of $H^0$: $G^0(z)= (z-H^0)^{-1}$, whatever the reference steady state. The self-energy of an interacting system with Hamiltonian $\widehat{H}= d\Gamma ({H^0}) + \widehat{H}^I$, where $\widehat{H}^I$ accounts for the (two-body) interactions, is the function $\Sigma : \mathbb {C}\setminus \mathbb {R}\rightarrow \mathbb {C}^{2L \times 2L}$ defined by

$$\begin{aligned} \Sigma (z) = G^0(z)^{-1} - G(z)^{-1} \quad \text{ or } \text{ equivalently } \quad G(z) = (z-H^0-\Sigma (z))^{-1}. \end{aligned}$$

DMFT consists in

- Approximating the exact self-energy of the whole system by a block diagonal self-energy $\Sigma = \text{ block-diag }(\Sigma _1, \dots , \Sigma _P)$, with $\Sigma _p: \mathbb {C}\setminus \mathbb {R}\rightarrow \mathbb {C}^{2L_p \times 2L_p}$ compatible with the cluster decomposition. This condition is sometimes called the DMFT approximation;
- Imposing the self-consistent conditions that for each cluster
  - The self-energy $\Sigma _p$ agrees with the restriction to the impurity of the exact self-energy of the associated AIM,
  - The restriction to the cluster of the approximate Green’s function of the whole system agrees with the restriction to the impurity of the exact Green’s function of the AIM. This condition is often referred to as the self-consistent condition.

In practice, the DMFT equations are solved by fixed-point iterations. The input of iteration *n* is a collection of *P* hybridization functions $(\Delta _p^{(n)})_{1 \le p \le P}$. At step 1, the *P* AIM problems with hybridization functions $\Delta _p^{(n)}$ are solved in parallel, in order to compute *P* cluster self-energies $\Sigma _p^{(n)}$, yielding an approximation $\Sigma ^{(n)} = \text{ block-diag }(\Sigma _1^{(n)}, \cdots \Sigma _P^{(n)})$ of the self-energy of the whole system. At step 2, the above two self-consistent conditions are combined yielding a new set $(\Delta _p^{(n+1)})_{1 \le p \le P}$ of hybridization functions. The DMFT iteration scheme can therefore be sketched as

1 $$\begin{aligned} \Delta ^{(n)}:=(\Delta _p^{(n)})_{1 \le p \le P} \mathop {\longrightarrow } \Sigma ^{(n)}:=(\Sigma _p^{(n)})_{1 \le p \le P} \mathop {\longrightarrow } \Delta ^{(n+1)}:=(\Delta _p^{(n+1)})_{1 \le p \le P}, \end{aligned}$$

or written in the more compact form

2 $$\begin{aligned} \Delta ^{(n+1)} = f^\textrm{DMFT}\left( \Delta ^{(n)}\right) . \end{aligned}$$

Of course, this basic self-consistent loop can be stabilized and accelerated using e.g. damping and Anderson-Pulay extrapolation methods. In this article, we forego an in-depth analysis of the iterative scheme and its convergence, opting instead to direct our attention towards a fundamental inquiry: the existence of solutions of the DMFT equations. Specifically, we address the question of the existence of a fixed-point of the DMFT map $f^\textrm{DMFT}$, a critical aspect which, to our knowledge remains unestablished in the current literature.

This article is organized as follows. In Sect. 3, we briefly introduce the DMFT approximation of a Hubbard model, emphasizing the mathematical framework rather than the approximation’s derivation (see Appendix A for the latter). Of particular importance is the fact that the key mathematical objects involved in DMFT (exact and approximate one-body Green’s functions and self-energies, hybridization functions) are all negatives of Pick functions. Recall that scalar Pick functions are analytic functions from the open upper half-plane to the closed upper-half plane [56, 60]. An interesting property of scalar Pick functions, which we use extensively in our analysis, is that any Pick function admits an integral representation involving a positive Borel measure on $\mathbb {R}$, called its Nevanlinna-Riesz measure [56]. Analogous properties hold true for matrix-valued Pick functions [30]. For the Hubbard model with a finite number of sites, the exact Green’s function and self-energy can be extended to meromorphic functions on $\mathbb {C}$ with finite numbers of poles (which appear to lie on the real-line), and are therefore represented by finite and finitely supported Nevanlinna-Riesz measures.

We then focus on the single-site, translation-invariant, paramagnetic IPT-DMFT approximation of the Hubbard model, for which $P=L$ and $L_1=\cdots = L_P=1$. We show that these equations have no solutions in the class of (negatives of) Pick functions with finite and finitely supported Nevanlinna-Riesz measures, but do have solutions in the set of (negatives of) Pick functions. More precisely, equation (2) has a translation invariant fixed point $(\Delta ,\dots ,\Delta )$, $\Delta$ being the negative of a Pick function whose Nevanlinna-Riesz measure has the form $c \nu$, where $c \in \mathbb {R}_+$ is a fixed constant only depending on the matrix $H^0$, and $\nu$ a Borel probability measure on $\mathbb {R}$. To obtain the latter result, we show that the IPT-DMFT iteration map $f^\textrm{DMFT}$ in (2) can be rewritten as a map $F^{\textrm{DMFT}}: \mathcal {P}(\mathbb {R})\rightarrow \mathcal {P}(\mathbb {R})$, from the set $\mathcal {P}(\mathbb {R})$ of Borel probability measures on $\mathbb {R}$ to itself, which is continuous for the weak topology. We conclude by checking that the Schauder-Singbal’s fixed-point theorem [68] can be applied to this setting.

In Appendix A, we provide a mathematical introduction to DMFT for the Hubbard model aimed at being accessible to readers unfamiliar with this theory. The Hubbard model provides insights into the behavior of electrons in strongly correlated systems. Its integration within the DMFT framework offers a powerful tool for understanding the interplay between electrons, shedding light on phenomena such as metal-insulator transitions. We recall the basics of second quantization formalism, the formulation of the Hubbard and Anderson impurity models, the definitions of one-body Green’s functions, self-energies, and hybridization functions, and the precise formulation of the DMFT equations. We then present Matsubara’s formalism, and motivate the introduction of the paramagnetic IPT solver. Finally, we highlight how, with such a solver, spin degeneracy can be lifted to reduce the single-site, translation-invariant, paramagnetic IPT-DMFT equations to a set of equations dealt with in the main body of this article.

Finally, the IPT solver being formulated in terms of an Analytic Continuation Problem (ACP), Appendix B is devoted to an introduction to these problems. In particular, we introduce a uniqueness result which proved to be very useful for the analysis of the IPT-DMFT equations.

## IPT-DMFT Framework

Let us introduce and recall some useful notation. We denote by

$$\begin{aligned} \mathbb {C}_+:= \{z\in \mathbb {C}\ | \ \Im (z)>0\} \end{aligned}$$

the complex open upper half-plane, that is the set of complex numbers with positive imaginary part. For a matrix $M\in \mathbb {C}^{n\times n}$, $n\ge 1$, the imaginary part of the Hermitian matrix *M* is defined by

$$\begin{aligned} \Im (M) = \frac{M-M^\dagger }{2i}. \end{aligned}$$

The set of Hermitian matrices of size *n* is denoted by $\mathcal {S}_n(\mathbb {C})$, and the set of positive-semidefinite matrices by $\mathcal {S}_n^+(\mathbb {C})$. The notation $M \ge 0$ (resp. $M > 0$) means that the matrix *M* is positive semidefinite (resp. positive definite). In the following, we will also deal with measure and probability theory. The set of finite signed Borel measures on $\mathbb {R}$ is denoted by $\mathcal {M}(\mathbb {R})$. The subset $\mathcal {M}_+(\mathbb {R})\subset \mathcal {M}(\mathbb {R})$ is the set of finite positive Borel measures on $\mathbb {R}$, and we recall that the subset $\mathcal {P}(\mathbb {R})\subset \mathcal {M}_+(\mathbb {R})$ denotes the set of Borel probability measures on $\mathbb {R}$.

For a positive Borel measure $\mu$ on $\mathbb {R}$, we say that $\mu$ has a finite moment of order $k\in \mathbb {N}$ if $\int _\mathbb {R}|\varepsilon |^k d\mu (\varepsilon )<\infty$. In this case, we denote by $m_k(\mu )=\int _\mathbb {R}\varepsilon ^k d\mu (\varepsilon )$ its *k*-th moment. In particular, $\mu$ is finite if and only if it has a finite moment of order 0. In this case, $\mu \in \mathcal {M}_+(\mathbb {R})$ and its 0-th moment is called the *mass* of $\mu$, denoted by $\mu (\mathbb {R})=m_0(\mu )=\int _\mathbb {R}d\mu$. These notation and considerations extend to the case of matrix-valued measures, as will be discussed in the next section.

A Hubbard model is built on an underlying connected, undirected, loopless graph (loopless means there is no edge joining a vertex to itself). Apart from the results stated in Sect. 3.4, we restrict ourselves to finite graphs, that is to Hubbard models with finite numbers of sites. Note that Hubbard graphs do not need to be considered as embedded in some *d*-dimensional physical space. We will however make use of the expression “translation-invariant setting” to refer to cases when the underlying graph is vertex-transitive. Recall that a graph $\mathcal {G}_{{H}}=(\Lambda ,E)$ is called *vertex-transitive* if for all $\lambda _1,\lambda _2 \in \Lambda$, there exists a graph automorphism $\tau :\Lambda \rightarrow \Lambda$ such that $\tau (\lambda _1)=\lambda _2$ (a graph automorphism on $\mathcal {G}_{{H}}$ is a bijection $\tau :\Lambda \rightarrow \Lambda$ such that $\{i,j\} \in E$ if and only if $\{\tau (i),\tau (j)\} \in E$). In the particularly important case of a *d*-dimensional periodic lattice (for instance, a cubic or hypercubic lattice), the vertex-transitivity of the graph is precisely a consequence of the lattice’s translation invariance. We hope that this slight abuse of terminology will not cause any confusion.

### One-Body Green’s Function and Self-Energy as Pick Functions

Our mathematical framework intersects with the realm of complex analysis pioneered by Pick [60] and Nevanlinna [56], focusing on the study of so-called Pick functions. A *Pick function* is a map $f: \mathbb {C}_+\rightarrow \overline{\mathbb {C}_+}$ which is analytic. In this article, we use the term Pick functions, but several terminologies coexist in the literature: Nevanlinna functions, Herglotz functions, Riesz functions, or *R*-functions. A *Pick matrix* is an analytic map $f:\mathbb {C}_+\rightarrow \mathbb {C}^{n\times n}$, $n\ge 1$, such that for all $z\in \mathbb {C}_+, \Im (f(z))\ge 0$ (i.e. $\Im (f(z))\in \mathcal {S}_n^+(\mathbb {C})$). Sometimes, it is convenient to extend Pick matrices to the lower-half-plane $\mathbb {C}_-$. In this case, the usual convention is to set for all $z \in \mathbb {C}_-, f(z)=f(\overline{z})^\dagger$ [30].

As mentioned in the introduction, DMFT is an approximation method of the quantum one-body Green’s function *G* (which we refer to as Green’s function in short). In this section, we recall the main mathematical properties of this object, and refer to Appendix A for a short, pedagogical introduction to Green’s functions.

Consider a many-body quantum system with finite-dimensional one-body state space $\mathcal {H}_{ }$ and Hamiltonian $\widehat{H}\in \mathcal {S}(\mathcal {F})$ on the Fock space $\mathcal {F}$ associated with $\mathcal {H}_{ }$. Assume that the system is in an equilibrium state characterized by the density operator $\widehat{\rho }\in \mathcal {S}(\mathcal {F}(\mathcal {H}_{ }))$. Recall that an equilibrium state is a stationary solution to the quantum Liouville equation, and thus a state commuting with the Hamiltonian ($[\widehat{H},\widehat{\rho }]=0$). The Green’s function $G:\mathbb {C}_+\rightarrow \mathcal {L}(\mathcal {H}_{ })$ is an operator-valued function defined on the upper half-plane and depending on both $\widehat{H}$ and $\widehat{\rho }$. A crucial mathematical property of *G* used in our analysis is the following (see Proposition A.1 in Appendix A):
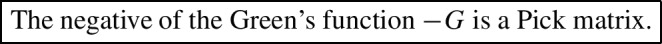


For a given pair of operators $(\widehat{H},\widehat{\rho })$, finding the associated Green’s function is very challenging. Nevertheless, when the Hamiltonian is the second quantization $\widehat{H}=d\Gamma ({H^0})$ of a one-body Hamiltonian $H^0\in \mathcal {S}(\mathcal {H}_{ })$, the Green’s function, denoted by $G^0$ in this special case, is the resolvent of the one-body Hamiltonian, regardless of the density operator $\widehat{\rho }$: for all $z \in \mathbb {C}_+$,

$$\begin{aligned} G^0(z)=\left( z-H^0\right) ^{-1}. \end{aligned}$$

For a Hamiltonian of the form $\widehat{H}=d\Gamma ({H^0})+\widehat{H}^I$ (these are the only Hamiltonians considered in this article), the self-energy $\Sigma :\mathbb {C}_+\rightarrow \mathcal {L}(\mathcal {H}_{ })$ quantifies the discrepancy between the interacting Green’s function *G* associated with the pair $(\widehat{H},\widehat{\rho })$ and the non-interacting Green’s function $G^0$ associated with $d\Gamma ({H^0})$: it is defined, for all $z \in \mathbb {C}_+$, by

$$\begin{aligned} \Sigma (z)=G^0(z)^{-1}-G(z)^{-1}=z-H^0-G(z)^{-1}. \end{aligned}$$

The second crucial property used in our analysis is the following (see again Proposition A.1): 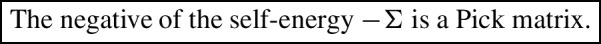


### IPT-DMFT Equations

In this article, we are interested in proving the existence of solution(s) to the DMFT equations, formulated within the approximation of the Iterated Perturbation Theory impurity (IPT) solver. A detailed introduction to IPT can be found in Appendix A. In the present section, we only briefly motivate this approach and introduce the corresponding equations.

The purpose of DMFT is to provide an approximation of the Green’s function of a Hubbard model in a generic Gibbs state. Let us recall that a Hubbard model considers particles living on a connected, undirected, loopless graph $\mathcal {G}_{{H}}=(\Lambda ,E)$, where each vertex can host up to two fermions. The Hubbard Hamiltonian $\widehat{H}_H$ models the fact that fermions can jump from one site to another — the amplitude of this effect being entirely defined by the hopping matrix $T_{ }:E\rightarrow \mathbb {R}$ — and that fermions interact with one another strictly locally, when they sit on the same site — the amplitude of this interaction being described by the on-site interaction $U_{ }:\Lambda \rightarrow \mathbb {R}$ (see Definition A.7 for a precise formulation).

The DMFT approximation is defined through a partition $\mathfrak {P}$ of the Hubbard vertices $\Lambda$: to each of the induced subgraphs $\left( \Lambda _p,E_p\right)$ is associated an Anderson Impurity model. The latter describes fermions that live in two subsystems: the impurity on the one-hand, which is prescribed by the induced subgraph in the present case and whose one-particle space is denoted by $\mathcal {H}_{{\textrm{imp}}}$, and the bath on the other hand, with one-partice space $\mathcal {H}_{{\textrm{bath}}}$ which is an unknown of the DMFT approximation. The Anderson Impurity Model (AIM) Hamiltonian ${\widehat{H}}_\textrm{AIM}$ models the fact that fermions can jump between the bath levels and the impurity vertices — the magnitude of this process being described by the coupling $V_{ }:\mathcal {H}_{{\textrm{imp}}} \rightarrow \mathcal {H}_{{\textrm{bath}}}$ — between impurity vertices — the magnitude of this process being described by the restriction of the hopping matrix $T_{ }$ to $E_p$ — and that fermions interact with one another only when they sit on the same impurity site — the magnitude of this interaction being described by the restriction of the on-site interaction $U_{ }$ to $\Lambda _p$ (see Appendix A.3 for a precise formulation).

In an Anderson Impurity model, interactions between fermions are localized within the impurity subsystem. As a consequence, the self-energy of an AIM satisfies a sparsity pattern (see Theorem A.10): using the decomposition $\mathcal {H}_{ }=\mathcal {H}_{{\textrm{imp}}} \oplus \mathcal {H}_{{\textrm{bath}}}$, we have

3 $$\begin{aligned} \Sigma (z)=\left( \begin{array}{ll} \Sigma _{\textrm{imp}}(z) &  0 \\ 0 & 0 \end{array} \right) . \end{aligned}$$

Moreover, the impurity self-energy $\Sigma _{\textrm{imp}}$ only depends on the impurity parameters (the restriction of $T_{ }$ and $U_{ }$ to $(\Lambda _p,E_p$)) and on the so-called hybridization function $\Delta :\mathbb {C}_+\rightarrow \mathcal {L}(\mathcal {H}_{{\textrm{imp}}})$, defined, for all $z \in \mathbb {C}_+$, by

$$\begin{aligned} \Delta (z)=V\left( z-H^0_{\textrm{bath}}\right) ^{-1}V^\dagger , \end{aligned}$$

where $H^0_{\textrm{bath}} \in \mathcal {S}(\mathcal {H}_{{\textrm{bath}}})$ is the restriction of the AIM one-body Hamiltonian to the bath subspace (see Definition A.9 for a precise formulation). Note that
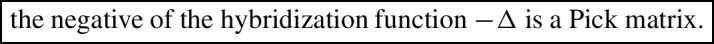


For fixed impurity parameters, the map that associates the hybridization function $\Delta$ to the corresponding impurity self-energy $\Sigma _{\textrm{imp}}$ is called an impurity solver. In this work, we considered an approximate impurity solver, the Iterated Perturbation Theory (IPT), in the single-site, translation-invariant, paramagnetic setting: as explained in Appendix A.6, this setting allows one to consider that $\Delta$ and $\Sigma _{\textrm{imp}}$ are scalar-valued. In other words,
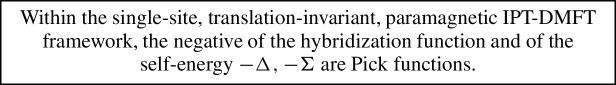


The IPT solver provides the self-energy as the solution to an Analytic Continuation Problem (ACP) (see Appendix B for an introduction to the Nevanlinna-Pick analytic continuation problem): given a hybridization function $-\Delta : \mathbb {C}_+\rightarrow \overline{\mathbb {C}_+}$, the impurity self-energy $\Sigma _{\textrm{imp}}$ is a solution to

4 $$\begin{aligned} -\Sigma _\textrm{imp}: \mathbb {C}_+\rightarrow \overline{\mathbb {C}_+}, \quad \Sigma _\textrm{imp} \text { is analytic}, \quad \forall n\in \mathbb {N}, \ \Sigma _\textrm{imp}(i\omega _n) = \Sigma _{\textrm{imp},n}, \end{aligned}$$

with, for all $n \in \mathbb {N}$,

5 $$\begin{aligned} \Sigma _{\textrm{imp},n}=U_{ }^2\int _{0}^\beta e^{i\omega _n \tau } \left( \frac{1}{\beta }\sum _{n' \in \mathbb {Z}}\left( i\omega _{n'} - \Delta (i\omega _{n'}) \right) ^{-1} e^{-i\omega _{n'} \tau } \right) ^3 d \tau , \end{aligned}$$

where $\omega _n=(2n+1)\pi /\beta$ is the *n*-th Matsubara’s frequency at given inverse temperature $\beta \in \mathbb {R}_+^*$ (see Appendix A.5.1 for an introduction to Matsubara’s formalism).

This solver, if well-defined, provides one relation between the hybridization function and the impurity self-energy. The second relation between these quantities is provided by the DMFT approximation: as explained in details in Appendix A.4, in the single-site, translation-invariant, paramagnetic setting (in which the hopping matrix is constant $T_{ } \in \mathbb {R}$), this equation reads, for all $z \in \mathbb {C}_+$,

6 $$\begin{aligned} \Delta (z)=W\left( z-H^0_\perp -\Sigma (z)\right) ^{-1}W^\dagger , \end{aligned}$$

where $W \in \mathbb {R}^{L-1}$ and $H^0_\perp \in \mathcal {S}(\mathbb {R}^{L-1})$ are defined by the following block decomposition of the adjacency matrix $\textrm{Adj}_{\mathcal {G}_{{H}}}$ of the considered Hubbard graph $\mathcal {G}_{{H}}$:

7 $$\begin{aligned} T\textrm{Adj}_{\mathcal {G}_{{H}}}=\left( \begin{array}{ll} 0 &  W \\ W^\dagger &  H^0_\perp \end{array}\right) . \end{aligned}$$

Finally, restricting ourselves to single-site, translation-invariant, paramagnetic framework, and denoting by $\Sigma$ the single-site impurity self-energy $\Sigma _\textrm{imp}$, we get from (3)–(7) the set of coupled equations

8a $$\begin{aligned}  &   \Delta (z) = W(z-H^0_\perp -\Sigma (z))W^\dagger , \end{aligned}$$

8b $$\begin{aligned}  &   -\Sigma \text { is a Pick function solution to the interpolation problem}\nonumber \\  &   \forall n\in \mathbb {N}, \Sigma (i\omega _n)=U_{ }^2\int _{0}^\beta e^{i\omega _n \tau } \left( \frac{1}{\beta }\sum _{n' \in \mathbb {Z}}\left( i\omega _{n'} - \Delta (i\omega _{n'}) \right) ^{-1} e^{-i\omega _{n'} \tau } \right) ^3 d \tau . \end{aligned}$$

## Main Results

The main results of this article deal with the existence and properties of solution(s) to the IPT-DMFT equations in the single-site, translation-invariant, paramagnetic setting: we will prove the following.

### Mainresults

For all connected, undirected, loopless and vertex-transitive graph $\mathcal {G}_{{H}}=(\Lambda ,E)$, for all $T_{ },U_{ } \in \mathbb {R}$, $\beta \in \mathbb {R}_+^*$, there exist Pick functions $-\Delta ,-\Sigma$ satisfying the single-site, translation-invariant, paramagnetic IPT-DMFT equations (8a)–(8b). More precisely, such solutions have the following form: there exist a probability measure $\nu \in \mathcal {P}(\mathbb {R})$ and a finite, positive, Borel measure $\mu \in \mathcal {M}_+(\mathbb {R})$ such that, for all $z \in \mathbb {C}_+$,

9 $$\begin{aligned} \Delta (z) = \vert W \vert ^2 \int _\mathbb {R}\frac{1}{z-\varepsilon } d\nu (\varepsilon ), \quad \Sigma (z)=\int _\mathbb {R}\frac{1}{z-\varepsilon } d\mu (\varepsilon ). \end{aligned}$$

Apart from the limit cases $$U_{ }=0$$ or $$ T_{ }=0$$ (for which the solution is unique), any solution of this form is such that $$\nu , \mu $$ are not finitely supported. Finally, any solution of this form is such that $$\nu ,\mu $$ have finite moments up to any order.

To get a deeper insight into this statement, let us remind the reader that Pick functions are tightly related to positive measures, according to the following integral representation theorem, which appears to be particularly useful in our analysis.

### Theorem 3.1

(Nevanlinna-Riesz representation theorem [30]). Let $f: \mathbb {C}_+\rightarrow \mathbb {C}^{n \times n}$ be a Pick matrix. There exist $a\in \mathcal {S}_n^+(\mathbb {C})$, $b\in \mathcal {S}_n(\mathbb {C})$ and $\mu$ a Borel $\mathcal {S}_n^+(\mathbb {C})$-valued measure on $\mathbb {R}$ such that $(1+|\varepsilon |)^{-1}$ is $\mu$-integrable and

10 $$\begin{aligned} \forall z\in \mathbb {C}_+, \quad f(z) = az+b+\int _\mathbb {R}\left( \dfrac{1}{\varepsilon -z}-\dfrac{\varepsilon }{1+\varepsilon ^2}\right) d\mu (\varepsilon ). \end{aligned}$$

The measure $\mu$ is called the Nevanlinna-Riesz measure of *f*, and

$$\begin{aligned} a = \underset{y\rightarrow +\infty }{\lim }\ \dfrac{1}{iy} f(iy), \qquad b = \Re (f(i)):=(f(i)+f(i)^\dagger )/2. \end{aligned}$$

In the particular case of Pick functions, i.e. $n=1$, we have $a\ge 0$, $b\in \mathbb {R}$, and $\mu$ is a positive Borel measure on $\mathbb {R}$, with the same integrability condition.

Our result therefore states that there exists a solution $(\Delta , \Sigma )$, such that $-\Delta$ and $-\Sigma$ are not generic Pick functions but Cauchy-Stieltjes transforms [28] of a probability measure (up to a fixed multiplicative constant) and a finite, positive, Borel measure respectively.

An important remark is that, given a sequence $(\Sigma _{\textrm{imp},n})_{n \in \mathbb {N}}$ computed via (5) from an admissible hybridization function $\Delta$, there is no guarantee that the Analytic Continuation Problem (4) admits a solution nor, if it does, that this solution is unique.

In Sect. 3.1, we study this problem for the functional spaces of hybridization functions/self-energies associated with finite-dimensional Anderson Impurity models. Based on an interpolation result stated in Appendix B, we show in Proposition 3.2 that for such a hybridization function (which is the Cauchy-Stieltjes transform of a *finitely supported* measure $\nu$), the considered ACP admits a *unique* solution $-\Sigma$, which appears to also be the Cauchy-Stieltjes transform of a finitely supported measure $\mu$. We then show in Proposition 3.3 that, apart from the limit cases $U=0$ or $T=0$, there is no solution $(-\Delta ,-\Sigma )$ to (8a)–(8b) which are Cauchy-Stieltjes transforms of finitely-supported measures. We prove this by reformulating (8a)–(8b) as a fixed point problem on the set of finitely-supported probability measures, and by proving that the cardinality of the support of the measure strictly increases when applying the fixed-point map to a finitely supported probability measure.

In Sect. 3.2, we extend the set of admissible $(-\Delta ,-\Sigma )$ to include Cauchy-Stieltjes transforms of measures with arbitrary support. In this setting, we show that the two maps in (1) whose combination leads to the DMFT map $f^\textrm{DMFT}$ are well-defined between appropriate functional spaces. More precisely, we show in Proposition 3.5 that the Bath Update map (8a), reformulated in terms of Nevanlinna-Riesz measures, is well-defined, and in Proposition 3.7 that the IPT solver (solving (8b)), reformulated in terms of Nevanlinna-Riesz measures, admits a unique continuous extension for the weak topology. This strategy allows us to avoid dealing with ACPs for which we were unable to conclude on the uniqueness, and provides, eventually, solutions to the IPT-DMFT equations.

In Sect. 3.3, we state our main result (Theorem 3.8) on the existence of a solution to the single-site, translation-invariant, paramagnetic IPT-DMFT equations, which is a fixed-point of the DMFT map. We also state Proposition 3.9 which shows that such solutions have finite moments at any order.

### Absence of finite dimensional bath solutions

As recalled previously, the mathematical study of the DMFT equations is very limited. The only results the authors are aware of are those contained in M. Lindsey’s PhD Thesis [50, Part VII], and are concerned with the well-posedness of the so-called “DMFT loop". Note that this map is referred to as the “Bath Update map" ($\Sigma \rightarrow \Delta$) in this article.

To introduce the results by Lindsey et al., let us recall that, for finite-dimensional Anderson Impurity Models, the self-energy $\Sigma$ and the hybridization function $\Delta$ have the following integral representation ([50], Definition A.9): there exist $C \in \mathbb {R}$ and $\mu ,\nu$ two finite, finitely supported and positive Borel measures on $\mathbb {R}$ such that, for all $z \in \mathbb {C}_+$,

11 $$\begin{aligned} \Sigma (z)&=C+ \int _\mathbb {R}\frac{1}{z-\varepsilon } d\mu (\varepsilon )=C+\sum _{\varepsilon \in \textrm{supp}(\mu )}\frac{\mu (\{\varepsilon \})}{z-\varepsilon },\end{aligned}$$

12 $$\begin{aligned} \Delta (z)&=\int _{\mathbb {R}}\frac{1}{z-\varepsilon } d\nu (\varepsilon )=\sum _{\varepsilon \in \textrm{supp}(\nu )}\frac{\nu (\{\varepsilon \})}{z-\varepsilon }, \end{aligned}$$

where the sums are finite. The main result by Lindsey et al. [50, Corollary 20, Part VII] states that, given a self-energy $\Sigma$ of the form (11), the hybridization function $\Delta$ defined from $\Sigma$ by Eq. (8a) is of the form (12).

Implementing on their result, we took this specific integral representation as a starting point for our analysis: the first result deals with the Iterated Perturbation Theory impurity solver, which we show below to be well-defined for such hybridization functions and self-energies.

#### Proposition 3.2

(IPT solver for finite-dimensional AIMs). Let $U \in \mathbb {R}$, $\Delta : \mathbb {C}_+\rightarrow \mathbb {C}$ of the form

13 $$\begin{aligned} \forall z \in \mathbb {C}_+, \quad \Delta (z) = \sum _{k=1}^K \frac{a_k}{z-\varepsilon _k}, \quad \text{ with } \quad \varepsilon _1< \varepsilon _2< \cdots < \varepsilon _K \text{ and } a_k > 0 \text{ for } \text{ all } 1 \le k \le K,\nonumber \\ \end{aligned}$$

and $(\Sigma _{n})_{n \in \mathbb {N}}$ defined by

$$\begin{aligned} \forall n \in \mathbb {N}, \quad \Sigma _{n}=U_{ }^2\int _{0}^\beta e^{i\omega _n\tau }\left( \frac{1}{\beta } \sum _{n' \in \mathbb {Z}}\left( i\omega _{n'} - \Delta (i\omega _{n'})\right) ^{-1} e^{-i\omega _{n'}\tau }\right) ^3 d\tau . \end{aligned}$$

Then, the Analytic Continuation Problem (ACP)

14 $$\begin{aligned} -\Sigma : \mathbb {C}_+\rightarrow \overline{\mathbb {C}_+}, \quad \Sigma \text { is analytic}, \quad \forall n\in \mathbb {N}, \Sigma (i\omega _n) = \Sigma _{n} \end{aligned}$$

has a unique solution, which is given by

15 $$\begin{aligned} \forall z \in \mathbb {C}_+, \quad \Sigma ^{{IPT}}(z)= U_{ }^2\sum _{k_1,k_2,k_3=1}^{K+1}\frac{a'_{k_1,k_2,k_3}}{z-\left( \varepsilon _{k_1}'+\varepsilon _{k_2}'+\varepsilon _{k_3}'\right) }, \end{aligned}$$

where $\varepsilon _1'< \varepsilon _2'< \cdots < \varepsilon '_{K+1}$ are the $(K+1)$ real roots of the equation

$$\begin{aligned} \varepsilon -\Delta (\varepsilon )=0, \end{aligned}$$

which satisfy the interlacing relation

$$\begin{aligned} \varepsilon _1'< \varepsilon _1< \varepsilon _2'< \varepsilon _2< \cdots< \varepsilon _{K} < \varepsilon '_{K+1}, \end{aligned}$$

and for all $k_1,k_2,k_3 \in [\![ {1},{K+1}]\!]$, $a_{k_1,k_2,k_3}$ is defined by

16 $$\begin{aligned} a'_{k_1,k_2,k_3}=\frac{1+e^{-\beta (\varepsilon _{k_1}'+\varepsilon _{k_2}'+\varepsilon _{k_3}')}}{\left( 1+e^{-\beta \varepsilon _{k_1}'}\right) \left( 1+e^{-\beta \varepsilon _{k_2}'}\right) \left( 1+e^{-\beta \varepsilon _{k_3}'}\right) } \prod _{i=1}^3\left( 1-\Delta '(\varepsilon _{k_i}')\right) ^{-1} >0. \end{aligned}$$

Combined with the results by Lindsey et al., this proves that the DMFT iteration loop

$$\begin{aligned} \Delta ^{(n)} \xrightarrow {\mathrm{Eqs.} \;~(4)-(5)}\Sigma ^{(n)} \xrightarrow {\mathrm{Eq.} \; ~(8a)} \Delta ^{(n+1)} \end{aligned}$$

is well-defined on the set of Cauchy-Stieltjes transforms of finite, finitely-supported and positive Borel measures. Nevertheless, the following proposition highlights the fact that the single-site, translation-invariant, paramagnetic IPT-DMFT equations have no solution with hybridization functions $\Delta$ corresponding to finite-dimensional AIMs.

#### Proposition 3.3

(Non-existence of solutions to the finite-dimensional bath single-site, translation-invariant, paramagnetic IPT-DMFT equations) Apart from the limit cases $U=0$ or $T_{ }=0$, the single-site, translation-invariant, paramagnetic IPT-DMFT equations (8a)–(8b) have no finite-dimensional bath solution, that is no solution $\Delta$ of the form

$$\begin{aligned} \forall z \in \mathbb {C}_+, \quad \Delta (z) = \sum _{k=1}^K \frac{a_k}{z-\varepsilon _k},\quad K\ge 1, \quad a_k > 0, \quad \varepsilon _k \in \mathbb {R}. \end{aligned}$$

This result originates from the fact that an iteration of the IPT-DMFT map increases the bath dimension *K*. It implies that finding a solution to the DMFT equations requires considering infinite-dimensional bath hybridization functions. The appropriate function space can be characterized in terms of Nevanlinna-Riesz measures, as will be shown in the subsequent section.

#### Remark 3.4

*(Limit cases* $U_{ }=0$ *or* $T_{ }=0$ *)*. The limit cases considered in the previous proposition are covered in Proposition A.14, for the exact impurity solver. In each of these limits, the (unique) solution to the DMFT equations provides the exact solution to the quantum many-body problem, which is indeed the Cauchy-Stieltjes transform of a finite, finitely-supported, positive Borel measure. See also Remark 3.4 below for a discussion of the limit case $U_{ }=0$ for infinite graphs of finite degree.

### Functional setting: The Bath Update and IPT Maps

The DMFT map is the composition of the IPT map and the Bath Update (BU) map, which we will study separately. First, we formalize in our setting the definition of the BU map $F^{\textrm{BU}}$. The following proposition extends the result by Lindsey et al. to the case of finite and positive Borel measures of arbitrary support, by using a different approach. These definitions are motivated by the consequences of Proposition 3.3 and the statements of Propositions 3.5 and 3.7.

We introduce the self-energy space

17 $$\begin{aligned} \mathfrak {S}= \left\{ \mathbb {C}_+\ni z\mapsto U^2\int _\mathbb {R}\frac{d\mu (\varepsilon )}{z-\varepsilon } \in \mathbb {C}\; \ \mu \in \mathcal {M}_+(\mathbb {R})\right\} , \end{aligned}$$

which is in one-to-one correspondence with the set $\mathcal {M}_+(\mathbb {R})$ of finite positive Borel measures on $\mathbb {R}$, and the hybridization space

18 $$\begin{aligned} \mathfrak {D}= \left\{ \mathbb {C}_+\ni z \mapsto |W_{ }|^2 \int _\mathbb {R}\frac{d\nu (\varepsilon )}{z-\varepsilon } \in \mathbb {C}\; \ \nu \in \mathcal {P}(\mathbb {R})\right\} , \end{aligned}$$

which is in one-to-one correspondence with the set $\mathcal {P}(\mathbb {R})$ of Borel probability measures on $\mathbb {R}$. These one-to-one correspondences (which are, up to a multiplicative constant, Cauchy-Stieltjes transforms) allow us to focus on the measure spaces $\mathcal {M}_+(\mathbb {R})$ and $\mathcal {P}(\mathbb {R})$, and study the DMFT iteration scheme in terms of measures. The BU map can then be defined as a function $F^{\textrm{BU}}$ between measure spaces as follows.

#### Proposition 3.5

(Bath Update ($F^{\textrm{BU}}: \Sigma \mapsto \Delta$) map in the single-site, translation-invariant, paramagnetic IPT-DMFT framework). Let $\Sigma \in \mathfrak {S}$, and let $\mu$ be the Nevanlinna-Riesz measure associated with $\Sigma$. Then, the hybridization function $\Delta$, given by

19 $$\begin{aligned} \Delta (z)=W\left( z-H^0_\perp - \Sigma (z)\right) ^{-1}W^\dagger , \end{aligned}$$

for $z \in \mathbb {C}_+$ is well-defined. With this definition, $-\Delta$ is a Pick function and there exists a Borel probability measure $\nu \in \mathcal {P}(\mathbb {R})$ such that

$$\begin{aligned} \forall z \in \mathbb {C}_+, \quad \Delta (z)=|W|^2\int _\mathbb {R}\frac{d\nu (\varepsilon )}{z-\varepsilon }. \end{aligned}$$

The Bath Update (BU) map in the single-site, translation-invariant, paramagnetic IPT-DMFT framework is defined as the function $F^{\textrm{BU}}: \mathcal {M}_+(\mathbb {R})\rightarrow \mathcal {P}(\mathbb {R})$ such that

$$\begin{aligned} F^{\textrm{BU}}(\mu ) = \nu , \end{aligned}$$

with

20 $$\begin{aligned} |W|^2\int _\mathbb {R}\frac{d\nu (\varepsilon )}{z-\varepsilon } = W_{ } \left( z - H^0_\perp - U^2\int _\mathbb {R}\frac{d\mu (\varepsilon )}{z-\varepsilon } \right) ^{-1} W_{ }^\dagger . \end{aligned}$$

#### Remark 3.6

*(Bath Update map in the general DMFT formalism).* In Sect. 4.3, we prove an extension of Proposition 3.5 to the general case presented in Appendix A.4, where we do not make the assumptions (single-site, translation-invariant, paramagnetic) under which the DMFT equations reduce to a set of two equations whose unknowns are two scalar-valued functions.

The second step of the DMFT loop is the impurity solver. Within our model, we define the IPT map $F^{\textrm{IPT}}$, which transforms a given hybridization function $\Delta$ into a self-energy $\Sigma$. As well as the Bath Update map, this mapping operates across measure spaces and maps Borel probability measures to finite positive Borel measures.

#### Proposition 3.7

(Definition of the IPT map in the single-site, translation-invariant, paramagnetic framework). Let $\nu \in \mathcal {P}(\mathbb {R})$ and $\Delta \in \mathfrak {D}$ the hybridization function associated with $\nu$: for all $z \in \mathbb {C}_+$,

21 $$\begin{aligned} \Delta (z)= |W_{ }|^2 \int _\mathbb {R}\frac{{d}\nu (\varepsilon )}{z-\varepsilon }. \end{aligned}$$

There exists $\xi \in \mathcal {P}(\mathbb {R})$, such that for all $z\in \mathbb {C}_+$,

22 $$\begin{aligned} \int _\mathbb {R}\frac{{d}\xi (\varepsilon )}{z-\varepsilon } = \frac{1}{z-\Delta (z)}. \end{aligned}$$

Then define

23 $$\begin{aligned} \tilde{\xi }({d}\varepsilon ):= \dfrac{\xi (d\varepsilon )}{1+e^{-\beta \varepsilon }}, \end{aligned}$$

24 $$\begin{aligned} \tilde{\mu }:= \tilde{\xi }*\tilde{\xi }*\tilde{\xi }, \end{aligned}$$

where $*$ is the convolution product, and

25 $$\begin{aligned} \mu (d\varepsilon ):= (1+e^{-\beta \varepsilon })\tilde{\mu }(d\varepsilon ). \end{aligned}$$

Finally, define the self-energy associated with the measure $\mu$ by

26 $$\begin{aligned} \Sigma (z) = U^2\int _\mathbb {R}\frac{d\mu (\varepsilon )}{z-\varepsilon }. \end{aligned}$$

The measure $\mu$ is a positive finite measure: $\mu \in \mathcal {M}_+(\mathbb {R})$, hence $\Sigma \in \mathfrak {S}$, where $\mathfrak {S}$ is defined in (17). The IPT map $F^{\textrm{IPT}}: \mathcal {P}(\mathbb {R})\rightarrow \mathcal {M}_+(\mathbb {R})$ is defined by

$$\begin{aligned} F^{\textrm{IPT}}(\nu ) = \mu . \end{aligned}$$

Moreover, the map $\mathfrak {D}\ni \Delta \mapsto \Sigma \in \mathfrak {S}$ defined by (22)–(26) is continuous with respect to the weak topology of measures, and coincides with the IPT solver defined in Proposition 3.2 on the dense subset of finite-dimensional bath hybridization functions.

Now that we have defined the maps $F^{\textrm{IPT}}$ and $F^{\textrm{BU}}$, we define the single-site, translation-invariant, paramagnetic IPT-DMFT map as

27 $$\begin{aligned} F^{\textrm{DMFT}}:= F^{\textrm{BU}}\circ F^{\textrm{IPT}}: \mathcal {P}(\mathbb {R})\rightarrow \mathcal {P}(\mathbb {R}). \end{aligned}$$

For the sake of brevity, we will sometimes refer to $F^{\textrm{DMFT}}$ as the IPT-DMFT map and not restate all the simplifying assumptions considered in this analysis: single-site, translation-invariant, paramagnetic framework. In the same way, we will refer to fixed points of $F^{\textrm{DMFT}}$ as IPT-DMFT solutions. Such fixed points (more precisely the associated hybridization functions and self-energies) are indeed solutions of the single-site, translation-invariant, paramagnetic IPT-DMFT equations (8a)–(8b).

### Existence and Properties of IPT-DMFT Solutions

The main result of this paper is the existence of a solution to the DMFT equations (8a)–(8b) in the single-site, translation-invariant, paramagnetic framework, using the IPT impurity solver.

#### Theorem 3.8

(Existence of a fixed-point). The IPT-DMFT map $F^{\textrm{DMFT}}$ has a fixed point $\nu \in \mathcal {P}(\mathbb {R})$.

Moreover, IPT-DMFT solutions that are fixed-points of $F^{\textrm{DMFT}}$ have finite moments at all orders.

#### Proposition 3.9

Let $\nu ^0 \in \mathcal {P}(\mathbb {R})$, $\nu =F^{\textrm{DMFT}}(\nu ^0)$, and $k\in 2\mathbb {N}$. If $\nu ^0\in \mathcal {P}(\mathbb {R})$ has finite *k*-th moment, then $\nu$ has finite $(k+4)$-th moment. In particular, any fixed point of the IPT-DMFT map has finite moments of all orders.

This property of the DMFT map is actually one of the key elements of the proof of Theorem 3.8, as it induces compactness. The fact that a fixed point $\nu$ of the DMFT map $F^{\textrm{DMFT}}$ has finite moments of any order also raises the following question: are there constants $A,\alpha >0$ such that, for all $R \in \mathbb {R}$,

28 $$\begin{aligned} \nu (\mathbb {R}\setminus [-R,R]) \le A e^{-\alpha R} \quad ? \end{aligned}$$

A positive answer to this question would imply that $F^{\textrm{DMFT}}$ has a unique fixed point at high temperature (in the limit case $\beta =0$). The argument is the following. Using Theorem 4.1 and some simple algebraic manipulations of equations (19), (21)–(26), it can be shown that there exists a sequence $(m_n^*)_{n \in \mathbb {N}} \in \mathbb {R}^\mathbb {N}$, depending on $\mathcal {G}_{{H}},T_{ }$ and $U_{ }$, and whose terms can be computed recursively, such that any fixed point $\nu$ of $F^{\textrm{DMFT}}$ for $\beta =0$ satisfies

29 $$\begin{aligned} \forall n \in \mathbb {N}, \quad m_n(\nu )=m^*_{n}, \end{aligned}$$

where we recall that $m_n(\nu )$ is the *n*-th moment of $\nu$. More precisely, fix $k\ge 0$ and $\nu ,\xi ,\mu$ defined as in Proposition 3.7 for a solution of the IPT-DMFT equations. Given equation (22) and using Theorem 4.1, there exists a polynomial $P^1_k\in \mathbb {R}[X_0,\dots ,X_k]$ such that $m_{k+2}(\xi )=P^1_k(m_0(\nu ),\dots ,m_k(\nu ))$. Then, using equations (23)–(25), there exists another polynomial $P^2_k\in \mathbb {R}[X_0,\dots ,X_k]$ such that $m_k(\mu )=P^2_k(m_0(\xi ),\dots ,m_k(\xi ))$. Finally, given equation (26) and (19), and using Theorem 4.1 once again, there exists $P^3_k\in \mathbb {R}[X_0,\dots ,X_k]$ such that $m_{k+2}(\nu )=P^3_k(m_0(\mu ),\dots ,m_k(\mu ))$. Putting these considerations together yields the existence of a polynomial $P_k\in \mathbb {R}[X_0,\dots ,X_k]$, such that $m_{k+4}(\nu )=P_k(m_0(\nu ),\dots ,m_k(\nu ))$. These polynomials can be computed explicitly and are actually independent of $\nu ,\xi ,\mu$, so that $P_k$ is independent of $\nu$ (but it depends on the global parameters $\mathcal {G}_{{H}},T$ and *U*). Additionally, the same reasoning and a direct computation (see Lemma 4.3) yield that the first moments $m_j(\nu )$ for $0\le j\le 3$ are also independent of the chosen solution $\nu$. In other words, by induction, any fixed point of the IPT-DMFT map satisfies the *Hamburger moment problem* (29) defined by the sequence $\left( m_n^*\right) _{n \in \mathbb {N}}$. Now, if $\nu$ satisfies the exponential decay property (28), it is easy to show that $m_n^*=m_n(\nu )$ satisfies the following Carleman’s condition [2]

30 $$\begin{aligned} \sum _{n=1}^\infty \left( m_{2n}^*\right) ^{-\frac{1}{2n}}=+\infty , \end{aligned}$$

which implies that the Hamburger moment problem has a unique solution, hence, that $\nu$ is unique.

Numerical simulations on the Hubbard dimer at zero temperature seem to indicate that (30) holds true for this particular system. However, even in this simple setting, we were unable to prove that $F^\textrm{DMFT}$ has at least one fixed point $\nu$ satisfying the exponential decay (28), nor that the Hamburger moment problem defined by $(m_n^*)_{n \in \mathbb {N}}$ is well-posed.

### Extension of the formalism to countably infinite graphs with finite degree

Many of our results extend to undirected, loopless and countably infinite graphs with finite degree, meaning that the number of neighbors of a site is uniformly bounded by a finite integer, i.e. $\deg (\mathcal {G}_{{H}}):=\sup _{\lambda \in \Lambda } \# \{ \text{ neighbors } \text{ of } \lambda \} <+\infty$. These graphs are indeed locally finite and include the nearest neighbor graphs induced by the *d*-dimensional hypercubic lattices, the triangular lattice, and all the regular trees of finite degree. For such a graph $\mathcal {G}_{{H}}=(\Lambda ,E)$, the formalism, based on the theory of adjacency operators introduced in [54, 55] for locally finite graphs, can be extended as follows under the assumption that the graph is vertex transitive (a property fulfilled by all the aforementioned examples), in the single-site and paramagnetic setting. Let $l^2(\Lambda )$ be the space of sequences indexed by $\Lambda$ which are of finite 2-norm (for all $\phi \in l^2(\Lambda ), \Vert {\phi } \Vert _2^2=\sum _{\lambda \in \Lambda } |\phi _\lambda |^2 < +\infty$). The adjacency operator $\textrm{Adj}_{\mathcal {G}_{{H}}} \in \mathcal {B}(l^2(\Lambda ))$ is the bounded operator defined on $l^2(\Lambda )$ by linearity with, for all $\lambda \in \Lambda$,

$$\begin{aligned} \textrm{Adj}_{\mathcal {G}_{{H}}} e_\lambda = \sum _{\{\lambda ,\lambda '\}\in E} e_{\lambda '}, \quad \text {where } e_\lambda \in l^2(\Lambda ) \text { is defined by } (e_\lambda )_{\lambda '}=\delta _{\lambda \lambda '}. \end{aligned}$$

Defined as such, this operator is indeed bounded ($\Vert {\textrm{Adj}_{\mathcal {G}_{{H}}}} \Vert \le \textrm{deg}(\mathcal {G}_{{H}})$) and symmetric, hence self-adjoint on $l^2(\Lambda )$. In particular, for any $T_{ } \in \mathbb {R}$ and any Pick function *f*, we have that

$$\begin{aligned} z\mapsto -W\left( z-H^0_\perp +f(z)\right) ^{-1}W^\dagger \end{aligned}$$

defines a Pick function, where $W \in l^2(\Lambda ) \setminus \{0\}$ and $H^0_\perp \in \mathcal {S}(\ell ^2(\Lambda \setminus \{0\}))$ are defined similarly as in the finite dimensional case. This fact, together with Theorem 4.1, shows that the proof of Proposition 3.5 extends easily to infinite graphs of finite degree.

Note that Proposition 3.3 also holds, with the slight difference that for the limit case $U_{ }=0$, the solution, being given by $\Sigma =0$ and

$$\begin{aligned} \forall z \in \mathbb {C}_+, \quad \Delta (z)=W\left( z-H^0_\perp \right) ^{-1}W^\dagger , \end{aligned}$$

may not be associated with a finitely supported measure $\nu$, the latter being given by

$$\begin{aligned} \nu =W\pi _{H^0_\perp }W^\dagger , \end{aligned}$$

where $\pi _{H^0_\perp }$ is the spectral measure associated with $H^0_\perp$.

For example, the Nevanlinna-Riesz measure $\nu$ of the hybridization function $\Delta :z \mapsto W\left( z-H^0_\perp \right) W^\dagger$ is, for the nearest neighbor graph of the 1d-lattice $\mathbb {Z},$ within the single-site DMFT approach, absolutely continuous with respect to the Lebesgue measure $d\varepsilon$, and with density $\rho$ given, for all $\varepsilon \in \mathbb {R}$, by

$$\begin{aligned} \rho (\varepsilon )=\lim _{\eta \rightarrow 0^+} \Im \left( \left( \int _{-2}^2 \frac{1}{\varepsilon + i\eta - T_{ }\lambda }\frac{1}{\sqrt{4-\lambda ^2}}d\lambda \right) ^{-1}\right) 1\!\!1_{{[-2T_{ },2T_{ }]}}(\varepsilon ). \end{aligned}$$

This result follows from a classical spectral graph result concerning the 1d-chain [55], in combination with the Stieltjes inversion formula [30], after noting that we have, using Schur complement, for all $z \in \mathbb {C}_+$,

$$\begin{aligned} \Delta (z)=z - \left( \langle e_0,\left( z-T\textrm{Adj}_{\mathcal {G}_{{H}}}\right) ^{-1}e_0 \rangle \right) ^{-1}. \end{aligned}$$

Finally, let us add that the proofs of Theorem 3.8 and Proposition 3.9 extend also to infinite graphs with finite degrees.

The reader familiar with the mathematical results regarding thermodynamic limits of fermion lattice systems may be surprised by the way the results stated in this article extend so straightforwardly to infinite systems. All these results hold because of the way DMFT takes interaction between fermions into account: within this approximation, only equation (8a) depends on the size of the system, which involves only the one-body Hamiltonian and one-body quantities (self-energy, hybridization function).

## Proofs

In this section, we give the proofs of the results stated in Sect. 3. Among other things, we will make use of the results already stated about Pick functions, especially Theorem 3.1 and of some results from measure theory, which will be recalled when needed. As we will discuss continuity of functions defined on measure spaces, we must specify the topology we are considering. Recall that a sequence $(\mu _n)_{n \in \mathbb {N}}$ of finite Borel measures on $\mathbb {R}$ is said to converge weakly to $\mu$ if for all bounded continuous function $f\in \mathcal {C}_b^0(\mathbb {R})$,

31 $$\begin{aligned} \int _\mathbb {R}f \, d\mu _n \rightarrow \int _\mathbb {R}f \, d\mu . \end{aligned}$$

It converges vaguely to $\mu$ if (31) holds for all $f \in \mathcal {C}_0^0(\mathbb {R})$, the space of continuous functions from $\mathbb {R}$ to $\mathbb {R}$ vanishing at infinity. Weak convergence clearly implies vague convergence, and the converse is also true in $\mathcal {P}(\mathbb {R})$, since $\mathbb {R}$ is locally compact [72, Remark 7.2]. We will also make use of the notions of Wasserstein distance and optimal transportation on $\mathbb {R}$. The Wasserstein 2-distance between two Borel probability measures $\mu$ and $\nu$ on $\mathbb {R}$ is defined by

32 $$\begin{aligned} W_2(\mu ,\nu ):= \left( \underset{\pi \in \Pi (\mu ,\nu )}{\inf }\ \int _{\mathbb {R}^2}|x-y|^2d\pi (x,y)\right) ^{\frac{1}{2}}, \end{aligned}$$

where $\Pi (\mu ,\nu )$ is the set of all couplings of $\mu$ and $\nu$, i.e. of Borel probability measures on $\mathbb {R}\times \mathbb {R}$ whose marginals with respect to the first and second variables are respectively $\mu$ and $\nu$. If $\mu$ and $\nu$ have finite moments of order 2, the infimum in the definition (32) is actually a minimum, and there exists in fact a unique $\pi _{\mu ,\nu } \in \Pi (\mu ,\nu )$ such that $W_2(\mu ,\nu )^2=\int |x-y|^2d\pi _{\mu ,\nu }(x,y)$ [64].

Moreover, we will make an extensive use of the following theorem, which extends to Pick matrices a result on Pick functions, which can be found in [18] and [2, Theorem 3.2.1]. It states that the moments of the Nevanlinna-Riesz measure of a Pick function or matrix are related to its expansion on the imaginary axis at $+\infty$.

### Theorem 4.1

Let $f:\mathbb {C}_+\rightarrow \mathbb {C}^{n\times n}$ be a Pick matrix and $\mu$ its Nevanlinna-Riesz measure. Let $n\in \mathbb {N}$. The function *f* satisfies:

33 $$\begin{aligned} -f(iy) = m_{-2} (iy) + m_{-1} + \frac{m_0}{iy} + \frac{m_1}{(iy)^2} +\frac{m_2}{(iy)^3}+\dots +\frac{m_{2n}}{(iy)^{2n+1}}+o_{y \rightarrow +\infty } \left( \frac{1}{y^{2n+1}}\right) \nonumber \\ \end{aligned}$$

if and only if $\mu$ has finite moments of order less than or equal to 2*n*, i.e. for all $x\in \mathbb {C}^n$, $\langle x,\int _\mathbb {R}|\varepsilon |^kd\mu (\varepsilon )x \rangle <\infty$ for $0\le k\le 2n$. For $0\le k \le 2n$, the coefficient $m_k$ is then the *k*-th moment of $\mu$, i.e. $m_k = \int _\mathbb {R}\varepsilon ^kd\mu (\varepsilon )\in \mathcal {S}_n(\mathbb {C})$.

### Proof

The result for Pick functions can be found in [18] and [2, Theorem 3.2.1]. To extend the result to Pick matrices, it suffices to notice *f* is a Pick matrix if and only if for all $x\in \mathbb {C}^n$, the map $f_x:z\in \mathbb {C}_+\mapsto \langle x,f(z)x \rangle$ is a Pick function. $\square$

### Proof of Proposition 3.2 (IPT Map for Finite-Dimensional Bath)

We have for all $z \in \mathbb {C}_+$,

$$\begin{aligned} (z-\Delta (z))^{-1}=\left( z-H^0_{\textrm{AIM}}\right) ^{-1}_{1,1}, \quad \text { where } H^0_\textrm{AIM}=\left( \begin{array}{lllll} 0&  \sqrt{a_1} &  \sqrt{a_2} & \cdots &  \sqrt{a_K} \\ \sqrt{a_1}& \varepsilon _1 &  0 & \cdots &  0 \\ \sqrt{a_2}&  0 &  \varepsilon _2 &  \ddots & \vdots \\ \vdots &  \vdots &  \ddots &  \ddots &  0 \\ \sqrt{a}_K &  0 &  \ldots &  0 &  \varepsilon _K \end{array}\right) , \end{aligned}$$

which holds true in particular for $z=i\omega _n$. Note that $H^0_{\textrm{AIM}}$ is self-adjoint and that for all $n \in \mathbb {Z}$, using functional calculus,

$$\begin{aligned} \int _{0}^\beta e^{i\omega _n\tau } \frac{-e^{-\tau H^0_{\textrm{AIM}}}}{1+e^{-\beta H^0_{\textrm{AIM}}}}d\tau = \left( i\omega _n - H^0_{\textrm{AIM}}\right) ^{-1}, \end{aligned}$$

so that we can perform explicitly the Fourier summation: for all $\tau \in (0,\beta )$,

34 $$\begin{aligned} \frac{1}{\beta }\sum _{n' \in \mathbb {Z}}e^{-i\omega _{n'}\tau }(i\omega _{n'} - \Delta (i\omega _{n'}))^{-1}=\left( \frac{-e^{-\tau H^0_{\textrm{AIM}}}}{1+e^{-\beta H^0_\textrm{AIM}}}\right) _{1,1}=-\sum _{k=1}^{K+1}\frac{\vert P_{1,k}\vert ^2 e^{-\tau \varepsilon '_k}}{1+e^{-\beta \varepsilon '_k}}, \end{aligned}$$

where $P \in \mathbb {C}^{(K+1)\times (K+1)}$ is a unitary matrix such that $H^0_{\textrm{AIM}}=P\textrm{diag}(\varepsilon '_1,\cdots ,\varepsilon '_{K+1}) P^\dagger$. The right-hand side of (34) is a continuous function on $[0,\beta )$ and the following integral is well-defined and reads

$$\begin{aligned} \int _{0}^\beta e^{i\omega _n\tau }\left( \frac{1}{\beta }\sum _{n'\in \mathbb {Z}}\left( i\omega _{n'} - \Delta (i\omega _{n'})\right) ^{-1}e^{-i\omega _{n'}\tau }\right) ^{3} d\tau =&\\ \sum _{k_1,k_2,k_3=1}^{K+1}\frac{1+e^{-\beta (\varepsilon '_{k_1}+\varepsilon '_{k_2}+\varepsilon _{k_3})}}{(1+e^{-\beta \varepsilon '_{k_1}})(1+e^{-\beta \varepsilon '_{k_2}})(1+e^{-\beta \varepsilon '_{k_3}})}&\frac{\vert P_{1,k_1}\vert ^2 \vert P_{1,k_2}\vert ^2 \vert P_{1,k_3}\vert ^2 }{i\omega _{n}-(\varepsilon '_{k_1}+\varepsilon '_{k_2}+\varepsilon '_{k_3})}. \end{aligned}$$

Let us now compute the spectrum of $H^0_{\textrm{AIM}}$: a simple calculation shows that its characteristic polynomial $\chi _{H^0_\textrm{AIM}}$ reads

$$\begin{aligned} \chi _{H^0_{\textrm{AIM}}}(\varepsilon )=\left( \prod _{k=1}^K(\varepsilon -\varepsilon _k)\right) \varepsilon - \sum _{k=1}^K a_k \prod _{l=1,l\ne k}^K (\varepsilon -\varepsilon _l). \end{aligned}$$

By assumption on the $a_k$’s and $\varepsilon _k$’s, we have $\chi _{H^0_{\textrm{AIM}}}(\varepsilon _k)\ne 0$, so that

$$\begin{aligned} \chi _{H^0_{\textrm{AIM}}}(\varepsilon )=0 \iff \varepsilon -\Delta (\varepsilon )=0. \end{aligned}$$

This in fact straightforwardly follows from the Schur complement approach. Moreover, one can compute explicitly $\vert P_{1,k}\vert ^2$: by definition, we have for all $k \in [\![ {1},{K+1}]\!]$ and $l \in [\![ {1},{K}]\!]$,

$$\begin{aligned} \sqrt{a_l}P_{1,k} + \varepsilon _l P_{l+1,k}=\varepsilon '_{k} P_{l+1,k} \implies \frac{a_l}{(\varepsilon '_k-\varepsilon _l)^2}\vert P_{1,k} \vert ^2 = \vert P_{l+1,k} \vert ^2, \end{aligned}$$

which gives after summation on *l* and using the fact that $P P^\dagger =1$,

$$\begin{aligned} \vert P_{1,k} \vert ^2 =(1-\Delta '(\varepsilon '_k))^{-1}. \end{aligned}$$

This shows that $\Sigma ^{\mathrm {{IPT}}}(i\omega _n)=\Sigma _{n}$, hence $\Sigma ^{\mathrm {{IPT}}}$ is a solution to the Analytic Continuation Problem (ACP) defined in (14). Then, Theorem B.9 ensures that there is no other solution, which concludes the proof.

### Proof of Proposition 3.3 (No Finite-Dimensional Bath Solution)

In this section, we prove the statement of Proposition 3.3, which states that there is no solution to the IPT-DMFT equations considering only hybridization functions with a finite-dimensional bath. The idea is simply that the DMFT loop imposes an increase in the number of poles of a hybridization function corresponding to a finite-dimensional bath, which leads to a contradiction if it is supposed to be a solution to the equations.

Suppose $\Delta$ is a hybridization function associated with a bath of finite dimension and a solution to the IPT-DMFT equations. In particular, there exist $K\ge 1$, $a_1,\dots ,a_K>0$ and $\epsilon _{{1}}<\dots <\epsilon _{{K}}$ such that

$$\begin{aligned} \Delta (z) = \sum _{k=1}^K \frac{a_k}{z-\epsilon _{{k}}}. \end{aligned}$$

Let $\Sigma$ be the self-energy given by the IPT impurity solver, see Proposition 3.2. We have

$$\begin{aligned} \Sigma (z) =U^2\sum _{1\le k_1,k_2,k_3\le K'} \frac{a_{k_1,k_2,k_3}'}{z-\varepsilon _{k_1}'-\varepsilon _{k_2}'-\varepsilon _{k_3}'} \text { and } \end{aligned}$$

$$\begin{aligned} a_{k_1,k_2,k_3}'=\left( 1+e^{-\beta (\varepsilon _{k_1}'+\varepsilon _{k_2}'+\varepsilon _{k_3}')}\right) \prod _{i=1}^3\frac{1-\Delta '(\varepsilon _{k_i}')}{1+e^{-\beta \varepsilon _{k_i}'}} > 0, \end{aligned}$$

where $\varepsilon _1'<\dots <\varepsilon _{K'}'$ are the poles of the rational function $(z-\Delta (z))^{-1}$. Since $\Delta$ has exactly *K* real poles, he number of poles of $(z-\Delta (z))^{-1}$ is exactly $K+1$ and the latter are real numbers, so that $K'=K+1$. Indeed, the $K'$ poles are the fixed points of the real-valued function $\varepsilon \in \mathbb {R}\mapsto \Delta (\varepsilon )$. This function is strictly decreasing on its domain of continuity: $\Delta '(\varepsilon )=-\sum _k \frac{a_k}{(\varepsilon -\varepsilon _k)^2}<0$; it goes to zero at infinity, and it goes to $\pm \infty$ at $\varepsilon _k^\pm$, so that there is exactly one fixed point in each interval $(-\infty ,\varepsilon _1), (\varepsilon _1,\varepsilon _2),\dots ,(\varepsilon _K,+\infty )$. Now, since $U_{ }>0$ by assumption, $\Sigma$ has more than $K+1$ poles (we do not need a better estimation of the number of poles). Hence we can write

$$\begin{aligned} \Sigma (z) = U^2\sum _{k=1}^{K''} \frac{a_k''}{z-\varepsilon _k''}, \end{aligned}$$

with $K''\ge K+1$, $a_k''>0$ and $\varepsilon _1<\dots <\varepsilon _{K''}$. As $\Delta$ is assumed to be a solution to the IPT-DMFT equations, it reads

$$\begin{aligned} \Delta (z) = W \left( z-H^0_\perp -\Sigma (z)\right) ^{-1}W^\dagger . \end{aligned}$$

This expression can be written using the spectral decomposition of the matrix $H^0_\perp$:

$$\begin{aligned} \Delta (z) = \sum _{\lambda \in \textrm{Sp}(H^0_\perp )}\frac{|\Pi _\lambda W^\dagger |^2}{z-\lambda -\Sigma (z)}. \end{aligned}$$

Since $W\ne 0$ because $T_{ }> 0$ and due to the hypotheses on the Hubbard graph, there is at least one $\lambda \in \textrm{Sp}(H^0_\perp )$ such that $|\Pi _\lambda W^\dagger |>0$. The same argument as above proves that the associated term in the previous expression is a rational function with exactly $K''+1$ real poles. There is no possible compensation between two same poles in two different terms of the sum because the associated weights are all of the same sign, hence $\Delta$ has more than $K+2$ poles. As *K* is by definition the number of poles of $\Delta$, it is impossible and $\Delta$ cannot be a solution to the IPT-DMFT equations.

### Proof of Proposition 3.5 (Bath Update Map)

As already mentioned in Remark 3.6, we will actually prove an extension of Proposition 3.5 in which we do not restrict ourselves to the single-site, translation-invariant, paramagnetic framework (see Remark 3.6). We consider a finite graph $\Lambda$ of cardinality $L:=|\Lambda | \ge 2$, partitioned into a set of $P \ge 2$ non-overlapping fragments $(\Lambda _p)_{1 \le p \le P}$ with $L_p:=|\Lambda _p| \ge 1$. This leads to an orthogonal decomposition of the one-electron state $\mathcal {H} \cong \mathbb {C}^{2L}$ into

$$\begin{aligned} \mathcal {H} = \mathcal {H}_1 \oplus \cdots \oplus \mathcal {H}_P \quad \text{ with } \quad \mathcal {H}_p \cong \mathbb {C}^{2L_p} \text{ for } \text{ all } 1 \le p \le P. \end{aligned}$$

Identifying $\mathcal {H}_p$ with $\mathbb {C}^{2L_p}$, the non-interacting Hamiltonian matrix in the decomposition $\mathcal {H}_p \oplus \mathcal {H}_p^\perp$ reads

$$\begin{aligned}  &   H_0 \cong \begin{pmatrix} H^0_p &  W_{{p}}\\ W_{{p}}^\dagger &  H^0_{\bar{p}} \end{pmatrix}\\\text{ with } \quad H^0_p  &   \quad \in \mathbb {C}^{2L_p \times 2L_p}, \quad W_{{p}}\in \mathbb {C}^{2(L-L_p) \times 2L_p}, \quad H^0_{\bar{p}} \in \mathbb {C}^{2(L-L_p) \times 2(L-L_p)}, \end{aligned}$$

and the self-energy and hybridization spaces are then respectively given by

35 $$\begin{aligned} \mathfrak {S}_p = \left\{ z\in \mathbb {C}_+\mapsto C+\int _\mathbb {R}\frac{d\mu (\varepsilon )}{z-\varepsilon } \; \ C \in \mathcal {S}_{2L_{p}}(\mathbb {C}), \mu \in \mathcal {M}(\mathbb {R},\mathcal {S}_{2L_{p}}^+(\mathbb {C})) \right\} , \end{aligned}$$

where $\mathcal {M}(\mathbb {R},\mathcal {S}_{n}^+(\mathbb {C}))$ is the set of finite $\mathcal {S}_n^+(\mathbb {C})$-valued Borel measures on $\mathbb {R}$, and

$$\begin{aligned} \mathfrak {D}_p =\left\{ z\in \mathbb {C}_+\mapsto \int _\mathbb {R}\frac{d\nu (\varepsilon )}{z-\varepsilon }\; \ \nu \in \mathcal {M}(\mathbb {R},\mathcal {S}_{2L_{p}}^+(\mathbb {C})), \ \nu (\mathbb {R})=W_{{p}}^\dagger W_{{p}}\right\} . \end{aligned}$$

We refer to Appendix A.4 for more details on this framework.

#### Proposition 4.2

Let $\Sigma = (\Sigma _1,\cdots ,\Sigma _P) \in \mathfrak {S}_1 \times \cdots \times \mathfrak {S}_P$. For each $1\le p\le P$, the hybridization function $\Delta _p$ given by

36 $$\begin{aligned} \forall z\in \mathbb {C}_+, \quad \Delta _p(z)=W_{{p}}\left( z - H^0_{\overline{p}}- \bigoplus _{q\ne p} \Sigma _{q}(z) \right) ^{-1} W_{{p}}^\dagger \end{aligned}$$

is well-defined, $-\Delta _p$ is a Pick matrix and there exists a finite measure $\nu _p\in \mathcal {M}(\mathbb {R},\mathcal {S}_{2L_{p}}^+(\mathbb {C}))$ such that

37 $$\begin{aligned} \forall z \in \mathbb {C}_+, \quad \Delta _p(z) = \int _\mathbb {R}\frac{d\nu _p(\varepsilon )}{z-\varepsilon } \quad \text { and } \quad \nu _p(\mathbb {R}) = W_{{p}}W_{{p}}^\dagger . \end{aligned}$$

In other words, $\Delta _p \in \mathfrak {D}_p$.

#### Proof

Let $C_p\in \mathcal {S}_{2L_{p}}(\mathbb {C})$ and $\mu _p\in \mathcal {M}(\mathbb {R},\mathcal {S}_{2L_{p}}^+(\mathbb {C}))$ be such that for all $z\in \mathbb {C}_+$, $\Sigma _p(z) = C_p +\int _\mathbb {R}\frac{d\mu _p(\varepsilon )}{z-\varepsilon }$. Since for all $1\le p \le P$, $-\Sigma _p: \mathbb {C}_+\rightarrow \mathbb {C}^{2L_{p}\times 2L_{p}}$ is a Pick matrix,

$$\begin{aligned} - \bigoplus _{q \ne p} \Sigma _{q}: \mathbb {C}_+\rightarrow \mathbb {C}^{2(L-L_{p}) \times 2(L-L_{p})} \end{aligned}$$

is a Pick matrix. As $H^0_{\overline{p}}$ is Hermitian, we have for all $z\in \mathbb {C}_+$,

$$\begin{aligned} \Im \left( z - H^0_{\overline{p}}- \bigoplus _{q\ne p} \Sigma _{q}(z)\right) =\Im (z)-\bigoplus _{q \ne p} \Im (\Sigma _{q}(z)) \ge \Im (z) > 0, \end{aligned}$$

where $M_1\ge M_2$ means that $M_1-M_2$ is a positive semidefinite matrix. Moreover, if $\Im (M) > 0$, then *M* is invertible. Thus $z-H^0_{\overline{p}}-\bigoplus _{q\ne j} \Sigma _{q}(z)$ is invertible and $\Delta _p(z)$ is well defined. As $\Im (M)>0$ if and only if $\Im (M^{-1})<0$, and as the congruence preserves the sign of the imaginary part, we have $\Im (\Delta _p(z))\le 0$ for all $z\in \mathbb {C}_+$, so that $-\Delta _p$ is a Pick matrix. To show formula (37), we will make use of the results on Pick functions stated in Sect. 2.1. As $\mu _q$, the Nevanlinna-Riesz measure of $\Sigma _q$, is finite for $1\le q \le P$, we have for $x\in \mathbb {C}^{2L_{q}}$, that the positive Borel measure $\mu _q^x$ defined as the Nevanlinna-Riesz measure of the Pick function $z\mapsto -\langle x,\Sigma _q(z)x \rangle$, is also a finite positive measure. Then, for $x\in \mathbb {C}^{2L_{q}}$ and $y\ge 1$,

$$\begin{aligned}  &   \left| \left\langle x,\int _\mathbb {R}\frac{d\mu _q(\varepsilon )}{iy-\varepsilon }x\right\rangle \right| = \left| \int _\mathbb {R}\frac{d\mu _q^x(\varepsilon )}{iy-\varepsilon }\right| \le \int _\mathbb {R}\frac{d\mu _q^x(\varepsilon )}{|iy-\varepsilon |} \le \int _\mathbb {R}d\mu _q^x < \infty . \end{aligned}$$

This coarse upper bound is enough to ensure that

$$\begin{aligned} iy\Delta _p(iy)  &   = iyW_{{p}}\left( iy-H^0_{\overline{p}}-\bigoplus _{q\ne p} \Sigma _q(iy)\right) ^{-1} W_{{p}}^\dagger \\  &   = W_{{p}}\left( 1-\frac{1}{iy}\left( H^0_{\overline{p}}+\bigoplus _{q\ne p} \left( C_q+\int _\mathbb {R}\frac{d\mu _q(\varepsilon )}{iy-\varepsilon }\right) \right) \right) ^{-1} W_{{p}}^\dagger \\  &   \underset{y\rightarrow +\infty }{\longrightarrow }\ W_{{p}}W_{{p}}^\dagger . \end{aligned}$$

This gives the expansion $\displaystyle \Delta _p(iy) = \frac{W_{{p}}W_{{p}}^\dagger }{iy} + o\left( \frac{1}{y}\right)$, as *y* goes to $+\infty$. By Theorem 4.1, it follows that the Nevanlinna-Riesz measure of $-\Delta _p$, denoted by $\nu _p$, is finite, and its mass is precisely the quantity $W_{{p}}^\dagger W_{{p}}$. Thus the Nevanlinna-Riesz representation of $-\Delta _p$ reads

$$\begin{aligned} -\Delta _p(z) = az+b-\int _\mathbb {R}\frac{d\nu _p(\varepsilon )}{z-\varepsilon }, \end{aligned}$$

for some $a\in \mathcal {S}_{2L_{p}}^+(\mathbb {C})$ and $b\in \mathcal {S}_{2L_{p}}(\mathbb {C})$. Now, because of the aforementioned expansion, we must have $a=b=0$, which concludes the proof. $\square$

### Proof of Proposition 3.7 (IPT Map for Infinite-Dimensional Bath)

Let $\nu \in \mathcal {P}(\mathbb {R})$, and define the associated hybridization function $\Delta (z) = |W|^2\int _\mathbb {R}\frac{d\nu (\varepsilon )}{z-\varepsilon }$. We first establish the following lemma, which we will use several times in our analysis.

#### Lemma 4.3

Let $c \in \mathbb {R}$, $\mu _0\in \mathcal {M}_+(\mathbb {R})$. For $z\in \mathbb {C}_+$, we set

$$\begin{aligned} f(z) = \int _\mathbb {R}\frac{d\mu _0(\varepsilon )}{z-\varepsilon } \quad \text{ and } \quad g(z) = \displaystyle \frac{1}{z-c-f(z)}. \end{aligned}$$

Then $-g$ is a Pick function and its Nevanlinna-Riesz representation reads $g(z)=\int _\mathbb {R}\frac{d\mu (\varepsilon )}{z-\varepsilon }$ with $\mu \in \mathcal {P}(\mathbb {R})$. In particular, $\mu$ has finite moments of order less than or equal to 2, given by

$$\begin{aligned} m_0(\mu )=1 \text{( }\mu \text{ is } \text{ a } \text{ probability } \text{ measure) }, \quad m_1(\mu )=c \quad \text{ and } \quad m_2(\mu )=\mu _0(\mathbb {R})+c^2. \end{aligned}$$

#### Proof

The result follows from the expansion of the function *g*(*z*) when $z=iy$, $y\rightarrow +\infty$ and Theorem 4.1. One has

$$\begin{aligned} g(iy) = \frac{1}{iy}\frac{1}{1-\frac{c}{iy}-\frac{f(iy)}{iy}}. \end{aligned}$$

Since $\displaystyle f(iy)=\frac{\mu _0(\mathbb {R})}{iy}+o\left( \frac{1}{y}\right)$, we have

$$\begin{aligned} g(iy) = \frac{1}{iy}\frac{1}{1-\frac{c}{iy}-\frac{\mu _0(\mathbb {R})}{(iy)^2}+o\left( \frac{1}{y^2}\right) }= &   \frac{1}{iy}\left( 1+\frac{c}{iy}+\frac{\mu _0(\mathbb {R})+c^2}{(iy)^2}+o\left( \frac{1}{y^2}\right) \right) \\= &   \frac{1}{iy}+\frac{c}{(iy)^2}+\frac{\mu _0(\mathbb {R})+c^2}{(iy)^3}+o\left( \frac{1}{y^3}\right) . \end{aligned}$$

Using again Theorem 4.1, we obtain the desired result. $\square$

We apply Lemma 4.3 with $c=0$ and $f=\Delta$. It follows that there exists $\xi \in \mathcal {P}(\mathbb {R})$ satisfying (22). Then, following equations (23)–(25), set $\tilde{\xi }(d\varepsilon ) = \dfrac{\xi (d\varepsilon )}{1+e^{-\beta \varepsilon }}$, and $\tilde{\mu }=\tilde{\xi }*\tilde{\xi }*\tilde{\xi }$. We must verify that, setting $\mu (d\varepsilon ) = (1+e^{-\beta \varepsilon })\tilde{\mu }(d\varepsilon )$, $\mu$ is indeed a positive finite measure, so that the self-energy $\Sigma$ associated with the measure $\mu$ belongs to $\mathfrak {S}^\textrm{IPT}$.

The multiplication by positive functions and the convolution preserves positivity, so $\mu$ is a positive measure. Moreover, we have

38 $$\begin{aligned} \mu (\mathbb {R}) = \int _{\mathbb {R}^3} \frac{\left( 1+e^{-\beta (\varepsilon _1+\varepsilon _2+\varepsilon _3)}\right) d\xi (\varepsilon _1)d\xi (\varepsilon _2)d\xi (\varepsilon _3)}{(1+e^{-\beta \varepsilon _1})(1+e^{-\beta \varepsilon _2})(1+e^{-\beta \varepsilon _3})}\le \int _{\mathbb {R}^3} d\xi (\varepsilon _1)d\xi (\varepsilon _2)d\xi (\varepsilon _3) = 1. \nonumber \\ \end{aligned}$$

Thus $\mu$ is finite, and the map $F^{\textrm{IPT}}$ is well defined. To prove that this map is actually continuous with respect to the weak topology of measures, we need to establish Lemma 4.4. This result states the continuity of the map $\mathcal {M}_+(\mathbb {R})\ni \mu _0 \mapsto \mu \in \mathcal {P}(\mathbb {R})$ defined in Lemma 4.3, which is central in the IPT-DMFT equations. Note that it holds

39 $$\begin{aligned} \forall z\in \mathbb {C}_+, \quad \int _\mathbb {R}\frac{d\mu (\varepsilon )}{z-\varepsilon } = \frac{1}{z-c-\int _\mathbb {R}\frac{d\mu _0(\varepsilon )}{z-\varepsilon }}. \end{aligned}$$

#### Lemma 4.4

The map $\Phi :\mathcal {M}_+(\mathbb {R})\ni \mu _0 \mapsto \mu \in \mathcal {P}(\mathbb {R})$ defined in Lemma 4.3 is weakly continuous. More precisely, the following stronger result holds true: if $(\mu _0^n)_{n \in \mathbb {N}}$ converges weakly to $\mu _0$ in $\mathcal {M}_+(\mathbb {R})$, then $W_2(\Phi (\mu _0^n),\Phi (\mu ))\underset{n\rightarrow \infty }{\longrightarrow }0$, where $W_2$ is the Wasserstein distance of order 2.

#### Proof

Let $(\mu _0^n)_{n \in \mathbb {N}} \subset \mathcal {M}_+(\mathbb {R})$ converging weakly to $\mu _0$ in $\mathcal {M}_+(\mathbb {R})$, $\mu ^n:=\Phi (\mu ^n_0)$ and $\mu :=\Phi (\mu _0)$. In view of Lemma 4.3, $\mu ^n$ has finite moments of orders 1 and 2, given by $m_1(\mu ^n)=c$ and $m_2(\mu ^n)=\mu _0^n(\mathbb {R})+c^2$.

As $\mu _0^n$ converges weakly to $\mu _0$, we have $\int _\mathbb {R}f d\mu _0^n \rightarrow \int _\mathbb {R}f d\mu _0$ for all $f \in \mathcal {C}_b^0(\mathbb {R})$. Taking $f \equiv 1$, we get $\mu _0^n(\mathbb {R}) = \int _\mathbb {R}d\mu _0^n \underset{n\rightarrow \infty }{\longrightarrow }\ \int _\mathbb {R}d\mu _0 = \mu _0(\mathbb {R})$. Hence, $m_2(\mu ^n)=\mu _0^n(\mathbb {R})+c^2\underset{n\rightarrow \infty }{\longrightarrow }\ \mu _0(\mathbb {R})+c^2=m_2(\mu )$.

Now, for all $z\in \mathbb {C}_+$, the function $\mathbb {R}\ni \varepsilon \mapsto \dfrac{1}{z-\varepsilon } \in \mathbb {C}$ is also bounded and continuous. Thus, we can pass to the limit in formula (39):

40 $$\begin{aligned} \int _\mathbb {R}\frac{d\mu ^n(\varepsilon )}{z-\varepsilon } = \frac{1}{z-c-\int _\mathbb {R}\frac{d\mu _0^n(\varepsilon )}{z-\varepsilon }}\underset{n\rightarrow \infty }{\longrightarrow }\ \frac{1}{z-c-\int _\mathbb {R}\frac{d\mu _0(\varepsilon )}{z-\varepsilon }} = \int _\mathbb {R}\frac{d\mu (\varepsilon )}{z-\varepsilon }. \end{aligned}$$

This can be extended to complex numbers $z\in \mathbb {C}\setminus \mathbb {R}$ by taking the complex conjugate of the limit (40). Let $\mathcal {A}$ be the algebra generated by the functions $\displaystyle \mathbb {R}\ni \varepsilon \mapsto \frac{1}{z-\varepsilon } \in \mathbb {C}$ for $z\in \mathbb {C}\setminus \mathbb {R}$. It is known that $\mathcal {A}$ is dense in $\mathcal {C}_0^0(\mathbb {R})$ (this can be shown using Helffer-Sjöstrand formula [35]). Together with (40), this implies that for all $f\in \mathcal {C}_0^0(\mathbb {R})$, $\int _\mathbb {R}fd\mu ^n \underset{n\rightarrow \infty }{\longrightarrow }\int _\mathbb {R}fd\mu$, i.e. that $(\mu ^n)_{n \in \mathbb {N}}$ vaguely converges to $\mu$. Since $\mathbb {R}$ is locally compact, and because $\mu$ and the $\mu ^n$’s are probability measures, the vague convergence is equivalent in this case to the weak convergence. The map $\Phi$ is therefore weakly continuous. Since on the space of Borel probability measures on $\mathbb {R}$, $W_2$-convergence is equivalent to weak convergence and the convergence of the second moment [3, Section 7.1], the proof is complete. $\square$

Let us now prove that the impurity solver $F^{\textrm{IPT}}: \mathcal {P}(\mathbb {R})\rightarrow \mathcal {M}_+(\mathbb {R})$ is weakly continuous. Let $(\nu ^n)_{n \in \mathbb {N}} \subset \mathcal {P}(\mathbb {R})$ converging weakly to $\nu \in \mathcal {P}(\mathbb {R})$. Define $\xi ^n\in \mathcal {P}(\mathbb {R})$ and $\mu ^n:=F^{\textrm{IPT}}(\nu ^n)$ as in Proposition 3.7, see equation (22), as well as $\xi \in \mathcal {P}(\mathbb {R})$ and $\mu :=F^{\textrm{IPT}}(\nu )$. We want to show that $\mu ^n$ converges weakly to $\mu$. First, because of the definition of $\xi$ through equation (22), and thanks to Lemma 4.4, we know that $W_2(\xi ^n,\xi )\underset{n\rightarrow \infty }{\longrightarrow }0$. Moreover, using the same density argument as in the proof of Lemma 4.4, it is sufficient to show that for all $z\in \mathbb {C}_+$, the following convergence holds:

$$\begin{aligned} \int _\mathbb {R}\frac{d\mu ^n(\varepsilon )}{z-\varepsilon }\underset{n\rightarrow \infty }{\longrightarrow }\int _\mathbb {R}\frac{d\mu (\varepsilon )}{z-\varepsilon }. \end{aligned}$$

We have for $z\in \mathbb {C}_+$,

$$\begin{aligned} \int _\mathbb {R}\frac{d\mu ^n(\varepsilon )}{z-\varepsilon } = \int _{\mathbb {R}^3} \frac{1}{z-(\varepsilon _1+\varepsilon _2+\varepsilon _3)}\frac{1+e^{-\beta (\varepsilon _1+\varepsilon _2+\varepsilon _3)}}{(1+e^{-\beta \varepsilon _1})(1+e^{-\beta \varepsilon _2})(1+e^{-\beta \varepsilon _3})}d\xi ^n(\varepsilon _1)d\xi ^n(\varepsilon _2)d\xi ^n(\varepsilon _3). \end{aligned}$$

Let $\displaystyle \psi (\varepsilon _1,\varepsilon _2,\varepsilon _3) = \frac{1}{z-(\varepsilon _1+\varepsilon _2+\varepsilon _3)}\frac{1+e^{-\beta (\varepsilon _1+\varepsilon _2+\varepsilon _3)}}{(1+e^{-\beta \varepsilon _1})(1+e^{-\beta \varepsilon _2})(1+e^{-\beta \varepsilon _3})}$, for $(\varepsilon _1,\varepsilon _2,\varepsilon _3)\in \mathbb {R}^3$. Then,

41 $$\begin{aligned} \left| \int _\mathbb {R}\frac{d\mu ^n(\varepsilon )}{z-\varepsilon }-\int _\mathbb {R}\frac{d\mu (\varepsilon )}{z-\varepsilon }\right|= &   \left| \int _{\mathbb {R}^3} \psi d\xi ^nd\xi ^nd\xi ^n - \int _{\mathbb {R}^3} \psi d\xi d\xi d\xi \right| \nonumber \\\le &   \left| \int _{\mathbb {R}^3} \psi d\xi ^nd\xi ^nd\xi ^n - \int _{\mathbb {R}^3} \psi d\xi ^n d\xi ^n d\xi \right| \end{aligned}$$

42 $$\begin{aligned}+ &   \left| \int _{\mathbb {R}^3} \psi d\xi ^nd\xi ^nd\xi - \int _{\mathbb {R}^3} \psi d\xi ^n d\xi d\xi \right| \end{aligned}$$

43 $$\begin{aligned}+ &   \left| \int _{\mathbb {R}^3} \psi d\xi ^nd\xi d\xi - \int _{\mathbb {R}^3} \psi d\xi d\xi d\xi \right| . \end{aligned}$$

We now prove that the last term (43) goes to zero when *n* goes to $\infty$. The same arguments apply to the other two terms, (41) and (42). The function $\psi$ is smooth and a simple calculation shows that its partial derivative with respect to $\varepsilon _1$ is bounded by

44 $$\begin{aligned} |\partial _1\psi (\varepsilon _1,\varepsilon _2,\varepsilon _3)| \le \frac{1}{|\Im (z)|^2}+\frac{2\beta }{|\Im (z)|}=:\kappa _{z,\beta }. \end{aligned}$$

Let $\varepsilon _2,\varepsilon _3\in \mathbb {R}$. Using the $W_2$ convergence of $\xi ^n$ towards $\xi$, set $\pi _n$ the optimal coupling between these two measures [64]. We have that

$$\begin{aligned} \int _\mathbb {R}\psi (\varepsilon _1,\varepsilon _2,\varepsilon _3)d\xi ^n(\varepsilon _1) = \int _{\mathbb {R}^2} \psi (\varepsilon _1,\varepsilon _2,\varepsilon _3) d\pi _n(\varepsilon _1,\varepsilon _1'). \end{aligned}$$

By Taylor’s Theorem and since $\psi$ is continuously derivable, we have

$$\begin{aligned}  &   \int _\mathbb {R}\psi (\varepsilon _1,\varepsilon _2,\varepsilon _3)d\xi ^n(\varepsilon _1) = \int _{\mathbb {R}^2} \psi (\varepsilon _1',\varepsilon _2,\varepsilon _3) d\pi _n(\varepsilon _1,\varepsilon _1') \\  &   + \int _{\mathbb {R}^2} \int _{\varepsilon _1'}^{\varepsilon _1}(\varepsilon _1-t) \partial _1\psi (t,\varepsilon _2,\varepsilon _3) dt d\pi _n(\varepsilon _1,\varepsilon _1'). \end{aligned}$$

On the one hand, the first term is exactly $\int _\mathbb {R}\psi (\varepsilon _1,\varepsilon _2,\varepsilon _3)d\xi (\varepsilon _1)$ by definition of $\pi _n$, and on the other hand, using (44), we can bound the second term by

$$\begin{aligned} \left| \int _{\mathbb {R}^2} \int _{\varepsilon _1'}^{\varepsilon _1}(\varepsilon _1-t) \partial _1\psi (t,\varepsilon _2,\varepsilon _3) dt d\pi _n(\varepsilon _1,\varepsilon _1')\right|\le &   \frac{1}{2}\int _{\mathbb {R}^2} |\varepsilon _1-\varepsilon _1'|^2 \Vert \partial _1\psi \Vert _\infty d\pi _n(\varepsilon _1,\varepsilon _1') \\\le &   \frac{\kappa _{z,\beta }}{2} \int _{\mathbb {R}^2} |\varepsilon _1-\varepsilon _1'|^2 d\pi _n(\varepsilon _1,\varepsilon _1') \\= &   \frac{\kappa _{z,\beta }}{2} W_2(\xi ^n,\xi )^2. \end{aligned}$$

Finally, the term (43) can be bounded by

$$\begin{aligned}&\left| \int _{\mathbb {R}^3} \psi d\xi ^nd\xi d\xi - \int _{\mathbb {R}^3} \psi d\xi d\xi d\xi \right| \\&\quad \le \int _{\mathbb {R}^2}\left| \int _\mathbb {R}\psi (\varepsilon _1,\cdot ,\cdot ) d\xi ^n(\varepsilon _1) - \int _\mathbb {R}\psi (\varepsilon _1,\cdot ,\cdot ) d\xi (\varepsilon _1) \right| d\xi d\xi \\&\quad = \int _{\mathbb {R}^2}\left| \int _{\mathbb {R}^2} \int _{\varepsilon _1'}^{\varepsilon _1}(\varepsilon _1-t) \partial _1\psi (t,\varepsilon _2,\varepsilon _3) dt d\pi _n(\varepsilon _1,\varepsilon _1')\right| d\xi (\varepsilon _2) d\xi (\varepsilon _3) \\&\quad \le \int _{\mathbb {R}^2} \frac{\kappa _{z,\beta }}{2} W_2(\xi ^n,\xi )^2 d\xi (\varepsilon _2) d\xi (\varepsilon _3) = \frac{\kappa _{z,\beta }}{2} W_2(\xi ^n,\xi )^2 \underset{n\rightarrow \infty }{\longrightarrow }0. \end{aligned}$$

This shows that the map $F^{\textrm{IPT}}$ is weakly continuous. It remains to prove that the map $\mathfrak {D}^\textrm{IPT}\ni \Delta \mapsto \Sigma \in \mathfrak {S}^\textrm{IPT}$ defined by (22)–(26) coincides on the set of discrete probability measures with finite support with the map defined in Proposition 3.2.

Let $\Delta \in \mathfrak {D}^{\textrm{IPT}}$ with a Nevanlinna-Riesz measure of the form $\nu =\sum _{k=1}^Ka_k\delta _{\varepsilon _k}$, where $a_k>0$, $\sum a_k = |W|^2$ and $\varepsilon _1<\dots <\varepsilon _K$. It follows that the rational function $(z-\Delta (z))^{-1}$ is of the form $\sum _{k=1}^{K+1} \frac{{a}_k'}{z-{\varepsilon }_k'}$ (see the proof of Proposition 3.3 in Sect. 4.2). This means by (22) that $\xi =\sum _{k=1}^{K+1}{a}_k'\delta _{{\varepsilon }_k'}$, and the ${\varepsilon }_k'$’s are the zeros of the rational function $z-\Delta (z)$, so that the residues are given by ${a}_k'=(1-\Delta '({\varepsilon }_k'))^{-1}$. The self-energy $\Sigma$ given by (26) then reads for all $z\in \mathbb {C}_+$,

$$\begin{aligned} \Sigma (z)&= U^2\int _{\mathbb {R}^3} \frac{1+e^{-\beta (\varepsilon _1+\varepsilon _2+\varepsilon _3)}}{(1+e^{-\beta \varepsilon _1})(1+e^{-\beta \varepsilon _2})(1+e^{-\beta \varepsilon _3})} \frac{d\xi (\varepsilon _1)d\xi (\varepsilon _2)d\xi (\varepsilon _3)}{z-(\varepsilon _1+\varepsilon _2+\varepsilon _3)} \\&= U^2\sum _{k_1,k_2,k_3 = 1}^{K+1} \frac{1+e^{-\beta (\varepsilon '_{k_1}+\varepsilon '_{k_2}+\varepsilon _{k_3})}}{(1+e^{-\beta \varepsilon '_{k_1}})(1+e^{-\beta \varepsilon '_{k_2}})(1+e^{-\beta \varepsilon '_{k_3}})} \frac{a_{k_1}'a_{k_2}'a_{k_3}'}{z-(\varepsilon _{k_1}'+\varepsilon _{k_2}'+\varepsilon _{k_3}')} \\&= U^2\sum _{k_1,k_2,k_3 = 1}^{K+1} \frac{a_{k_1,k_2,k_3}'}{z-(\varepsilon _{k_1}'+\varepsilon _{k_2}'+\varepsilon _{k_3}')}, \end{aligned}$$

where $a_{k_1,k_2,k_3}'$ is given by (16). This complies with the result stated in Proposition 3.2.

Finally, since the set of discrete probability measures with finite support is dense in the set of probability measures $\mathcal {P}(\mathbb {R})$ for the weak topology and since $F^{\textrm{IPT}}$ is weakly continuous, $F^{\textrm{IPT}}$ is the only continuous extension of the IPT map defined in Proposition 3.2 for a finite-dimensional bath.

### Continuity of the IPT-DMFT Map

The following result is central to prove the existence of a fixed point to the DMFT equations.

#### Theorem 4.5

(Continuity of the IPT-DMFT map). The IPT-DMFT map $F^\textrm{DMFT}$ is weakly continuous on $\mathcal {P}(\mathbb {R})$. More precisely, the following stronger results holds true: if $(\nu ^n)_{n \in \mathbb {N}}$ converges weakly to $\nu$, then

$$\begin{aligned} W_2\left( F^{\textrm{DMFT}}(\nu ^n),F^{\textrm{DMFT}}(\nu )\right) \underset{n\rightarrow \infty }{\longrightarrow }0, \end{aligned}$$

where $W_2$ is the Wasserstein 2-distance.

We have proven in the previous section the continuity of the map $F^{\textrm{IPT}}$. In order to prove the continuity of $F^{\textrm{BU}}$, we need to adapt Lemma 4.4 to equation (20). Let $\mu \in \mathcal {M}_+(\mathbb {R})$. Applying Theorem 4.1 to the measure $\mu$, and using the definition (20) of the hybridization function $\Delta$ associated with $F^{\textrm{BU}}(\mu )$, we obtain

$$\begin{aligned} \Delta (iy)= &   \frac{1}{iy} W\left( 1 - \frac{H^0_\perp }{iy} - \frac{1}{iy} U^2 \int _{\mathbb {R}} \frac{d\mu (\varepsilon )}{iy-\varepsilon } \right) ^{-1} W^\dagger \\= &   \frac{1}{iy} W \left( 1 - \frac{H^0_\perp }{iy} - \frac{1}{iy}\left( \frac{U^2\mu (\mathbb {R})}{iy} +o\left( \frac{1}{y}\right) \right) \right) ^{-1} W^\dagger \\= &   W\left[ \frac{1}{iy} + \frac{H^0_\perp }{(iy)^2} + \frac{(H^0_\perp )^2+ U^2 \mu (\mathbb {R})}{(iy)^3}\right] W^\dagger + o\left( \frac{1}{y^3}\right) . \end{aligned}$$

It follows that, when $y\rightarrow +\infty$, we have the expansion

45 $$\begin{aligned} \int _{\mathbb {R}} \frac{dF^{\textrm{BU}}(\mu )(\varepsilon )}{iy-\varepsilon } = \frac{1}{|W|^2}\Delta (iy) = \frac{1}{iy} + \frac{s^1}{(iy)^2}+\frac{s^2(\mu )}{(iy)^3} + o\left( \frac{1}{y^3}\right) , \end{aligned}$$

with $s^1$ and $s^2(\mu )$ given by

46 $$\begin{aligned} s^1 = \frac{WH^0_\perp W^\dagger }{|W|^2}, \end{aligned}$$

47 $$\begin{aligned} s^2(\mu ) = \frac{W\left( (H^0_\perp )^2+ U^2 \mu (\mathbb {R})\right) W^\dagger }{|W|^2}. \end{aligned}$$

In view of Theorem 4.1, this implies that $\nu :=F^{\textrm{BU}}(\mu )$ has finite moments of orders 1 and 2, respectively given by $m_1(\nu )=s^1$ and $m_2(\nu )=s^2(\mu )$. The arguments in the proof of Lemma 4.4 can then be used to show that the following result holds true.

#### Proposition 4.6

$F^{\textrm{BU}}: \mathcal {M}_+(\mathbb {R})\rightarrow \mathcal {P}(\mathbb {R})$ is weakly continuous. More precisely, if $(\mu ^n)_{n \in \mathbb {N}}$ converges weakly to $\mu$ in $\mathcal {M}_+(\mathbb {R})$, then $W_2(F^{\textrm{BU}}(\mu ^n),F^{\textrm{BU}}(\mu ))\underset{n\rightarrow \infty }{\longrightarrow }0$.

We are now in position to complete the proof of Theorem 4.5. Let $(\nu ^n)_n\subset \mathcal {P}(\mathbb {R})$ converging weakly to $\nu$ in $\mathcal {P}(\mathbb {R})$. Denoting by $\mu ^n:=F^{\textrm{IPT}}(\nu ^n)$ and $\mu :=F^{\textrm{IPT}}(\nu )$, we have shown in the proof of Proposition 3.7 that $\mu ^n$ converges weakly to $\mu$ in $\mathcal {M}_+(\mathbb {R})$, see Sect. 4.4. Proposition 4.6 then shows that $W_2(F^{\textrm{DMFT}}(\nu ^n),F^{\textrm{DMFT}}(\nu )) = W_2(F^{\textrm{BU}}(\mu ^n),F^{\textrm{BU}}(\mu ))\underset{n\rightarrow \infty }{\longrightarrow }0$. In particular, $F^{\textrm{DMFT}}(\nu ^n)$ converges weakly to $F^{\textrm{DMFT}}(\nu )$.

### Proof of Theorem 3.8 (Existence of a Fixed Point)

The existence of a fixed point to the IPT-DMFT map, that is a solution to the IPT-DMFT equations, is a consequence of the following fixed point theorem [68].

#### Theorem 4.7

(Schauder-Singbal). Let *E* be a locally convex Hausdorff linear topological space, *C* a nonempty closed convex subset of *E*, and *F* a continuous map from *C* into itself, such that *F*(*C*) is contained in a compact subset of *C*. Then *F* has a fixed point.

Let us consider the vector space $E = \mathcal {M}(\mathbb {R})$ endowed with the Kantorovitch-Rubinstein norm $\Vert \cdot \Vert _0$. Recall that the latter is defined as

$$\begin{aligned} \Vert \mu \Vert _0:= \sup \left\{ \int _\mathbb {R}f d\mu \; \ f\in \textrm{Lip}_1, \Vert f\Vert _\infty \le 1 \right\} , \end{aligned}$$

where $\textrm{Lip}_1$ is the set of continuous functions on $\mathbb {R}$ with Lipschitz constant less than or equal to 1.

Let us then set $C:= \mathcal {P}(\mathbb {R})= \{\mu \in E \ | \ \mu \ge 0, \ \int _\mathbb {R}d\mu = 1 \}$. Since *E* is a normed vector space on $\mathbb {R}$, it is a locally convex Hausdorff linear topological space, and *C* is obviously a non-empty convex subset of *E*.

Besides, on the set of finite positive measures, weak convergence is equivalent to convergence for the Kantorovitch-Rubinstein norm [10, Chapter 8.3]. We can thus work with the weak topology on *C*.

The fact that *C* is weakly closed means that $\mathcal {P}(\mathbb {R})$ is a weakly closed subset of $\mathcal {M}(\mathbb {R})$. This result can be found in [11, Section 3.2]. Moreover, we already proved in Theorem 4.5 that $F^{\textrm{DMFT}}: C \rightarrow C$ was weakly continuous.

To apply Schauder-Singbal’s theorem to our setting, it thus remains to show that $F^{\textrm{DMFT}}(C)$ is relatively compact for the weak topology. This is in fact a consequence of Prokhorov’s Theorem [11, Theorem 2.3.4].

Indeed, let $\nu \in F^{\textrm{DMFT}}(C)$ and $\nu _0\in \mathcal {P}(\mathbb {R})$, $\mu =F^{\textrm{IPT}}(\nu _0)\in \mathcal {M}_+(\mathbb {R})$ such that $\nu =F^{\textrm{DMFT}}(\nu _0)=F^{\textrm{BU}}(\mu )$. As we have seen in the proof of Proposition 4.6, $\nu$ has finite moments of order 1 and 2, given respectively by $m_1(\nu )=s^1$ and $m_2(\nu )=s^2(\mu )$, where $s^1$ is defined by (46) and $s^2(\mu )$ by (47). The inequality (38) states that the mass of the measure $\mu$ is bounded by 1. Hence, $m_2(\nu )=s^2(\mu )$ is bounded independently on $\nu$:

48 $$\begin{aligned} m_2(\nu ) = s^2(\mu )\le c:= \frac{W\left( (H^0_\perp )^2+ U^2 \right) W^\dagger }{|W|^2}. \end{aligned}$$

This allows us to show that $F^{\textrm{DMFT}}(C)$ is tight. For $\eta >0$, take $K=[-a,a]$, with $a\ge 1$ large enough so that $c/a^2\le \eta$. Then for $\nu \in F^{\textrm{DMFT}}(C)$,  (48) holds and

$$\begin{aligned} \nu (\mathbb {R}\setminus K) = \int _{\mathbb {R}\setminus K}\frac{\varepsilon ^2}{\varepsilon ^2}d\nu (\varepsilon )\le \frac{1}{a^2} \int _{\mathbb {R}\setminus K}\varepsilon ^2d\nu (\varepsilon ) \le \frac{1}{a^2}\int _\mathbb {R}\varepsilon ^2d\nu (\varepsilon ) = \frac{m_2(\nu )}{a^2} \le \frac{c}{a^2} \le \eta . \end{aligned}$$

Hence $F^{\textrm{DMFT}}(C)$ is tight. By Prokhorov’s Theorem, it is thus weakly relatively compact.

This concludes the proof of our main result.

### Proof of Proposition 3.9 (Finiteness of the Moments of a Solution)

Let $\nu ^0\in \mathcal {P}(\mathbb {R})$ and $k\in 2\mathbb {N}$, and assume that $\nu ^0$ has finite *k*-th moment, i.e. $\int _\mathbb {R}|\varepsilon |^kd\nu ^0(\varepsilon )<\infty$. By Theorem 4.1, the following expansion holds:

49 $$\begin{aligned} \int _{\mathbb {R}}\frac{d\nu ^0(\varepsilon )}{iy-\varepsilon } = \frac{1}{iy}+\dots +\frac{m_k(\nu ^0)}{(iy)^{k+1}}+o\left( \frac{1}{y^{k+1}}\right) . \end{aligned}$$

Then define $\displaystyle \Delta ^0(z)=|W|^2\int _\mathbb {R}\frac{d\nu ^0(\varepsilon )}{z-\varepsilon }$ and $\xi$ by (22):

$$\begin{aligned} \int _\mathbb {R}\frac{d\xi (\varepsilon )}{z-\varepsilon } = \frac{1}{z-\Delta ^0(z)}. \end{aligned}$$

This function can be asymptotically expanded to order $k+3$ using (49).

$$\begin{aligned} \begin{array}{rl} \displaystyle \int _{\mathbb {R}} \frac{d\xi (\varepsilon )}{iy-\varepsilon } = &  \dfrac{1}{iy-|W|^2\left( \frac{1}{iy}+\dots +\frac{m_k(\nu ^0)}{(iy)^{k+1}}+o\left( \frac{1}{y^{k+1}}\right) \right) } \\ = &  \displaystyle \frac{1}{iy} \left( 1-\frac{|W|^2}{iy} \left( \frac{1}{iy}+\dots +\frac{m_k(\nu ^0)}{(iy)^{k+1}}+o\left( \frac{1}{(iy)^{k+1}}\right) \right) \right) ^{-1} \\ = &  \displaystyle \frac{1}{iy} + \dots + \frac{m_{k+2}(\xi )}{(iy)^{k+3}} + o\left( \frac{1}{y^{k+3}}\right) . \end{array} \end{aligned}$$

By Theorem 4.1, $\xi$ has finite moments up to order $k+2$, which is even, denoted by $m_0(\xi )=1,\dots ,m_{k+2}(\xi )$. Now let $\mu : = F^{\textrm{IPT}}(\nu ^0)$, so that $\mu \in \mathcal {M}_+(\mathbb {R})$ is given by (25) in the statement of Proposition 3.7. In particular, there exists $C_k \in \mathbb {R}_+$ such that

$$\begin{aligned}  &   \displaystyle \int _\mathbb {R}|\varepsilon |^{k+2}d\mu (\varepsilon ) \\  &   = \displaystyle \int _{\mathbb {R}^3} |\varepsilon _1+\varepsilon _2+\varepsilon _3|^{k+2} \frac{1+e^{-\beta (\varepsilon _1+\varepsilon _2+\varepsilon _3)}}{(1+e^{-\beta \varepsilon _1})(1+e^{-\beta \varepsilon _2})(1+e^{-\beta \varepsilon _3})} d\xi (\varepsilon _1)d\xi (\varepsilon _2)d\xi (\varepsilon _3) \\  &   \le \displaystyle \int _{\mathbb {R}^3} |\varepsilon _1+\varepsilon _2+\varepsilon _3|^{k+2} d\xi (\varepsilon _1)d\xi (\varepsilon _2)d\xi (\varepsilon _3) \\  &   \le \displaystyle \int _{\mathbb {R}^3}C_k\sum _{\underset{i_1+i_2+i_3=k+2}{1\le i_1,i_2,i_3\le k+2}} |\varepsilon _1|^{i_1}|\varepsilon _2|^{i_2}|\varepsilon _3|^{i_3} d\xi (\varepsilon _1)d\xi (\varepsilon _2)d\xi (\varepsilon _3) \\  &   = \displaystyle C_k \sum _{\underset{i_1+i_2+i_3=k+2}{1\le i_1,i_2,i_3\le k+2}} \int _\mathbb {R}|\varepsilon _1|^{i_1} d\xi (\varepsilon _1) \int _\mathbb {R}|\varepsilon _2|^{i_2} d\xi (\varepsilon _2) \int _\mathbb {R}|\varepsilon _3|^{i_3} d\xi (\varepsilon _3) < \infty , \end{aligned}$$

since for $l\le k+2$, $\int _\mathbb {R}|\varepsilon |^ld\xi (\varepsilon ) <\infty$. Thus $\mu$ has finite moments of order less than or equal to $k+2$. Finally, let $\nu :=F^{\textrm{BU}}(\mu )=F^{\textrm{DMFT}}(\nu ^0)$. Equations (36) and (37) read

$$\begin{aligned} \Delta (z)=|W|^2\int _\mathbb {R}\frac{d\nu (\varepsilon )}{z-\varepsilon } = W \left( z - H^0_\perp - \Sigma (z) \right) ^{-1} W^\dagger , \end{aligned}$$

with $\Sigma (z) =U^2\int _\mathbb {R}\frac{d\mu (\varepsilon )}{z-\varepsilon }$. By Theorem 4.1, we can expand $\int _{\mathbb {R}} \frac{d\mu (\varepsilon )}{iy-\varepsilon }$ as *y* goes to $+\infty$ and get

$$\begin{aligned} \Delta (iy)= &   W\left( iy-H^0_\perp -U^2\left( \frac{m_0(\mu )}{iy}+\dots +\frac{m_{k+2}(\mu )}{(iy)^{k+3}}+o\left( \frac{1}{y^{k+3}}\right) \right) \right) ^{-1}W^\dagger \\= &   |W|^2\left( \frac{1}{iy}+\dots +\frac{m_{k+4}(\nu )}{(iy)^{k+5}}\right) +o\left( \frac{1}{y^{k+5}}\right) . \end{aligned}$$

This means that

$$\begin{aligned} \int _{\mathbb {R}} \frac{d\nu (\varepsilon )}{iy-\varepsilon } = \frac{1}{iy}+\dots +\frac{m_{k+4}(\nu )}{(iy)^{k+5}}+o\left( \frac{1}{y^{k+5}}\right) , \end{aligned}$$

which proves, by Theorem 4.1, that $\nu$ has finite moments up to order $k+4$.

## Figures and Tables

**Fig. 1:**
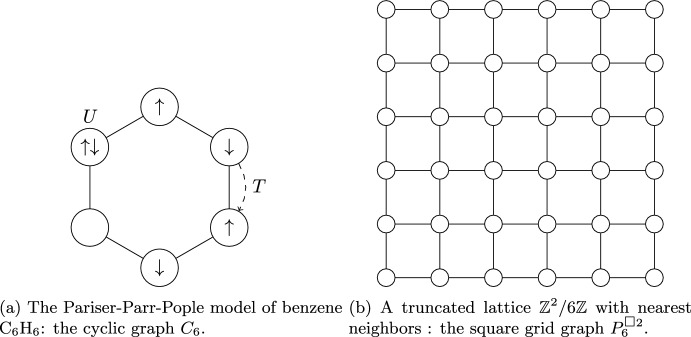
The Hubbard model for different graphs

**Fig. 2:**
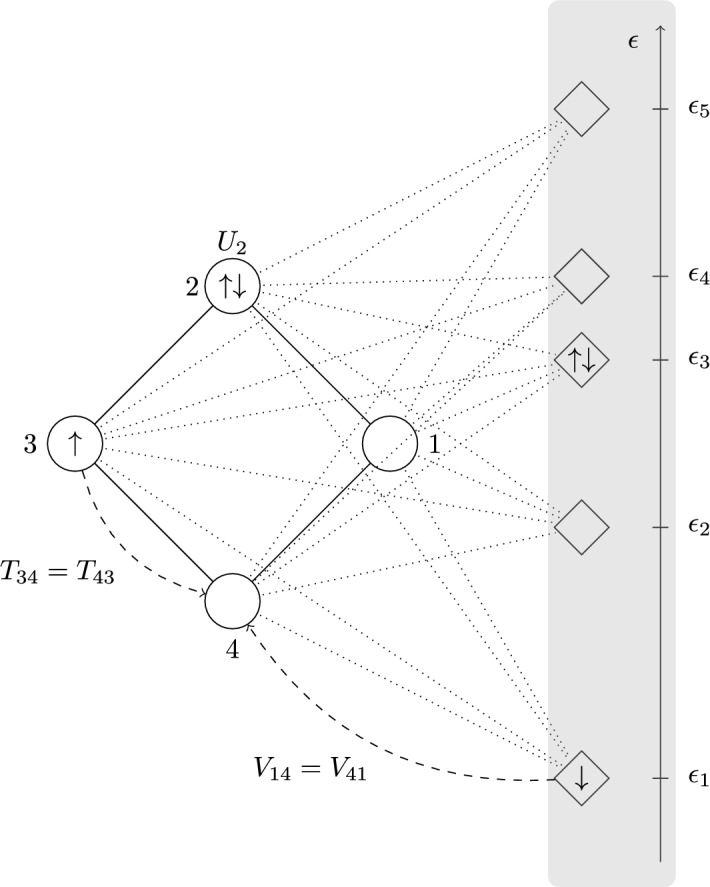
The Anderson Impurity Model of an impurity $(\mathcal {G}_{{H}}=C_4,T_{ },U_{ }$) and a *bath* $(B=5,\epsilon _{ },V_{ })$ in a 6 electrons state

**Fig. 3:**
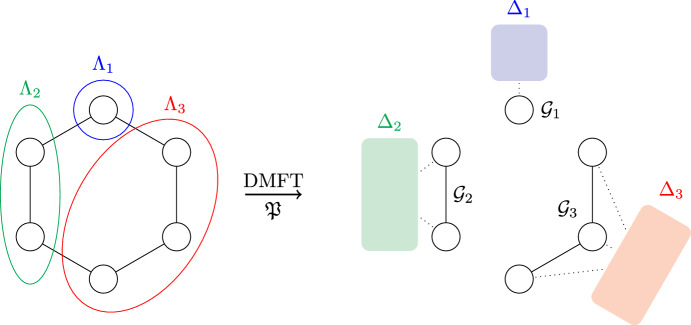
A partition $\mathfrak {P}=\Lambda _1\sqcup \Lambda _2\sqcup \Lambda _3$ of the 6 vertices of the $C_6$ graph, and the 3 induced graphs $\mathcal {G}_{{1}}, \mathcal {G}_{{2}}, \mathcal {G}_{{3}}$: the impurities of the corresponding AIMs are defined using the original Hubbard model, while the bath and the impurity-bath coupling are specified by the DMFT approximation

**Fig. 4:**
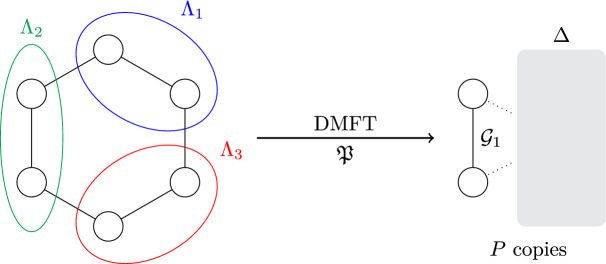
Translation-invariant DMFT formalism

## Data Availability

Data sharing is not applicable to this article as it has no associated data.

## Appendices

In these appendices, we provide pedagogical introductions to two topics which have not been incorporated to the main body of the paper for the sake of brevity.

The first section is devoted to a mathematical derivation of the DMFT equations, which departs from usual presentations in two main respects:

- First, we introduce a mathematically rigorous Green’s functions formalism. To do so, we stick to the setting in which the one-particle Hilbert space is finite dimensional. This choice is motivated by the fact that the statements of the main results and the corresponding proofs are greatly simplified, and the reader is referred to [16] and [39] for an introduction to Green’s function in the infinite dimensional setting. Note also that, instead to considering the (real-frequency) Fourier transform of the Green’s function, as usually done [31, 52], we reinvest the notion of Generalized Fourier Transform, introduced by Titchmarsh [19], which is defined for *any* complex frequency. This enables us to establish a fruitful connection between Green’s functions and (matrix-valued) Pick functions, which forms the basis of our analysis. Adapting our presentation to the infinite dimensional setting would require, in the light of this connection, handling operator-valued Pick functions [29]. Such an approach would involve technical complications that are unnecessary for a pedagogical introduction to DMFT.
- Second, we introduce the Hubbard and Anderson Impurity models using graph theory formalism. This way of defining the models is not new (see e.g. [7] for the fermionic and [4] for the bosonic setting), and allows us to consider various applications. For instance, the notion of “translation invariance" is captured by vertex-transitive graphs, and avoids any spatial embedding. Moreover, we believe that the DMFT formalism is very concisely defined when using notions such as induced subgraphs.

The second section consists of a basic introduction to an Analytic Continuation Problem (ACP) formulated in the set of (matrix-valued) Pick functions. Such a problem appears in the DMFT equations considered in this work because of the deep connection between the formalism of Green’s functions introduced in Sect. A.1 and the Matsubara formalism introduced in Sect. A.5.1. The most important result in this section is Theorem B.9: it shows the one-to-one correspondence between these two formalisms (see Theorem A.17) and more importantly that the IPT solver is well-defined in the framework of a finite-dimensional AIM (leading to Proposition 3.2).

Solving analytic continuation problem for finitely supported measure and then extending the IPT solver by continuity, we circumvent the theoretical and computational technicality related to ACPs.

## DMFT of the Hubbard Model

We provide in this section a mathematical description of the models and quantities of interest involved in Dynamical Mean-Field Theory (DMFT) for the Hubbard model. We first recall the definitions of one-body Green’s functions and the self-energy. We then introduce the Hubbard model and the Anderson Impurity Model (AIM). Next, we derive the DMFT equations and finally present the Iterated Perturbation Theory (IPT) solver, which is the approximate impurity solver considered in this work, together with a derivation of the Matsubara formalism of Green’s functions. We then detail the spin independence of the equations within the paramagnetic framework, and the following simplification of the equations. Finally, in section A.7, we give the proofs of the results stated in this appendix.

The following result, which can also be found in [50], underlines the relation between Pick functions and Green’s function methods. It is central in our mathematical analysis of the IPT-DMFT equations, and justifies the framework of Sect. 2. The mathematical objects involved, namely the Green’s function, the hybridization function and the self-energy are introduced later in this section. Recall that a Pick matrix *M* is a matrix-valued analytic function defined on the upper-half plane, such that for all $z\in \mathbb {C}_+$, the matrix $M(z)-M(z)^\dagger$ is positive semi-definite.

### Proposition A.1

($$-G,-\Delta ,-\Sigma $$ are Pick).

1. Let $\widehat{H}\in \mathcal {S}(\mathcal {F})$ be a Hamiltonian and $G: \mathbb {C}_+\rightarrow \mathcal {L}(\mathcal {H}_{ }) \simeq \mathbb {C}^{n \times n}$ the one-body Green’s function of $\widehat{H}$ in a steady state. Then $-G$ is a Pick matrix.
2. Let $\widehat{H}\in \mathcal {S}(\mathcal {F})$ be a Hamiltonian, with non-interacting component $d\Gamma ({H^0}) \in \mathcal {S}(\mathcal {F})$, $G^0$ the one-body Green’s function of $d\Gamma ({H^0})$, $G$ the one-body Green’s function of $\widehat{H}$ in a steady state of $\widehat{H}$, and $\Sigma : \mathbb {C}_+\rightarrow \mathcal {L}(\mathcal {H}_{ }) \simeq \mathbb {C}^{n \times n}$ the self-energy defined by
  $$\begin{aligned} \Sigma (z):= G^0(z)^{-1} - G(z)^{-1}. \end{aligned}$$
  Then $-\Sigma$ is a Pick matrix.
3. Let $\Delta :\mathbb {C}_+\rightarrow \mathbb {C}^{n \times n}$ be the hybridization function of some Anderson Impurity Model (AIM) with impurity one-particle state space $\mathbb {C}^n$. Then $-\Delta$ is a Pick matrix.

### One-body Green’s Functions and the Self-Energy

One-body Green’s functions are key objects in DMFT. To avoid technicalities, we will define Green’s functions in a finite-dimensional setting and assume that the one-body state space is a finite-dimensional Hilbert space $(\mathcal {H}_{ },\langle \cdot ,\cdot \rangle )$, $\textrm{dim}(\mathcal {H}_{ })=2L \in \mathbb {N}^*$. The associated Fock space

$$\begin{aligned} \mathcal {F}=\bigoplus _{n=0}^{2L}\bigwedge ^n \mathcal {H}_{ }, \end{aligned}$$

where the *n*-particle sector $\displaystyle \bigwedge ^n \mathcal {H}_{ }$ is the anti-symmetrized tensor product of *n* copies of $\mathcal {H}_{ }$, is then of dimension $2^{2L}$. Given a one-particle state $\phi \in \mathcal {H}_{ }$, we denote by $\widehat{a}_{{\phi }}$ (resp. $\widehat{a}_{{\phi }}^\dagger$) the usual annihilation (resp. creation) operator defined on $\mathcal {F}$ (see e.g. [13]), which satisfy the Canonical Anti-commutation Relations (CAR):

$$\begin{aligned} \forall \phi , \phi ' \in \mathcal {H}_{ }, \quad \{ {\widehat{a}_{{\phi }}},{\widehat{a}_{{\phi '}}} \}= \{ {\widehat{a}_{{\phi }}^\dagger },{\widehat{a}_{{\phi }}^\dagger } \}=0,\quad \{ {\widehat{a}_{{\phi }}},{\widehat{a}_{{\phi '}}^\dagger } \}=\langle \phi ,\phi ' \rangle \end{aligned}$$

where $\{ {\widehat{O}},{\widehat{O}'} \}=\widehat{O}\widehat{O}' + \widehat{O}'\widehat{O}$ is the anti-commutator of the two operators $\widehat{O},\widehat{O}' \in \mathcal {L}(\mathcal {F})$.

Steady states A *state* $\Omega$ is a linear form on the set of operators $\mathcal {L}(\mathcal {F})$, which is positive ($\Omega (\widehat{O}^\dagger \widehat{O}) \ge 0$) and normalized (i.e. $\sup \{ \vert {\Omega (\widehat{O})} \vert , \Vert {\widehat{O}} \Vert =1\}=1$). In the finite-dimensional case, any state $\Omega$ can be represented by a unique self-adjoint operator $\widehat{\rho }\in \mathcal {S}(\mathcal {F})$ such that for all $\widehat{O}\in \mathcal {L}(\mathcal {F})$, $\Omega (\widehat{O})=\textrm{Tr}(\widehat{\rho }\widehat{O})$. The operator $\widehat{\rho }$ is positive and satisfies $\textrm{Tr}(\widehat{\rho })=1$. It is called the *density operator* associated with the state $\Omega$. For an isolated quantum system described by a time-independent Hamiltonian $\widehat{H}\in \mathcal {S}(\mathcal {F})$, a *steady state* corresponds to a stationary solution to the quantum Liouville equation

$$\begin{aligned} i \frac{d\widehat{\rho }}{dt}(t) = [\widehat{H},\widehat{\rho }(t)], \end{aligned}$$

where $[\widehat{O},\widehat{O}']=\widehat{O}\widehat{O}' - \widehat{O}'\widehat{O}$ is the commutator of $\widehat{O},\widehat{O}'\in \mathcal {L}(\mathcal {F})$. It follows that a state is a steady state if and only if its density $\widehat{\rho }$ commutes with the Hamiltonian $\widehat{H}$, namely $[{\widehat{H}},{\widehat{\rho }}]=0$. Important examples of steady states are thermal and osmotic equilibrium states known as Gibbs states, as well as ground and excited states of $\widehat{H}$ with a prescribed number of particles (for particle-number conserving Hamiltonians).

*One-body Green’s functions* The *one-body time-ordered Green’s functions* are then defined as follows:

#### Definition A.2

*(One-body time-ordered Green’s function).* Given a Hamiltonian $\widehat{H}\in \mathcal {S}(\mathcal {F})$ and an associated steady state $\Omega$, one defines the $\mathcal {L}(\mathcal {H}_{ })$-valued function $\widetilde{G}: \mathbb {R}\rightarrow \mathcal {L}(\mathcal {H}_{ })$, known as *one-body time-ordered Green’s function*, by requiring that $i\widetilde{G}(t)$ is the operator represented by the sesquilinear form defined, for all $\phi ,\phi ' \in \mathcal {H}_{ }$, by

50 $$\begin{aligned} \langle \phi ,(i\widetilde{G}(t))\phi ' \rangle = 1\!\!1_{{\mathbb {R}_{+}}} (t) \, \Omega (\mathbb {H}({\widehat{a}_{{\phi }}})(t)\widehat{a}_{{\phi '}}^\dagger ) - 1\!\!1_{{\mathbb {R}_{-}^*}} (t) \, \Omega (\widehat{a}_{{\phi '}}^\dagger \mathbb {H}({\widehat{a}_{{\phi }}})(t)), \end{aligned}$$

where for all $\widehat{O}\in \mathcal {L}(\mathcal {F}),\ \mathbb {H}({\widehat{O}}): \mathbb {R}\ni t \mapsto e^{it\widehat{H}} \widehat{O}e^{-it\widehat{H}}$ denotes the *Heisenberg picture* of $\widehat{O}$ and $1\!\!1_{{A}}$ is the characteristic function of the set *A*.

Let us comment on the terminology. First, the term “body” encompasses cases of a “particle” or a “hole”: the first term of the right hand side of (50) can be interpreted as describing the propagation from $t_0=0$ to $t > 0$ of a particle added to the system at $t_0=0$, while the second term can be interpreted as the propagation from $t < 0$ to $t_0=0$ of a hole created at $t<0$. Second, it is “time-ordered”: the r.h.s. of (50) can be rewritten as

$$\begin{aligned} \Omega \left( \mathcal {T}\left( \mathbb {H}({\widehat{a}_{{\phi }}}),\mathbb {H}({\widehat{a}_{{\phi '}}^\dagger })\right) (t,0)\right) \end{aligned}$$

where for all operators-valued functions $\mathbb {R}\ni t \mapsto \widehat{O}(t) \in \mathcal {L}(\mathcal {F}),\mathbb {R}\ni t \mapsto \widehat{O}'(t) \in \mathcal {L}(\mathcal {F})$, the fermionic *time-ordered product* $\mathcal {T}(\widehat{O},\widehat{O}')$ is the operator-valued function $\mathbb {R}^2 \rightarrow \mathcal {L}(\mathcal {F})$ defined as

$$\begin{aligned} \mathcal {T}(\widehat{O},\widehat{O}')(t,t')=\left\{ \begin{array}{ll} \widehat{O}(t) \widehat{O}'(t') \text { if t }\ge \text {t}' \\ -\widehat{O}'(t')\widehat{O}(t) \text { otherwise,} \end{array}\right. \end{aligned}$$

where the minus sign is specific to the fermionic case. Up to a sign, it is the product of the operators applied in the order of increasing time.

The *i* prefactor in the left hand side of (50) is a convention that facilitates the expression of the results to come, especially Proposition A.4.

Finally, note that $\widetilde{G}$ is real-analytic on $(-\infty ,0)\cup (0,+\infty )$ with a $-i\textbf{1}$ jump at $t=0$ due to the CAR.

As $\mathcal {F}$ is finite-dimensional, the Green’s function can be expanded in a joint orthonormal eigenbasis $\mathcal {B}$ of $\widehat{\rho }$ and $\widehat{H}$, leading to the Källén-Lehmann (KL) representation [43, 45]

51 $$\begin{aligned}  &   \forall \phi ,\phi ' \in \mathcal {H}_{ }, \quad \langle \phi ,i\widetilde{G}(t)\phi ' \rangle =\nonumber \\  &   \sum _{\psi ,\psi ' \in \mathcal {B}} e^{it(E_{\psi }-E_{\psi '})}\langle \psi ,\widehat{a}_{{\phi }}\psi ' \rangle \langle \psi ',\widehat{a}_{{\phi '}}^\dagger \psi \rangle \left( \rho _\psi 1\!\!1_{{\mathbb {R}_+}}(t) - \rho _{\psi '} 1\!\!1_{{\mathbb {R}_-^*}}(t)\right) , \end{aligned}$$

where $\forall \psi \in \mathcal {B}, \widehat{H}\psi = E_{\psi } \psi$ (with $E_{\psi } \in \mathbb {R}$) and $\widehat{\rho }\psi =\rho _{\psi } \psi$ (with $\rho _{\psi } \in \mathbb {R}_+$, $\sum _{\psi \in \mathcal {B}} \rho _{\psi }=1$).

Other types of Green’s functions are encountered in the physics literature, notably retarded and advanced Green’s functions. These objects encode the same information on the spectral properties of $\widehat{H}$ as the time-ordered Green’s function, but this information is stored in a different way. A suitable way to highlight this information is to consider specific holomorphic extensions to the complex plane of the time-Fourier transform of these Green’s functions [16, 52]. In the case of the time-ordered one-body Green’s function, the suitable holomorphic extension is provided by the generalized Fourier transform introduced by Titchmarsh [19].

#### Definition A.3

*(Generalized Fourier Transform (GFT)).* The Generalized Fourier Transform (GFT) of the one-body time-ordered Green’s function $\widetilde{G}$ is the analytic function on the upper half-plane $G:\mathbb {C}_+\rightarrow \mathcal {L}(\mathcal {H}_{ })$, also called (one-body) Green’s function, defined by

52 $$\begin{aligned} \forall z \in \mathbb {C}_+, G(z)&= G_+(z)+G_{-}(\overline{z})^\dagger \end{aligned}$$

with

53 $$\begin{aligned}&\forall z \in \mathbb {C}_+:=\{ z \in \mathbb {C}\ | \ \Im (z)>0 \}, \quad G_+(z)&=\int _{\mathbb {R}_+}e^{izt} \widetilde{G}(t) dt, \end{aligned}$$

54 $$\begin{aligned}&\forall z \in \mathbb {C}_-:=\{ z \in \mathbb {C}\ | \ \Im (z) < 0 \}, \quad G_-(z)&=\int _{\mathbb {R}_-}e^{izt} \widetilde{G}(t) dt. \end{aligned}$$

Note that the Green’s function $G$ can be extended to $\mathbb {C}\setminus \mathbb {R}$ by reflection, namely by setting

55 $$\begin{aligned} \forall z \in \mathbb {C}_-, \quad G(z)=G(\bar{z})^\dagger . \end{aligned}$$

By construction, $G(z)$ is analytic on $\mathbb {C}_+$. In addition, it follows from the KL representation (51) that

56 $$\begin{aligned} \forall z \in \mathbb {C}_+, \quad \forall \phi ,\phi ' \in \mathcal {H}_{ }, \quad \langle \phi ,G(z)\phi ' \rangle =\sum _{\psi ,\psi ' \in \mathcal {B}} \frac{\rho _{\psi }+\rho _{\psi '}}{z + (E_{\psi }-E_{\psi '})} \langle \psi ,\widehat{a}_{{\phi }}\psi ' \rangle \langle \psi ',\widehat{a}_{{\phi '}}^\dagger \psi \rangle . \nonumber \\ \end{aligned}$$

An important observation is that

57 $$\begin{aligned}  &   \forall z \in \mathbb {C}_+, \quad \forall \phi \in \mathcal {H}_{ }\setminus \{0\}, \quad \Im \left( \langle \phi ,G(z)\phi \rangle \right) \nonumber \\  &   =- \Im (z) \sum _{\psi ,\psi ' \in \mathcal {B}} \frac{\rho _{\psi '}+\rho _\psi }{|z+(E_{\psi }-E_{\psi '})|^2} |\langle \psi ,\widehat{a}_{{\phi }}\psi ' \rangle |^2 <0, \end{aligned}$$

which shows in particular that $G(z)$ is invertible for all $z \in \mathbb {C}_+$.

Up to now, we have not specified the Hamiltonian $\widehat{H}$; in the sequel, we will assume that it is of the form

58 $$\begin{aligned} \widehat{H}=d\Gamma ({H^0}) + \widehat{H}^I, \quad H^0\in \mathcal {S}(\mathcal {H}_{ }), \quad \widehat{H}^I\in \mathcal {S}(\mathcal {F}) \end{aligned}$$

where $d\Gamma ({H^0})$ is the second quantization of the one-particle Hamiltonian $H^0\in \mathcal {S}(\mathcal {H}_{ })$ (see e.g. [13]) and $\widehat{H}^I\in \mathcal {S}(\mathcal {F})$ some interaction Hamiltonian. We say that $\widehat{H}$ is *non-interacting* if $\widehat{H}^I=0$.

Depending on the formalism of interest, one can consider the grand canonical Hamiltonian $\widehat{H}'=\widehat{H}-\epsilon _F\widehat{N}$ without loss of generality.

The following is an essential property of the Green’s function, to which it owes its name: the Green’s function of a non-interacting system in a steady state is the resolvent of the one-particle Hamiltonian.

#### Proposition A.4

(Non-interacting Green’s function). Let $H^0\in \mathcal {S}(\mathcal {H}_{ })$ be a one-particle Hamiltonian and $\Omega$ a steady state of the non-interacting Hamiltonian $d\Gamma ({H^0})$. The Green’s function $G^0$ of $d\Gamma ({H^0})$ associated with $\Omega$ is the resolvent of $H^0$:

59 $$\begin{aligned} \forall z \in \mathbb {C}_+, \quad G^0(z)=(z-H^0)^{-1}. \end{aligned}$$

In particular, $G^0$ is independent of $\Omega$.

The proof of this classical result is recalled in Sect. A.7.1. It is a consequence of the fact that the one-body Green’s function $\widetilde{G}^0$ of the non-interacting Hamiltonian $d\Gamma ({H^0})$ is solution to the equation

60 $$\begin{aligned} \left( i\frac{d}{dt} -H^0\right) \widetilde{G}= \delta \textbf{1}\quad \text { in } \mathcal {D}'(\mathbb {R};\mathcal {L}(\mathcal {H}_{ })) \end{aligned}$$

so that the time-ordered Green’s function $\widetilde{G}^0$ is indeed a Green’s function of the linear differential operator $i\frac{d}{dt} - H^0$. The various avatars of the Green’s function (retarded/advanced) also satisfy this equation in $\mathcal {D}'(\mathbb {R}^*;\mathcal {L}(\mathcal {H}_{ }))$, but with different asymptotic conditions at infinity and jumps at $t=0$. The properties (59)–(60) hold *only* for non-interacting Hamiltonians.

Self-energy. For interacting systems, the general relation between the Hamiltonian and the Green’s function is more involved: the discrepancy with the non-interacting case is characterized by the *self-energy*.

#### Definition A.5

*(Self-energy).* The *self-energy* $\Sigma :\mathbb {C}_+\rightarrow \mathcal {L}(\mathcal {H}_{ })$ of an Hamiltonian $\widehat{H}$ of the form (58) associated with a steady state $\Omega$ of $\widehat{H}$ is defined by

61 $$\begin{aligned} \forall z \in \mathbb {C}_+, \quad \Sigma (z)=G^0(z)^{-1} - G(z)^{-1}, \end{aligned}$$

where the *non-interacting Green’s function* $G^0$ is the Green’s function of any steady state of $d\Gamma ({H^0})$.

Recall that $G^0(z)^{-1} = z-H^0$ (59) and that *G*(*z*) is invertible in view of (57). Let us emphasize that the definition of the self-energy only depends on $\widehat{H}$, $H^0$, and the considered steady state $\Omega$ of $\widehat{H}$, since $G^0$ is independent of the steady state associated with $d\Gamma ({H^0})$.

Using again (59) and the definition of the self-energy, the Green’s function *G* can be rewritten as

$$\begin{aligned} \forall z \in \mathbb {C}_+, \quad G(z)=\left( z-(H^0+\Sigma (z))\right) ^{-1} \end{aligned}$$

so that, for a given complex frequency *z*, $H^0+ \Sigma (z)$ can be considered as an *effective one-particle Hamiltonian* (compare with (59)): the self-energy is thus the extra term to be added to $H^0$ to obtain a representation of the interacting system of particles in terms of a system of non-interacting ones. The frequency dependence of $\Sigma$ comes from the fact that particles do interact in the original system.

#### Remark A.6

In condensed matter physics, the Nevanlinna-Riesz measure of $-G$ is the so-called spectral function [52].

### Hubbard Model

Originally introduced in quantum chemistry [59, 61], the Hubbard model [33, 36, 38] is an idealized model that minimally describes *interacting electrons* in a molecule or a crystalline material. From the mathematical physics perspective, it is a prototypical example of short-range fermionic lattice system, and its mathematical study has been pioneered soon after its introduction in [47, 48], triggering an extensive study of its ground-states properties [46]. More recently, the discovery of cuprate-based high-temperature superconductors [9, 78], exhibiting a *layered structure*, shed new light on the *square lattice* Hubbard model, for which much remains to be discovered [46]. Since then, *approximation methods* have been derived for this model such as *generalized Hartree-Fock* [7], and Dynamical Mean-Field Theory [27].

In this article, we restrict ourselves to *finite-dimensional* Hubbard models, i.e. to Hubbard models defined on *finite graphs*. The reason for this is threefold. First, we stick to the case of finite-dimensional state spaces, for which all the objects involved in the mathematical formulation of DMFT are well-defined. Second, *graph theory* provides a suitable formalism to describe on the same footing different physical settings, ranging from molecular systems to truncated lattices, and with arbitrary hopping parameters (nearest neighbours, next nearest neighbors,...). Third, this is precisely the language in which DMFT can be formulated concisely, as described in Sect. A.4.

Consider now a finite undirected graph $\mathcal {G}_{{H}}=(\Lambda ,E)$ that describes the “sites” $\Lambda$ hosting electrons, as depicted in Fig. 1. The state space of a site is the vector space spanned by the orthonormal vectors $\vert \emptyset \rangle$ (empty site), $\vert \! \uparrow \rangle$ (site occupied by one spin-up electron), $\vert \! \downarrow \rangle$ (site occupied by one spin-down electron), and $\vert \! \uparrow \downarrow \rangle$ (doubly-occupied site). Note that $\vert \! \uparrow \downarrow \rangle$ is a shorthand notation for the two-electron singlet state $2^{-1/2}(\vert \! \uparrow \rangle \otimes \vert \! \downarrow \rangle - \vert \! \downarrow \rangle \otimes \vert \! \uparrow \rangle )$. In chemistry language, to each site is associated a single orbital (and thus two spin-orbitals); in physics, this setting is referred to as the *one-band* Hubbard model. Since the sites are distinguishable, the state space of the full system is the tensor product of the state space of each site. It is therefore of dimension $4^{\vert {\Lambda }\vert }=2^{2L}$ where $L=\vert {\Lambda }\vert$ is the number of sites.

Let us now specify the Hamiltonian. In the Hubbard model, electrons can jump from one site to any other neighboring site. This models the tunnel effect, whose intensity is described by the *hopping matrix* $T_{ }$, as for tight-binding Hamiltonians. Electrons repel each other due to (screened) Coulomb interaction. The simplicity of the Hubbard model lies in the *range* of this interaction, which is the shortest possible: it is only effective for two electrons occupying the same site, and if the site *i* is doubly occupied, the energy of the system is increased by an *on-site repulsion* $U_{{i}}$.

More precisely, a finite-dimensional Hubbard model can be defined as follow.

#### Definition A.7

*(Hubbard model of a finite graph).* Given a finite graph $\mathcal {G}_{{H}}=(\Lambda ,E)$, a hopping matrix $T_{ }:E\rightarrow \mathbb {R}$, and an on-site repulsion $U_{ }: \Lambda \rightarrow \mathbb {R}$, the Hubbard Fock Space $\mathcal {F}_{H}$ is defined as

$$\begin{aligned} \mathcal {F}_{H}=\bigotimes _{i \in \Lambda } \mathcal {F}_1, \quad \mathcal {F}_1=\textrm{Span}(\vert \emptyset \rangle ,\vert \! \uparrow \rangle ,\vert \! \downarrow \rangle ,\vert \! \uparrow \downarrow \rangle ) \end{aligned}$$

and the Hubbard Hamiltonian $\widehat{H}_H\in \mathcal {S}(\mathcal {F}_{H})$ as

$$\begin{aligned} \widehat{H}_H=\sum _{\{i,j\} \in E, \sigma \in \{\uparrow ,\downarrow \}}T_{{i,j}}\left( \widehat{a}_{{i,\sigma }}^\dagger \widehat{a}_{{j,\sigma }}+ \widehat{a}_{{j,\sigma }}^\dagger \widehat{a}_{{i,\sigma }}\right) + \sum _{i \in \Lambda } U_{{i}}\widehat{n}_{{i,\uparrow }} \widehat{n}_{{i,\downarrow }} \end{aligned}$$

where the usual annihilation and creation operators $\widehat{a}_{{i,\sigma }}$ and $\widehat{a}_{{i,\sigma }}^\dagger$ of an electron on site *j* with spin $\sigma$ satisfy the CAR

62 $$\begin{aligned} \forall i,j \in \Lambda , \quad \sigma \in \{\uparrow ,\downarrow \}, \quad \{ {\widehat{a}_{{i,\sigma }}},{\widehat{a}_{{j,\sigma '}}} \}= \{ {\widehat{a}_{{i,\sigma }}^\dagger },{\widehat{a}_{{j,\sigma '}}^\dagger } \}=0,\quad \{ {\widehat{a}_{{i,\sigma }}},{\widehat{a}_{{j,\sigma '}}^\dagger } \}=\delta _{i,j}\delta _{\sigma ,\sigma '}, \nonumber \\ \end{aligned}$$

and the site number operators ${\widehat{n}}_{i,\sigma }$ are defined by ${\widehat{n}}_{i,\sigma }=\widehat{a}_{{i,\sigma }}^\dagger \widehat{a}_{{i,\sigma }}$.

In this paper, we do not consider external magnetic field, so that we can assume without loss of generality that the hopping matrix $T_{ }$ is real-valued [46]. For standard Coulomb interaction, the on-site repulsion $U_{ }$ is positive, but we do not need to make this assumption here. Let us finally mention that this model falls within the class of quantum lattice systems (see [13, 14, 77] for a discussion about the mathematical and physical properties of these systems).

#### Remark A.8

The Hubbard Hamiltonian $\widehat{H}_H$ is particle-number and spin conserving, i.e. it commutes with the number operator $\widehat{N}_H=\sum _{i \in \Lambda } \left( \widehat{n}_{{i,\uparrow }} + \widehat{n}_{{i,\downarrow }}\right)$ and the spin operators. In particular, it commutes with the *z*-component $\widehat{S}_{H}^z=\frac{1}{2}\sum _{i \in \Lambda } \left( \widehat{n}_{{i,\uparrow }}-\widehat{n}_{{i,\downarrow }}\right)$ of the spin operator. This property will be used later, in Appendix A.6, to make the IPT-DMFT model spin-independent.

### Anderson Impurity Model (AIM)

Impurity models Impurity models are models in which an “impurity” is coupled to a “bath” in such a way that particles in the bath do not interact, and the coupling Hamiltonian between the impurity and the bath only involves one-body terms. Otherwise stated, the one-particle state space and the Fock space of the total system can be decomposed as

63 $$\begin{aligned} \mathcal {H}_{\textrm{IM}} = {\mathcal {H}}_\textrm{imp} \oplus {\mathcal {H}}_\textrm{bath} \quad \text{ and } \quad \mathcal {F}_\textrm{IM} = \mathcal {F}_\textrm{imp} \otimes \mathcal {F}_\textrm{bath}, \end{aligned}$$

and its Hamiltonian as

64 $$\begin{aligned} \widehat{H}_\textrm{IM} = \widehat{H}_\textrm{imp} \otimes 1_\textrm{bath} + 1_\textrm{imp} \otimes \widehat{H}_\textrm{bath} + \widehat{H}_\textrm{coupling}, \end{aligned}$$

with

65 $$\begin{aligned} \widehat{H}_\textrm{imp} = d\Gamma (H^0_\textrm{imp}) + \widehat{H}_\textrm{imp}^\textrm{I}, \quad \widehat{H}_\textrm{bath}= d\Gamma (H^0_\textrm{bath}), \quad \widehat{H}_\textrm{coupling}= d\Gamma (H^0_\textrm{coupling}), \end{aligned}$$

and $H^0_\textrm{coupling}$ can be decomposed according to (63) as

$$\begin{aligned} H^0_\textrm{coupling} = \left( \begin{array}{cc} 0 &  V \\ V^\dagger &  0 \end{array} \right) . \end{aligned}$$

Reshuffling the terms, we also have

66 $$\begin{aligned} \widehat{H}_\textrm{IM} = d\Gamma (H^0_\textrm{IM}) + \widehat{H}_\textrm{imp}^\textrm{I} \otimes 1_\textrm{bath}, \quad \text{ with } \quad H^0_\textrm{IM} = \left( \begin{array}{cc} H^0_\textrm{imp} &  V \\ V^\dagger &  H^0_\textrm{bath} \end{array} \right) . \end{aligned}$$

As will be seen below, a key step of the DMFT iteration loop is to compute the restriction of the Green’s function $G_\textrm{IM}$ of an impurity model to the impurity space $\mathcal {H}_{{\textrm{imp}}}$. This is in general a difficult task. On the other hand, computing the restriction of the *non-interacting* Green’s function can be done straightforwardly using a Schur complement technique. This leads to the concept of hybridization function, which, as announced in the introduction, is of paramount importance in the mathematical formulation of DMFT.

#### Definition A.9

*(Hybridization function* $\Delta$ *of an impurity model).* Given an impurity model of the form (63)–(66), its *hybridization function* $\Delta :\mathbb {C}_+\rightarrow \mathcal {L}(\mathcal {H}_{{\textrm{imp}}})$ is defined by

67 $$\begin{aligned} \forall z \in \mathbb {C}_+, \quad \Delta (z)=V \left( z-H^0_{\textrm{bath}}\right) ^{-1}V^\dagger . \end{aligned}$$

Using this definition and Proposition A.4, the non-interacting Green’s function of the impurity model is then given in the decomposition (63) by

$$\begin{aligned} G^0(z) = (z-H^0_\textrm{IM})^{-1} = \left( \begin{array}{cc} (z-H^0_\textrm{imp}-\Delta (z))^{-1} &  * \\ * &  * \end{array} \right) . \end{aligned}$$

The hybridization function $\Delta$ thus plays a role analogous to the self-energy $\Sigma$. The equation

$$\begin{aligned} \left( G^0(z)\right) _\textrm{imp}=\left( z-(H^0_{\textrm{imp}}+\Delta (z))\right) ^{-1} \end{aligned}$$

means that $H^0_{\textrm{imp}}+\Delta (z)$ can be considered as an *effective one-particle impurity Hamiltonian*: the hybridization function is the extra term to be added to $H^0_{\textrm{imp}}$ so that, in the non-interacting case, a particle in the whole system can be regarded as a particle localized on the impurity. In the time domain, the hybridization function describes the phenomenon of a particle localized on the impurity, jumping into the bath, and jumping back to the impurity, hence the name *hybridization*.

The most important result in this section is the following.

#### Theorem A.10

($\Sigma$ sparsity pattern of an impurity model). Given an impurity model of the form (63)–(66), the self-energy $\Sigma _\textrm{IM}: \mathbb {C}_+\rightarrow \mathcal {H}_{\textrm{IM}}$ associated with any steady state of $\widehat{H}_\textrm{IM}$ reads in the decomposition (63)

68 $$\begin{aligned} \forall z \in \mathbb {C}_+, \quad \Sigma _\textrm{IM}(z)=\left( \begin{array}{ll} \Sigma _\textrm{imp}(z) & 0 \\ 0 & 0 \end{array} \right) . \end{aligned}$$

In practical DMFT computations and as mentioned already in [23], the self-energy $\Sigma$ of an impurity problem depends solely on the hybridization function $\Delta$ and on the impurity Hamiltonian defined by $H^0_{\textrm{imp}}$ and $\widehat{H}^I$, via a quantum action defined by path integrals [32, eq. 9]. In this article, we focus on the IPT approximation (see section A.5), which makes this dependence almost explicit, and postpone the study of the exact impurity solver in a more general framework to future works.

Anderson Impurity Model The original Anderson impurity model (AIM) [5] is a specific impurity model in which the impurity consists of a single-site Hubbard model. It was introduced by Anderson back in 1961 to explain the low-temperature behavior of the conductivity of metallic alloys with dilute magnetic impurities, later called the *Kondo effect* [41, 65]. The AIM was then extended to multiple-site Hubbard impurities. Figure 2 provides a graphical illustration of an AIM model with a 4-site Hubbard impurity and 5 bath orbitals ($\textrm{dim}({\mathcal {H}}_\textrm{imp})=8$, $\textrm{dim}({\mathcal {H}}_\textrm{bath})=10$). Without loss of generality, we can indeed identify the bath space ${\mathcal {H}}_\textrm{bath}$ with $\mathbb {C}^{2B}$ and assume that $H^0_\textrm{bath}=\textrm{diag}(\epsilon _1, \epsilon _1, \cdots , \epsilon _B, \epsilon _B)$. In this picture, the canonical basis of $\mathbb {C}^{2B}$ corresponds to an orthonormal basis of bath spin-orbitals with energies $\epsilon _1, \epsilon _1, \dots , \epsilon _B, \epsilon _B$. The matrix $V_{ }$ models jumps from the bath to the impurity, while the matrix $V_{ }^\dagger$ models jumps from the impurity to the bath. The coupling terms $V_{i,j}$ are also assumed to be real-valued due to the absence of magnetic field.

In the seminal paper [5], the *electronic bath* was thought as the set of *conducting electrons*, for instance described by a tight-binding model on a (truncated) lattice.

#### Remark A.11

As for the Hubbard model, the z-component of the total spin operator $\widehat{S}_{AI}^z=\widehat{S}_{H}^z+\sum _{k=1}^B\widehat{n}_{{k,\uparrow }} - \widehat{n}_{{k,\downarrow }}$, the total number operator $\widehat{N}_{AI}=\widehat{N}_H+\sum _{k=1}^B\widehat{n}_{{k,\uparrow }} + \widehat{n}_{{k,\downarrow }}$ and the Anderson Impurity Hamiltonian ${\widehat{H}}_\textrm{AIM}$ pairwise commute. This property will also be used to make the IPT-DMFT model spin-independent.

### Dynamical Mean-Field Theory (DMFT)

Consider a Hubbard model defined by $(\mathcal {G}_{{H}},T_{ },U_{ })$ in one of its steady states $\Omega$. The purpose of DMFT is to provide an *approximation* of the corresponding Green’s function $G$, and more precisely of some blocks of this Green’s function.

To do so, DMFT uses a self-consistent mapping between the Hubbard model $(\mathcal {G}_{{H}},T_{ },U_{ })$ and a collection of Anderson Impurity Models associated with a partition $\mathfrak {P}$ of the vertices of the Hubbard graph $\mathcal {G}_{{H}}$. The process, illustrated in Fig. 3, is the following.

1. Choose a partition $\mathfrak {P}=\{\Lambda _p, p \in [\![ {1},{P}]\!] \}$ of the *L* sites of the Hubbard model into *P* impurities $\Lambda _{1},\cdots \Lambda _{P}$,, $\sqcup _{p=1}^{P}\Lambda _{p}=\Lambda$, and consider for all $p \in [\![ {1},{P}]\!]$ the *induced subgraphs* $\mathcal {G}_{{p}}=(\Lambda _{p},E_{p})$ with $E_{p}=\{\{i,j\} \in E; \ i,j \in \Lambda _{p}\}$. This partition leads to the canonical orthogonal decomposition
  69 $$\begin{aligned} \mathcal {H}_{{H}}=\bigoplus _{p=1}^P\mathcal {H}_{{p}}, \quad \textrm{dim}(\mathcal {H}_{{p}})=2|\Lambda _p|=2L_p, \end{aligned}$$
  from which follows the decomposition of the Fock space
  70 $$\begin{aligned} \mathcal {F}_H = \bigotimes _{p=1}^P\mathcal {F}_p, \quad \dim \mathcal {F}_p = 4^{L_p}. \end{aligned}$$
  The decomposition (69) of the one-particle state space of the original Hubbard model gives rise to block-operator representations of the exact Green’s function and self-energy (for the state $\Omega$) of the original Hubbard model
  $$\begin{aligned} G(z)=\left( \begin{array}{llll} G_1(z) & *& \cdots & *\\ *&  G_2(z)& \cdots & * \\ \vdots & \vdots & \ddots & \vdots \\ *& *& \cdots &  G_P(z) \end{array}\right) , \qquad \Sigma (z)=\left( \begin{array}{llll} \Sigma _1(z) & *& \cdots & *\\ *&  \Sigma _2(z)& \cdots & * \\ \vdots & \vdots & \ddots & \vdots \\ *& *& \cdots &  \Sigma _P(z) \end{array}\right) ; \end{aligned}$$
2. The decomposition (69) also leads to a block-operator representation of the one-body Hamiltonian corresponding to the non-interacting part of the Hubbard Hamiltonian
  $$\begin{aligned} H^0_\textrm{H}=\left( \begin{array}{llll} H^0_{1} & *& \cdots & *\\ *&  H^0_{2} & \cdots & * \\ \vdots & \vdots & \ddots & \vdots \\ *& *& \cdots &  H^0_P\end{array}\right) , \end{aligned}$$
  where the diagonal block $H^0_p$ is constructed from the *induced hopping matrices* $T_{{p}}=T_{{|E_{p}}}$, $1 \le p \le P$. Due to the locality of the interactions in the Hubbard model, the interaction term is “block-diagonal” in the decomposition  (70). It is represented by the *induced on-site repulsion* $U_{{p}}=U_{{|\Lambda _p}}$, and it reads $\widehat{H}^I= \oplus _{p=1}^P\widehat{H}^I_p$, with $\widehat{H}^I_p = \sum _{i\in \Lambda _p}U_i\widehat{n}_{{i,\uparrow }}\widehat{n}_{{i,\downarrow }}$;
3. The AIM associated with the *p*-th impurity is defined by (i) the impurity state space $\mathcal {H}_{{\textrm{imp},p}}:=\mathcal {H}_{{p}}$, (ii) the impurity Hamiltonian defined by the induced Hubbard model $(\mathcal {G}_{{p}},T_{{p}},U_{{p}})$, (iii) some bath state space $\mathcal {H}_{{\textrm{bath},p}}$, bath one-body Hamiltonian $H^0_{\textrm{bath},p}$ and coupling one-body Hamiltonian $$H^0_{\textrm{coupling},p}= \left( \begin{array}{cc} 0 &  V_p^\dagger \\ V_p &  0 \end{array} \right) $$ to be specified. From each of these *P* AIMs, one can compute the Green’s functions and self-energies
  $$\begin{aligned} G_{\textrm{AI},p}(z) = \left( \begin{array}{cc} G_{\textrm{imp},p}(z) &  * \\ * &  * \end{array} \right) , \qquad \Sigma _{\textrm{AI},p}(z) = \left( \begin{array}{cc} \Sigma _{\textrm{imp},p}(z) &  0 \\ 0 &  0 \end{array} \right) , \end{aligned}$$
  for AIM steady states $\Omega _p$ of the same nature as $\Omega$ (see Remark A.12 below for a comment on this important point).

DMFT aims at computing approximations of the diagonal-blocks $G_1(z), \cdots , G_P(z)$ of the exact Green’s function *G*(*z*). Ideally, the baths should be ajusted in such a way that $G_{\textrm{imp},p} =G_p$, but of course this is not possible since the functions $G_p$ are unknown. To make DMFT a practical tool, the idea is to introduce approximate Green’s functions and self-energies of the form

71 $$\begin{aligned} G_\textrm{DMFT}(z)&=\left( \begin{array}{llll} G_{\textrm{DMFT},1}(z) & *& \cdots & *\\ *&  G_{\textrm{DMFT},2}(z) & \cdots & * \\ \vdots & \vdots & \ddots & \vdots \\ *& *& \cdots &  G_{\textrm{DMFT},P}(z) \end{array}\right) , \end{aligned}$$

72 $$\begin{aligned} \Sigma _\textrm{DMFT}(z)&=\left( \begin{array}{llll} \Sigma _{\textrm{DMFT},1}(z) &  0 & \cdots &  0\\ 0&  \Sigma _{\textrm{DMFT},1}(z)& \cdots & 0 \\ \vdots & \vdots & \ddots & \vdots \\ 0& 0& \cdots &  \Sigma _{\textrm{DMFT},P}(z) \end{array}\right) , \end{aligned}$$

related by

$$\begin{aligned} G_{\textrm{DMFT}}(z)&= ((G^0_\textrm{DMFT})^{-1}(z)- \Sigma _{\textrm{DMFT}}(z))^{-1},\\ G^0_{\textrm{DMFT}}(z)&= \left( z-H^0_{\textrm{H}} \right) ^{-1}, \end{aligned}$$

and to choose the baths in such a way that

73 $$\begin{aligned} \forall 1 \le p \le P, \quad&G_{\textrm{DMFT},p}(z)=G_{\textrm{imp},p}(z), \end{aligned}$$

74 $$\begin{aligned}&\Sigma _{\textrm{DMFT},p}(z)=\Sigma _{\textrm{imp},p}(z). \end{aligned}$$

Of course, it is not clear whether a collection of baths that satisfies the above conditions exists, and this article partially answers this question.

Note that the DMFT Green’s function $G_\textrm{DMFT}(z)$ is *not*, a priori, the Green’s function of some interacting quantum many-body problem which could be defined as in Sect. A.1. Instead, it is defined from an approximate self-energy $\Sigma _\textrm{DMFT}(z)$. Equations (72) and (74) indicate that, in the DMFT approximation, each impurity interacts with its neighborhood as if the former were an impurity and the latter a bath, in accordance with Theorem A.10, hence the name of *mean-field*. This mean-field is *dynamical* because it is frequency dependent, in contrast with static mean-field theory such as in Hartree-Fock or Density-Matrix Embedding Theory (DMET).

#### Remark A.12

In the above sketch of the DMFT framework, we did not specify the states $(\Omega _p)_{p \in [\![ {1},{P}]\!]}$ of the associated AIMs from which the self-energies $\left( \Sigma _{\textrm{imp},p}\right) _{p\in [\![ {1},{P}]\!]}$ are computed. In [27], it is implicitly stated that, for a Hubbard model in the *Gibbs* state $\Omega _{\textrm{H},\beta ,\epsilon _F}$ as defined below in Sect. A.5.1, the AIM’s steady states $\Omega _p$ are defined to be *Gibbs* states as well $\Omega _p=\Omega _{\textrm{AIM},\beta ,\epsilon _F'}$, with the same temperature as that of the *Gibbs* state of the whole system, but with *a priori* different chemical potential $\epsilon _F'$. The latter is to be chosen to satisfy appropriate *filling* conditions. We will address this question in a future work and simply assume here that the $\epsilon _F'=\epsilon _F$. Moreover, when working with the IPT solver, the chemical potential is fixed by the on-site interaction, as detailed in Sect. A.5.

Our analysis is based on a reformulation of conditions (73)–(74) using the hybridization functions $(\Delta _p(z))_{1 \le p \le P}$ of the *P* impurity problems as the main variables. As mentionned previously, knowing $\Delta _p$ as well as $T_p$, $U_p$ and $\Omega _p$, suffices in principle to compute $\Sigma _{\textrm{imp},p}$. In practice, this is done by using an approximate impurity solver. A particular example of such a solver will be presented in Sect. A.5. Conversely, knowing $(\Sigma _{\textrm{imp},p})_{1 \le p \le P}$ and *T* suffices to characterize the unique set $(\Delta _p(z))_{1 \le p \le P}$ for which (73)–(74) hold true. Indeed, by denoting

$$\begin{aligned}  &   \mathcal {H}_{{\overline{p}}}:= \bigoplus _{p \ne q=1}^P \mathcal {H}_{{q}}, \\  &   \Sigma _{\textrm{DMFT},\overline{p}}(z) =\left( \begin{array}{lllllll} \Sigma _{\textrm{DMFT},1}(z) &  \cdots & 0 & \cdots &  0\\ \vdots &  \ddots &  \vdots &  &  & \\ 0 &  \cdots &  \Sigma _{\textrm{DMFT},p-1}(z) &  0 &  &  & \\ 0 &  \cdots &  0 &  \Sigma _{\textrm{DMFT},p+1}(z) & \cdots &  0\\ \vdots & \ddots & \vdots & \vdots \\ 0& \cdots &  0 &  0 &  \cdots &  \Sigma _{\textrm{DMFT},P}(z)\\ \end{array}\right) , \end{aligned}$$

and using the Schur complement formula, we have on the one hand

75 $$\begin{aligned} G_{\textrm{DMFT},p}(z)&=\left( \left( z-(H^0_\textrm{H}+\Sigma _{\textrm{DMFT}}(z))\right) ^{-1}\right) _p \nonumber \\&= \left( z- \left( H^0_p+\Sigma _{\textrm{DMFT},p}(z)\right) - W_p \left( z- \left( H^0_{\overline{p}} + \Sigma _{\textrm{DMFT},\overline{p}}(z)\right) \right) ^{-1} W_p^\dagger \right) ^{-1}, \end{aligned}$$

where

76 $$\begin{aligned} \begin{pmatrix} H^0_p &  W_{{p}}\\ W_{{p}}^\dagger &  H^0_{\bar{p}} \end{pmatrix} \text { and } \left( \begin{array}{cc} z-(H^0_p+\Sigma _\textrm{DFMT,p}(z)) &  -W_p \\ -W_p^\dagger &  z-(H^0_{\overline{p}}+\Sigma _\mathrm{DFMT,\overline{p}}(z)) \end{array} \right) \end{aligned}$$

are the block-representations of $H^0_\textrm{H}$ and $(z-(H^0_\textrm{H}+\Sigma _\textrm{DMFT}(z)))$, respectively, in the decomposition $\mathcal {H}_{ }=\mathcal {H}_{{p}} \oplus \mathcal {H}_{{\overline{p}}}$. Note that $H^0_p \in \mathcal {L}(\mathcal {H}_{{p}})$, $W_p \in \mathcal {L}\left( \mathcal {H}_{{\overline{p}}};\mathcal {H}_{{p}} \right)$, and $H^0_{\overline{p}}, \Sigma _\mathrm{DFMT,\overline{p}}(z) \in \mathcal {L}\left( \mathcal {H}_{{\overline{p}}}\right)$. On the other hand, using again the Schur complement formula, we have

$$\begin{aligned} G_{\textrm{AI},p}(z)&= \left( G^0_{\textrm{AI},p}(z)^{-1} - \Sigma _{\textrm{AI},p}(z) \right) ^{-1} = \left( z-H^0_{\textrm{AI},p} - \Sigma _{\textrm{AI},p}(z) \right) ^{-1} \\&= \left( \begin{array}{cc} z-H^0_\textrm{p}-\Sigma _{\textrm{imp},p}(z) &  -V_{p} \\ -V_p^\dagger &  z-H^0_{\textrm{bath},p} \end{array}\right) ^{-1}\\&= \left( \begin{array}{cc} (z-H^0_\textrm{p}-\Sigma _{\textrm{imp},p}(z)-\Delta _p(z))^{-1} &  * \\ * &  * \end{array}\right) , \end{aligned}$$

so that

77 $$\begin{aligned} G_{\textrm{imp},p}(z) = (z-H^0_\textrm{p}-\Sigma _{\textrm{imp},p}(z)-\Delta _p(z))^{-1}. \end{aligned}$$

Conditions (73)–(74), together with (75)–(77) yield the self-consistent condition

78 $$\begin{aligned} \forall 1 \le p \le P, \quad \Delta _p(z) = W_p \left( z-H^0_{\overline{p}} - \bigoplus _{q=1,q \ne p}^P\Sigma _{\textrm{imp},q}(z) \right) ^{-1} W_p^\dagger . \end{aligned}$$

Note that the matrices $W_p$, $H^0_{\bar{p}}$ depend only on the hopping matrix $T_{ }$ through

$$\begin{aligned} [W_p]_{i\sigma ,j\sigma '}&=\left\{ \begin{array}{ll} T_{{i,j}} \\ 0 \end{array} \begin{array}{ll} \text { if } i \in \Lambda _p, j \in \Lambda \setminus \Lambda _p, \{i,j\} \in E, \; \sigma =\sigma ', \\ \text { otherwise,} \end{array} \right. \\ [H^0_{\bar{p}}]_{i\sigma ,j\sigma '}&= \left\{ \begin{array}{ll} T_{{i,j}} \\ 0 \end{array} \begin{array}{ll} \text { if } i,j \in \Lambda \setminus \Lambda _p, \{i,j\} \in E, \; \sigma =\sigma ', \\ \text { otherwise,} \end{array} \right. \end{aligned}$$

where $i,j \in \Lambda$ denote site indices and $\sigma ,\sigma ' \in \{\uparrow ,\downarrow \}$ spin indices.

#### Remark A.13

This formulation of DMFT agrees with the original one [25, 27] in the translation-invariant setting where

79 $$\begin{aligned} \forall p \in [\![ {1},{P}]\!], \quad \mathcal {H}_{{p}}=\mathcal {H}_{{1}}, \quad H^0_p=H^0_1, \quad W_p=W_1, \quad H^0_{\overline{p}}=H^0_{{\overline{1}}}, \quad U_p=U_1. \end{aligned}$$

In this setting, we can consider translation-invariant solutions to the DMFT equations, for which

$$\begin{aligned} \forall p \in [\![ {1},{P}]\!], \quad \forall z \in \mathbb {C}_+, \quad \Sigma _{\textrm{imp},p}(z)&=\Sigma _{\textrm{imp}}(z), \quad \Delta _p(z)=\Delta (z), \end{aligned}$$

where $\Sigma _{\textrm{imp}}(z)$ and $\Delta (z)$ are related by the translation-invariant self-consistent condition

80 $$\begin{aligned} \Delta (z)=W_1 \left( z-H^0_{\bar{1}} -\bigoplus _{q=2}^{P} \Sigma _{\textrm{imp}}(z)\right) ^{-1}W_1^\dagger . \end{aligned}$$

This setting is sketched in Fig. 4. When $\mathfrak {P}$ is a partition into singletons (single-site DMFT), condition (79) is equivalent to the fact that $U_{ }$ is constant and $(\mathcal {G}_{{H}},T_{ })$ is *vertex-transitive*, namely that for all $\lambda _1,\lambda _2 \in \Lambda$, there exists a graph isomorphism $\tau :\Lambda \rightarrow \Lambda$ such that

$$\begin{aligned} \tau (\lambda _1)=\lambda _2, \quad \forall \lambda ,\lambda ' \in \Lambda , \quad T_{{\tau (\lambda ),\tau (\lambda ')}}=T_{{\lambda ,\lambda '}} \end{aligned}$$

In particular, the graph Cartesian product $C_{N}^{\square d}$ of *d* copies of the *N*-cycle, which is the nearest neighbor graph of a truncation of the *d*-dimensional square lattice, with constant hopping and on-site repulsion, and artificial periodic boundary conditions (supercell method), is vertex-transitive due to the translation invariance of the corresponding lattice. This setting was the one considered in the first DMFT computations [27]. Due to translation invariance, a single impurity problem has to be solved at each iteration. Recall that the impurity solver is the computational bottleneck in DMFT.

The DMFT self-consistent equations (78), combined with an exact impurity solver, give the exact Green’s function of the original Hubbard model in the following trivial limits.

#### Proposition A.14

(Exactness of DMFT in some trivial limits). Consider a Hubbard model $(\mathcal {G}_{{H}},T_{ },U_{ })$ in a Gibbs state $\Omega _{\beta ,\epsilon _F}$. The self-consistent DMFT equations (78), combined with an exact impurity solver, admit a unique solution in each of the following two settings:

1. Non-interacting particles. If the on-site repulsion term is equal to zero ($U_{{i}}=0$ for all $i \in \Lambda$), then this solution is given by
  81 $$\begin{aligned} \forall p \in [\![ {1},{P}]\!], \quad \forall z \in \mathbb {C}_+, \quad \Sigma _{\textrm{imp},p}(z)&=0, \nonumber \\ \Delta _p(z)&= W_p \left( z-H^0_{\overline{p}}\right) ^{-1} W_p^\dagger ; \end{aligned}$$
2. Disconnected graphs and atomic limit. If the partition $\mathfrak {P}$ of $\mathcal {G}_{{H}}$ is such that $\mathcal {G}_{{H}}=\bigoplus _{p=1}^P\mathcal {G}_{{p}}$ (meaning $E_H=\sqcup _{p=1}^PE_p$), that is if the partition decomposes the original Hubbard model $(\mathcal {G}_{{H}},T_{ },U_{ })$ into *P* independent Hubbard models $(\mathcal {G}_{{p}},T_{{p}},U_{{p}})$, or if the hopping matrix $T_{ }$ vanishes, then this solution is given by
  82 $$\begin{aligned} \forall p \in [\![ {1},{P}]\!], \quad \forall z \in \mathbb {C}_+, \quad \Sigma _{\textrm{imp},p}(z)&=\Sigma _{p}(z), \nonumber \\ \Delta _p(z)&=0, \end{aligned}$$
  where $\Sigma _{p}$ is the exact self-energy associated with the *p*-th Hubbard model in the associated Gibbs state $\Omega _{\beta ,\epsilon _F}^p$.

In both settings, DMFT is exact in the sense that

$$\begin{aligned} \forall z \in \mathbb {C}_+, \quad G_{\textrm{DMFT}}(z)=G(z). \end{aligned}$$

The purpose of DMFT [25] is to build an appoximation that complies with these two limiting cases. Another limiting case in which DMFT is claimed to be exact is in the “infinite dimension” limit [27, 53]. We leave the mathematical investigation of this limit to future works.

We deduce from (81)–(82) that in the trivial limits considered in Proposition A.14, the hybridization functions $\Delta _p(z)$ are either identically zero, or have a finite number of poles, so that the corresponding baths can be chosen finite dimensional.

### A Specific Impurity Solver: The Iterated Perturbation Theory (IPT) Solver.

To define properly the IPT solver, we need first to introduce *Matsubara*’s formalism, and more precisely *Matsubara’s Green’s function* $\widetilde{G}^M$.

#### Matsubara’s Green’s functions

Matsubara’s Green’s functions are defined only for *Gibbs states* $\Omega _{\beta ,\epsilon _F}$ at a given inverse temperature $\beta$ and chemical potential $\epsilon _F$. These functions have been more extensively studied mathematically than time-ordered Green’s functions [12, 13]. In this section, we recall their definition and state an analytic continuation result based on Theorem B.9, the latter being useful for our analysis. The setup remains the same as the one introduced in Sect. A.1.

##### Definition A.15

*(Gibbs’s state and Matsubara’s time-ordered Green’s function).* Given a particle-number conserving Hamiltonian $\widehat{H}\in \mathcal {S}(\mathcal {F})$, that is $[{\widehat{H}},{\widehat{N}}]=0$, the *Gibbs state* $\Omega _{\beta ,\epsilon _F}$ at inverse temperature $\beta \in \mathbb {R}_+^*$ and chemical potential $\epsilon _F\in \mathbb {R}$ is defined through its density operator by

$$\begin{aligned} \widehat{\rho }=\frac{1}{Z_{\beta ,\epsilon _F}} e^{-\beta \left( \widehat{H}-\epsilon _F\widehat{N}\right) }, \quad Z_{\beta ,\epsilon _F}= \textrm{Tr}\left( e^{-\beta \left( \widehat{H}-\epsilon _F\widehat{N}\right) }\right) . \end{aligned}$$

The *Matsubara’s Green’s function* $\widetilde{G}^M$ is defined as the $\mathcal {L}(\mathcal {H}_{ })$-valued map $\widetilde{G}^M:[-\beta ,\beta ) \rightarrow \mathcal {L}(\mathcal {H}_{ })$ represented by the sesquilinear form verifying for all $\phi ,\phi ' \in \mathcal {H}_{ }$,

83 $$\begin{aligned}  &   \forall \tau \in [-\beta ,\beta ), \quad \langle \phi ,-\widetilde{G}^M(\tau )\phi ' \rangle = 1\!\!1_{{\mathbb {R}_+}}(\tau ) \, \Omega _{\beta ,\epsilon _F}\left( \mathbb {M}({\widehat{a}_{{\phi }}})(\tau ) \widehat{a}_{{\phi '}}^\dagger \right) \nonumber \\  &   - 1\!\!1_{{\mathbb {R}_-^*}}(\tau ) \, \Omega _{\beta ,\epsilon _F}\left( \widehat{a}_{{\phi '}}^\dagger \mathbb {M}({\widehat{a}_{{\phi }}})(\tau )\right) , \end{aligned}$$

where for all $\widehat{O}\in \mathcal {L}(\mathcal {F})$,

$$\begin{aligned} \mathbb {M}({\widehat{O}}):[-\beta ,\beta ] \ni \tau \mapsto e^{\tau (\widehat{H}-\epsilon _F\widehat{N})}\widehat{O}e^{-\tau (\widehat{H}-\epsilon _F\widehat{N})} \end{aligned}$$

is the *Matsubara picture* of $\widehat{O}$.

Recall that in our setting, any operator is trace-class since $\mathcal {F}$ is finite dimensional, hence $Z_{\beta ,\epsilon _F}$ is always finite.

As it is the case for the time-ordered Green’s function $\widetilde{G}$, one can recast equation (83) using the time-ordered product:

$$\begin{aligned} \forall \phi ,\phi ' \in \mathcal {H}_{ }, \ \forall \tau \in [-\beta ,\beta ), \quad \langle \phi ,-\widetilde{G}^M(\tau ) \phi ' \rangle =\Omega _{\beta ,\epsilon _F}\left( \mathcal {T}\left( \mathbb {M}({\widehat{a}_{{\phi }}}),\mathbb {M}({\widehat{a}_{{\phi '}}^\dagger })\right) (\tau ,0)\right) . \end{aligned}$$

Note that the negative sign in the definition of $\widetilde{G}^M$ must be consistent with the *i* prefactor in the definition of $\widetilde{G}$ for Theorem A.17 below to hold. Considering the grand canonical Hamiltonian $\widehat{H}'=\widehat{H}-\epsilon _F\widehat{N}$ as the Hamiltonian from which the Green’s function $\widetilde{G}$ is defined, the Matsubara formalism involves the following formal connection (known as *Wick rotation* [75]):

$$\begin{aligned} \tau \leftrightarrow it \end{aligned}$$

or, in other words, working with $t \leftrightarrow -i \tau$ where $\tau$ is real. Hence, the term *imaginary-time* Green’s function [52].

Contrary to the Heisenberg picture $\widehat{O}\mapsto \mathbb {H}({\widehat{O}})$, the Matsubara picture does not consist in a family of $C^*$-morphisms: one has for all $\tau \in [-\beta ,\beta ]$,

$$\begin{aligned} \left( \mathbb {M}({\widehat{O}})(\tau )\right) ^\dagger = \mathbb {M}({\widehat{O}^\dagger })(-\tau ). \end{aligned}$$

A consequence of this property is that the Matsubara’s Green’s function is Hermitian:

$$\begin{aligned} \left( \widetilde{G}^M(\tau )\right) ^\dagger =\widetilde{G}^M(\tau ). \end{aligned}$$

Note moreover that Gibbs states are *Kubo-Martin-Schwinger (KMS)* states [13], meaning that they satisfy the following property:

$$\begin{aligned} \forall \ \widehat{O},\widehat{O}' \in \mathcal {L}(\mathcal {F}),\ \forall \tau \in [-\beta ,0], \quad \Omega _{\beta ,\epsilon _F}(\mathbb {M}({\widehat{O}})(\tau + \beta )\widehat{O}')=\Omega _{\beta ,\epsilon _F}(\widehat{O}'\mathbb {M}({\widehat{O}})(\tau )). \end{aligned}$$

This implies that $\widetilde{G}^M$ is $\beta$-*anti-periodic*, i.e.

$$\begin{aligned} \forall \tau \in [-\beta ,0), \quad \widetilde{G}^M(\tau + \beta )=-\widetilde{G}^M(\tau ). \end{aligned}$$

As for the time-ordered Green’s function, the Matsubara’s Green’s function has a Källén-Lehmann’s representation: given an orthonormal basis $\mathcal {B}$ of $\mathcal {F}$ which jointly diagonalizes $\widehat{H}$ and $\widehat{N}$ ($\forall \psi \in \mathcal {B}, \widehat{H}\psi = E_\psi \psi , \; \widehat{N}\psi = N_\psi \psi$), we have for all $\tau \in [0,\beta )$, for all $\phi ,\phi ' \in \mathcal {H}_{ }$,

$$\begin{aligned} \langle \phi ,-\widetilde{G}^M(\tau ) \phi ' \rangle&= \sum _{\psi ,\psi ' \in \mathcal {B}} e^{\tau ((E_\psi -\epsilon _FN_\psi )--(E_{\psi '} - \epsilon _FN_{\psi '}))} \langle \psi ,\widehat{a}_{{\phi }}\psi ' \rangle \langle \psi ',\widehat{a}_{{\phi '}}^\dagger \psi \rangle e^{-\beta (E_\psi - \epsilon _FN_\psi )} \\  &=\sum _{\psi ,\psi ' \in \mathcal {B}} e^{\tau (E_\psi -E_{\psi '} + \epsilon _F)} \langle \psi ,\widehat{a}_{{\phi }}\psi ' \rangle \langle \psi ',\widehat{a}_{{\phi '}}^\dagger \psi \rangle e^{-\beta (E_\psi - \epsilon _FN_\psi )}, \end{aligned}$$

as $$N_{\psi '}=N_\psi +1$$ whenever $$\langle \psi ',\widehat{a}_{{\phi '}}^\dagger \psi \rangle \ne 0$$.

Similarly to the time-ordered Green’s function $\widetilde{G}$, it is convenient to work with a Fourier representation of the Matsubara Green’s function $\widetilde{G}^M$. Since the latter is defined only on $[-\beta ,\beta )$, it is quite natural to extend $\widetilde{G}^M$ to the real-line by periodicity and introduce the associated Fourier series with coefficients defined by

$$\begin{aligned} \forall n \in \mathbb {Z}, \quad \frac{1}{2}\int _{-\beta }^{\beta }\widetilde{G}^M(\tau ) e^{in\frac{\pi }{\beta }\tau }d\tau . \end{aligned}$$

Due to the $\beta$-anti-periodicity of $\widetilde{G}^M$, the even Fourier coefficients vanish and it holds

$$\begin{aligned} \frac{1}{2}\int _{-\beta }^{\beta }\widetilde{G}^M(\tau ) e^{in\frac{\pi }{\beta }\tau }d\tau = \left\{ \begin{array}{ll} \int _{0}^\beta \widetilde{G}^M(\tau ) e^{in\frac{\pi }{\beta }\tau }d\tau &  \text {if} n \text {is odd,} \\ 0 &  \text { otherwise.} \end{array} \right. \end{aligned}$$

##### Definition A.16

*(Matsubara’s Fourier series and frequencies).* The *Matsubara’s Fourier coefficients* $\left( G^M_n\right) _{n \in \mathbb {Z}}$ are defined by

$$\begin{aligned} \forall n \in \mathbb {Z}, \quad G^M_n=\int _0^\beta \widetilde{G}^M(\tau ) e^{i\omega _n\tau } d\tau \end{aligned}$$

where $\omega _n=(2n+1)\frac{\pi }{\beta }$ is the *n*-th *Matsubara’s frequency*.

We thus have

$$\begin{aligned} \forall \tau \in [-\beta ,\beta ), \quad \widetilde{G}^M(\tau )=\frac{1}{\beta } \sum _{n \in \mathbb {Z}}e^{-i\omega _n\tau } G^M_n. \end{aligned}$$

The very reason these coefficients are useful in Green’s functions methods lies in the following theorem.

##### Theorem A.17

(Matsubara’s Fourier coefficients analytic continuation). Let $\widehat{H}\in \mathcal {S}(\mathcal {F})$ be a particle-number-conserving Hamiltonian, $\Omega _{\beta ,\epsilon _F}$ the associated Gibbs state, and $G: \mathbb {C}_+\rightarrow \mathcal {L}(\mathcal {H}_{ })$ the Generalized Fourier Transform of the associated time-ordered Green’s function $\widetilde{G}$ defined from the grand canonical Hamiltonian $\widehat{H}'=\widehat{H}-\epsilon _F\widehat{N}$. Then $G$ is the only analytic matrix-valued function such that

84 $$\begin{aligned} \forall z\in \mathbb {C}_+, \ \Im (G(z)):=\frac{G(z)-G(z)^\dagger }{2i}\le 0, \text { and } \end{aligned}$$

85 $$\begin{aligned} \forall n \in \mathbb {N}, \quad G(i\omega _n)= G^M_n. \end{aligned}$$

Note that, since $\omega _{-n}=-\omega _{n-1}$ and $\widetilde{G}^M$ is Hermitian, it holds

$$\begin{aligned} \forall n \in \mathbb {N}^*, \quad \left( G^M_{n-1}\right) ^\dagger = G^M_{-n}, \end{aligned}$$

so that (85) actually holds for all $n \in \mathbb {Z}$, with the extension of $G$ to $\mathbb {C}_-$ defined as in (55).

##### Remark A.18

The requirement that $G$ is analytic and verifies (84) is crucial for uniqueness: for instance, for each $m \in 2\mathbb {Z}+1$, the function

$$\begin{aligned} \mathbb {C}_+\ni z \mapsto \frac{1-e^{m\beta z}}{2} G(z) \in \mathcal {L}(\mathcal {H}_{ }) \end{aligned}$$

also satisfies (85) and is analytic, but its imaginary part is not negative semi-definite.

Theorem A.17 is extensively used for practical computations: it is sufficient to run the computations for the Matsubara frequencies and then perform an analytic continuation [21].

Note that Theorem A.17 works in particular for non-interacting Hamiltonians, which leads to the following definition.

##### Definition A.19

*(Matsubara’s self-energy).* The *Matsubara’s self-energy Fourier coefficients* $\left( \Sigma ^M_n\right) _{n\in \mathbb {Z}}$ are defined by

$$\begin{aligned} \forall n \in \mathbb {Z}, \quad \Sigma ^M_n=i\omega _n +\epsilon _F-H^0- \left( G^M_n\right) ^{-1}. \end{aligned}$$

In fact, the self-energy $\Sigma : \mathbb {C}_+\rightarrow \mathcal {L}(\mathcal {H}_{ })$ defined in (61) is the only analytic function with negative imaginary part such that

$$\begin{aligned} \forall n \in \mathbb {N}, \quad \Sigma (i\omega _n)=\Sigma ^M_n. \end{aligned}$$

This follows from Proposition A.1 and Theorem B.9. One can define the *Matsubara’s self-energy* $\tilde{\Sigma }^M(\tau )$ by Fourier summation formula as in Definition A.16, but this function will not play any role in what follows.

With all these definitions in place, we can now introduce the final ingredient of the model under investigation, namely the single-site, translation-invariant, paramagnetic Iterated Perturbation Theory (IPT).

#### IPT approximation

In this article, we will not discuss the derivation nor the validity of the Iterative Perturbation Theory (IPT) approximation and refer the interested reader to [6]. As in the review paper [27], we only consider *single-site* and *translation-invariant* DMFT. This seems to be a setting in which the usual IPT approximation is generally considered as valid in the physics literature, while constructing an IPT-like approximation for multi-site DMFT is still an active field of research [6]. In the former case, $\mathfrak {P}$ is a partition of the *L* sites into $P=L$ singletons and the on-site repulsion $U_{ }$ is constant as exposed in remark A.13. Also, we will focus on the paramagnetic case [62]. In other words, we will assume that there is no spin-symmetry breaking, so that the spin components can be factored out as detailed in Appendix A.6. In the single-site, translation-invariant, paramagnetic IPT-DMFT approximation considered in the sequel, we thus have $P=L$ and for $z\in \mathbb {C}_+$,

$$\begin{aligned} H^0_\textrm{H},\ G^{{DMFT}}(z),\ \Sigma ^{{DMFT}}(z) \in \mathbb {C}^{P \times P}. \end{aligned}$$

Recall that in translation invariant DMFT, we restrict ourselves to translation invariant solutions to the DMFT equations, so that we consider only one hybridization function $\Delta$ and self-energy $\Sigma$, with for all $z\in \mathbb {C}_+$,

$$\begin{aligned} \Delta (z),\ \Sigma (z) \in \mathbb {C}, \end{aligned}$$

and we use the following notations

$$\begin{aligned} W^\dagger := W_p^\dagger \in \mathbb {C}^{P-1}, \quad H^0_\perp := H^0_{{\overline{p}}} \in \mathbb {C}^{(P-1) \times (P-1)}. \end{aligned}$$

Moreover, we stick to the *half-filled* setting [27], for which the chemical potential $\epsilon _F$ of the Anderson Impurity Model is set to $U_{ }/2$. The Hamiltonians of interest are then on the one-hand the grand canonical Hubbard Hamiltonian $\widehat{H}_{H}-\epsilon _F\widehat{N}_{H}$, and on the other hand the "impurity" grand canonical Hamiltonian ${\widehat{H}}_\textrm{AIM}-\epsilon _F\widehat{N}_\textrm{imp}$ where $\widehat{N}_\textrm{imp}$ is the impurity number operator, complying with [27, eq. 14].

The IPT solver is based on a second order *perturbation expansion* of the Matsubara’s self-energy Fourier coefficients $\Sigma ^M_{\textrm{imp},n}$ of the single-site impurity problem in the parameter $U_{ }$: the Matsubara’s self-energy Fourier coefficients of the impurity problem is approximated by

$$\begin{aligned} \forall n \in \mathbb {N}, \quad \Sigma ^M_{\textrm{imp},n} \approx \Sigma _{\textrm{imp},n}^{M,{{IPT}}}:=\frac{U_{ }}{2}+U_{ }^2\int _0^\beta e^{i\omega _n\tau } \left( \tilde{G}^{M,0}_{\textrm{imp}}(\tau )\right) ^3 d\tau , \end{aligned}$$

where $\tilde{G}^{M,0}_{\textrm{imp}}$ is the restriction to $\mathcal {H}_{{\textrm{imp}}}$ of the Matsubara’s Green’s function of the non-interacting Hamiltonian $H^0_{\textrm{AI}}$. The Fourier coefficients of $\tilde{G}^{M,0}_{\textrm{imp}}$ are given by

$$\begin{aligned} G^{M,0}_{\textrm{imp},n}= \left( \left( G_{\textrm{imp}}^0(i\omega _n)\right) ^{-1} - \epsilon _F\right) ^{-1} = \left( i\omega _n - H^0_{\textrm{imp}} - \Delta (i\omega _n) \right) ^{-1} =\left( i\omega _n - \Delta (i\omega _n) \right) ^{-1}, \end{aligned}$$

since $H^0_{\textrm{imp}}=0$ and where the first equality is a consequence of a shift to enforce particle-hole symmetry [27, p.50]. Finally, noticing that

86 $$\begin{aligned} \Delta (z)=W\left( z+\epsilon _F-H^0_{\perp } + \Sigma (z) \right) ^{-1}W^\dagger = W \left( z-H^0_{\perp } -(\Sigma (z)-\epsilon _F)\right) ^{-1}W^\dagger ,\qquad \end{aligned}$$

we make the change of variable $\Sigma \leftarrow \Sigma - \epsilon _F$ and we thus have, due to the filling condition,

87 $$\begin{aligned} \Sigma _{\textrm{imp},n}^{M,{IPT}}=U_{ }^2\int _{0}^\beta e^{i\omega _n \tau } \left( \frac{1}{\beta }\sum _{n' \in \mathbb {Z}}\left( i\omega _{n'} - \Delta (i\omega _{n'}) \right) ^{-1} e^{-i\omega _{n'} \tau } \right) ^3 d \tau . \end{aligned}$$

The IPT approximation therefore provides an explicit expression of the Matsubara Fourier coefficients of the impurity self-energy $\Sigma _{\textrm{imp}}$ as a function of $U_{ }$ and $\Delta$. To reconstruct $\Sigma ^{{IPT}}_{\textrm{imp}}(z)$ from these Fourier coefficients, we have to solve an Analytic Continuation Problem. The following result shows that, in the case of finite-dimensional baths, this problem has a unique solution, which can be computed (almost) explicitly. Our results are similar to computations already obtained in [37, eq. 4].

We denote by

$$\begin{aligned} \Sigma _\textrm{imp}^{{IPT}}=\mathrm {{IPT}}_\beta (U_{ },\Delta ) \end{aligned}$$

the output of this solver. At this time, this solver is defined only for finite baths, that is for hybridization functions that are rational functions of the form (13). We will see later (in Proposition 3.7) that this map can be extended by (weak) continuity to the space of all physically admissible hybridization functions. We thus finally obtain the system of single-site, translation-invariant, paramagnetic IPT-DMFT equations

88 $$\begin{aligned} \forall z \in \mathbb {C}_+, \quad \Delta (z)&=W\left( z-H^0_{\perp } - \Sigma (z) \right) ^{-1}W^\dagger \end{aligned}$$

89 $$\begin{aligned} \Sigma&=\mathrm {{IPT}}_\beta (U_{ },\Delta ) , \end{aligned}$$

where the on-site interaction $U\in \mathbb {R}$, the inverse temperature $\beta > 0$, the vector $W^\dagger \in \mathbb {C}^{P-1}$ and the matrix $H^0_{\perp } \in \mathbb {C}^{(P-1)\times (P-1)}$ obtained from the hopping matrix *T* are the parameters of the model, and where $\Delta :\mathbb {C}_+\rightarrow \mathbb {C}$ and $\Sigma :\mathbb {C}_+\rightarrow \mathbb {C}$ are the unknowns.

In the remainder of this article, our main focus will be the existence of solutions to the above equations.

### Paramagnetic IPT-DMFT Equations

In this appendix, we detail the spin independence of the paramagnetic IPT-DMFT equations.

As detailed in Remark A.11, the Hamiltonian ${\widehat{H}}_\textrm{AIM}$ commutes with the total spin operator $\widehat{S}_{AI}$. More precisely, $\mathcal {H}_{{AI}}$ is $\widehat{S}_{AI}$-invariant, and in the decomposition

90 $$\begin{aligned}&\mathcal {H}_{{AI}}=\mathcal {H}_{{\uparrow }}\oplus \mathcal {H}_{{\downarrow }}, \quad \mathcal {H}_{{\sigma }}\nonumber \\&\quad =\textrm{Span}\left( \vert \emptyset \rangle \otimes \cdots \otimes \vert \emptyset \rangle \otimes \underset{m\text {-th}}{\vert \sigma \rangle } \otimes \vert \emptyset \rangle \otimes \cdots \otimes \vert \emptyset \rangle , m \in \Lambda \sqcup [\![ {1},{B}]\!]\right) \end{aligned}$$

the total spin operators reads $\widehat{S}_{AI}=\textbf{1}_{\uparrow }\oplus (-\textbf{1}_{\downarrow })$:

$$\begin{aligned} \widehat{S}_{AI}=\left( \begin{array}{ll} \textbf{1}& \quad 0 \\ 0& \quad -\textbf{1}\end{array} \right) . \end{aligned}$$

Writing in this decomposition $H^0=H^0_{\uparrow }\oplus H^0_{\downarrow }$, we then have

$$\begin{aligned} G^0(z)=(z-H^0_\uparrow )^{-1}\oplus (z-H^0_\downarrow )^{-1}. \end{aligned}$$

Since no magnetic field is included in the model, $H^0_\uparrow$ and $H^0_\downarrow$ act the same way on their respective domains: denoting by $\widehat{F}\in \mathcal {L}(\mathcal {H}_{{AI}})$ the *spin flip* isomorphism defined by linearity with $\forall m \in \Lambda \sqcup [\![ {1},{B}]\!],\forall \sigma \in \{\uparrow ,\downarrow \}$

$$\begin{aligned} \widehat{F}\left( \vert \emptyset \rangle \otimes \cdots \otimes \vert \emptyset \rangle \otimes \underset{m\text {-th}}{\vert \sigma \rangle } \otimes \vert \emptyset \rangle \otimes \cdots \otimes \vert \emptyset \rangle \right) = \vert \emptyset \rangle \otimes \cdots \otimes \vert \emptyset \rangle \otimes \underset{m\text {-th}}{\vert \bar{\sigma } \rangle } \otimes \vert \emptyset \rangle \otimes \cdots \otimes \vert \emptyset \rangle , \end{aligned}$$

we have

$$\begin{aligned} H^0_{\uparrow }=\widehat{F}_{\uparrow ,\downarrow } H^0_{\downarrow } \widehat{F}_{\downarrow ,\uparrow }. \end{aligned}$$

Now in the impurity-spin decomposition

91 $$\begin{aligned} \mathcal {H}_{ }&=\mathcal {H}_{{\uparrow ,\textrm{imp}}}\oplus \mathcal {H}_{{\uparrow ,\textrm{bath}}}\oplus \mathcal {H}_{{\downarrow ,\textrm{imp}}}\oplus \mathcal {H}_{{\downarrow ,\textrm{bath}}} \end{aligned}$$

92 $$\begin{aligned} \mathcal {H}_{{\sigma ,imp}}&= \textrm{Span}\left( \vert \emptyset \rangle \otimes \cdots \otimes \vert \emptyset \rangle \otimes \underset{i\text {-th}}{\vert \sigma \rangle } \otimes \vert \emptyset \rangle \otimes \cdots \otimes \vert \emptyset \rangle , i \in \Lambda \right) \end{aligned}$$

93 $$\begin{aligned} \mathcal {H}_{{\sigma ,bath}}&= \textrm{Span}\left( \vert \emptyset \rangle \otimes \cdots \otimes \vert \emptyset \rangle \otimes \underset{k\text {-th}}{\vert \sigma \rangle } \otimes \vert \emptyset \rangle \otimes \cdots \otimes \vert \emptyset \rangle , k \in [\![ {1},{B}]\!] \right) \end{aligned}$$

we have $\widehat{S}_{AI}=\textbf{1}\oplus \textbf{1}\oplus (-\textbf{1})\oplus (-\textbf{1})$ and $\widehat{N}_\textrm{imp}=\textbf{1}\oplus 0 \oplus \textbf{1}\oplus 0$:

$$\begin{aligned} \widehat{S}_{AI}=\left( \begin{array}{llll} \textbf{1}& \quad 0& \quad 0& \quad 0 \\ 0& \quad \textbf{1}& \quad 0& \quad 0 \\ 0& \quad 0& \quad -\textbf{1}& \quad 0 \\ 0& \quad 0& \quad 0& \quad -\textbf{1}\end{array} \right) ,\quad \widehat{N}_\textrm{imp}=\left( \begin{array}{llll} \textbf{1}& \quad 0& \quad 0& \quad 0\\ 0& \quad 0& \quad 0& \quad 0 \\ 0& \quad 0& \quad \textbf{1}& \quad 0\\ 0& \quad 0& \quad 0& \quad 0 \end{array}\right) \end{aligned}$$

and we have for the impurity orthogonal restriction of the non-interacting Green’s function $G_\textrm{imp}^0$:

$$\begin{aligned} G_\textrm{imp}^0(z)=\left( z-H^0_\mathrm {\uparrow ,\textrm{imp}}-\Delta _\uparrow (z)\right) \oplus \left( z-H^0_\mathrm {\downarrow ,\textrm{imp}}-\Delta _\downarrow (z)\right) \end{aligned}$$

Indeed, as it is the case for the non-interacting Hamiltonian and the non-interacting Green’s function, $\Delta _{\uparrow }$ acts similarly as $\Delta _{\downarrow }$ on their respective domain, and are related by conjugation with the spin flip operator

$$\begin{aligned} \Delta _{\uparrow }=\widehat{F}_{\uparrow ,\downarrow }\Delta _{\downarrow }\widehat{F}_{\downarrow ,\uparrow } \end{aligned}$$

With that being said, in the IPT approximation outlined in this document, we have for $n \in \mathbb {Z}$,

$$\begin{aligned} \Sigma ^M_{\textrm{imp},n}=\Sigma ^M_{\uparrow ,\textrm{imp},n} \oplus \Sigma ^M_{\downarrow ,\textrm{imp},n}, \end{aligned}$$

with for all $\sigma \in \{\uparrow ,\downarrow \},$

$$\begin{aligned} \Sigma ^M_{\sigma ,\textrm{imp},n}=U_{ }^2\int _{0}^\beta e^{i\omega _n \tau } \left( \frac{1}{\beta }\sum _{n' \in \mathbb {Z}}\left( i\omega _{n'} - \Delta _{\sigma }(i\omega _{n'})^{-1} \right) \right) ^3 d \tau . \end{aligned}$$

And indeed, we have the conjugation relation

$$\begin{aligned} \Sigma ^M_{\uparrow ,\textrm{imp},n} =\widehat{F}_{\uparrow ,\downarrow }\Sigma ^M_{\downarrow ,\textrm{imp},n}\widehat{F}_{\downarrow ,\uparrow }. \end{aligned}$$

These last equations show that the impurity-spin orthogonal restrictions of the Matsubara self-energy Fourier coefficients are copies one of another. Since we assume that the partition $\mathfrak {P}$ is in singletons, it follows that $\vert {\Lambda }\vert =1$ and $\dim (\mathcal {H}_{{\sigma ,\textrm{imp}}})=1$, so that using the bases $\mathcal {B}_{{\sigma ,\textrm{imp}}}$ given by

$$\begin{aligned} \forall \sigma \in \{\uparrow ,\downarrow \}, \quad \mathcal {B}_{{\sigma ,\textrm{imp}}}=\{ \vert \sigma \rangle \otimes \vert \emptyset \rangle \otimes \cdots \otimes \vert \emptyset \rangle \}, \end{aligned}$$

we have for all $n \in \mathbb {Z}$,

$$\begin{aligned} \quad \textrm{mat}_{\mathcal {B}_{{\uparrow ,\textrm{imp}}}}(\Sigma ^M_{\uparrow ,\textrm{imp},n})=\textrm{mat}_{\mathcal {B}_{{\downarrow ,\textrm{imp}}}}(\Sigma ^M_{\downarrow ,\textrm{imp},n}):= \Sigma _n. \end{aligned}$$

Finally, we have for $z \in \mathbb {C}_+$,

$$\begin{aligned} \textrm{mat}_{\mathcal {B}_{{\uparrow ,\textrm{imp}}}}(\Delta _{\uparrow }(z))=\textrm{mat}_{\mathcal {B}_{{\downarrow ,\textrm{imp}}}}(\Delta _\downarrow (z))=\sum _{k=1}^B\frac{V_{{k}} V_{{k}}^\dagger }{z-\epsilon _{{k}}}, \end{aligned}$$

so that we indifferently refer to $\Delta (z)$ by abuse of notation.

### Proofs of the Results in Appendix A

Most of the results presented in Appendix A are known in settings similar to ours. However, the proofs of Propositions A.4 and A.10 available in the literature typically apply only to specific classes of states (such as ground states or Gibbs states) and do not emphasize the importance of the notion of *steady* state. Our proofs remove this artificial distinction. Additionally, Proposition A.14 is often treated as obvious in the physics literature, although it is usually proven only in translation-invariant settings. Our proof facilitates the understanding of the DMFT equations, and we hope it will help the reader grasp the machinery introduced in this section.

Furthermore, Theorem A.17 has long been considered proven in [8] within the physics community. However, the proof given in [8] does not make use of the classical analytic continuation techniques already available at the time. Our proof is entirely different and relies on Nevanlinna-Pick analytic continuation theory (especially on Theorem B.9, see Appendix B for an introduction to this subject). We hope it sheds light on certain aspects of [8] in the specific *finite*-dimensional Hilbert space setting considered here. In particular, our proof of the uniqueness of the analytic continuation of the Matsubara’s Fourier coefficients does not rely on the asymptotic behavior of the Green’s function.

#### Proof of Proposition A.4

Our proof is based on the time evolution formula of annihilation/creation propagators for ideal Fermi gases: one has, as detailed in [13, p.46],

94 $$\begin{aligned} \mathbb {H}({\widehat{a}_{{\phi }}})(t)=\widehat{a}_{{e^{itH^0}\phi }}. \end{aligned}$$

For all $z \in \mathbb {C}_+$, we have to show that $(z-H^0)G(z)=(z-H^0)(G_{+}(z)+G_{-}(\bar{z})^\dagger )=\textbf{1}$. Integrating by parts, one has

$$\begin{aligned} (z-H^0)G_{+}(z)=i\widetilde{G}(0^+)+\int _{\mathbb {R}_+^*}e^{izt}\left( i\frac{d}{dt}\widetilde{G}(t)-H^0\widetilde{G}(t)\right) dt, \end{aligned}$$

and for $t > 0$, $i\widetilde{G}(t)$ represents the sesquilinear form defined by

$$\begin{aligned} \forall \phi ,\phi ' \in \mathcal {H}_{ }, \quad \langle \phi ,(i\widetilde{G})(t)\phi ' \rangle =\Omega (\mathbb {H}({\widehat{a}_{{\phi }}})(t)\widehat{a}_{{\phi '}}^\dagger ), \end{aligned}$$

so that

$$\begin{aligned}  &   \langle \phi ,(i\frac{d}{dt}\widetilde{G})(t)\phi ' \rangle =\Omega (\frac{d}{dt}(\mathbb {H}({\widehat{a}_{{\phi }}}))(t)\widehat{a}_{{\phi '}}^\dagger )\\  &   =-i\Omega (\mathbb {H}({\widehat{a}_{{H^0\phi }}})(t)\widehat{a}_{{\phi '}}^\dagger )=\langle H^0\phi ,\widetilde{G}(t)\phi ' \rangle = \langle \phi ,H^0\widetilde{G}(t)\phi ' \rangle . \end{aligned}$$

Similarly, another integration by parts leads to

$$\begin{aligned} (z-H^0)G_{-}(\bar{z})^\dagger =i\widetilde{G}(0^-)^\dagger + \int _{\mathbb {R}_{-}}e^{-izt}(i\frac{d}{dt}\widetilde{G}(t)-\widetilde{G}(t) H^0)^\dagger dt. \end{aligned}$$

For $t<0$, $i\widetilde{G}(t)$ represents the sesquilinear form defined by

$$\begin{aligned} \forall \phi ,\phi ' \in \mathcal {H}_{ }, \langle \phi ,(i\widetilde{G})(t)\phi ' \rangle =-\Omega (\widehat{a}_{{\phi '}}^\dagger \mathbb {H}({\widehat{a}_{{\phi }}})(t)). \end{aligned}$$

Note that, because $\Omega$ is a steady state and by the cyclic property of the trace, one has

95 $$\begin{aligned} \langle \phi ,(i\widetilde{G})(t)\phi ' \rangle =-\Omega (\mathbb {H}({\widehat{a}_{{\phi '}}^\dagger })(-t)\widehat{a}_{{\phi }}) \end{aligned}$$

(steady state propagators are time-translation-invariant), so that

$$\begin{aligned} \langle \phi ,(i\frac{d}{dt}\widetilde{G})(t)\phi ' \rangle = i \Omega (\mathbb {H}({\widehat{a}_{{H^0\phi '}}^\dagger })(-t)\widehat{a}_{{\phi }})=\langle \phi ,\widetilde{G}(t)H^0\phi ' \rangle . \end{aligned}$$

Finally, one has for $\phi ,\phi ' \in \mathcal {H}_{ }$,

$$\begin{aligned} \langle \phi ,(z-H^0)G(z)\phi ' \rangle =\langle \phi ,i\widetilde{G}(0^+) \phi ' \rangle + \langle \phi ,i\widetilde{G}(0^{-})^\dagger \phi ' \rangle =\Omega (\widehat{a}_{{\phi }}\widehat{a}_{{\phi '}}^\dagger +\widehat{a}_{{\phi '}}^\dagger \widehat{a}_{{\phi }}) = \langle \phi ,\phi ' \rangle , \end{aligned}$$

because $\Omega$ is a state and the annihilation/creation operators satisfy the CAR.

#### Proof of Proposition A.1 ($-G, -\Sigma , -\Delta$ are Pick matrices)

The fact that the Green’s function $G$ is a Pick matrix readily follows from the KL representation (56) and inequality (57).

Combining (59) and (61), the self-energy can be written as

$$\begin{aligned} \forall z \in \mathbb {C}_+, \quad \Sigma (z)= z-H^0 - G(z)^{-1}. \end{aligned}$$

Recall that we know from (57) that *G*(*z*) is invertible for all $z \in \mathbb {C}_+$. Since $-G$ is Pick, $G^{-1}$ is Pick. This readily implies that $\Sigma$ is analytic. Let us now prove that $-\Sigma$ is Pick. First, we infer from the KL representation (56) that for all $k \in \mathbb {N}$, there exists $m_0,\dots ,m_{2k}\in \mathbb {C}^{n\times n}$ such that it holds

$$\begin{aligned} G(iy) =\frac{m_0}{iy} + \frac{m_1}{(iy)^2} +\frac{m_2}{(iy)^3}+\dots +\frac{m_{2k}}{(iy)^{2k+1}}+o_{y \rightarrow +\infty } \left( \frac{1}{y^{2k+1}}\right) . \end{aligned}$$

Using the anti-commutation relation $\widehat{a}_{{\phi }}\widehat{a}_{{\phi '}}^\dagger + \widehat{a}_{{\phi '}}^\dagger \widehat{a}_{{\phi }}=\langle \phi ,\phi ' \rangle$ and the normalization condition $\sum _{\psi \in \mathcal {B}} \rho _\psi = 1$, we obtain that $m_0=I_n$. As a consequence, we have

$$\begin{aligned} G(iy)^{-1} = (iy) I_n -m_1 + \frac{1}{iy}(m_1^2-m_2) + o_{y \rightarrow +\infty }\left( \frac{1}{y} \right) . \end{aligned}$$

In view of Theorem 4.1, the Pick matrix $G^{-1}$ has a Nevanlinna-Riesz representation of the form

$$\begin{aligned} G(z)^{-1} = z - H^0- \Sigma _\infty +\int _{\mathbb {R}} \frac{d\mu (\epsilon )}{\epsilon -z}, \end{aligned}$$

with $\Sigma _\infty \in \mathcal {S}_n(\mathbb {C})$ and $\mu$ a finite Borel $\mathcal {S}_n^+(\mathbb {C})$-valued measure on $\mathbb {R}$. We thus obtain that

$$\begin{aligned} \forall z \in \mathbb {C}_+, \quad \Sigma (z) = \Sigma _\infty +\int _{\mathbb {R}} \frac{d\mu (\epsilon )}{z-\epsilon }, \end{aligned}$$

from which we deduce that $-\Sigma$ is Pick. As a matter of fact, $\Sigma$ is a matrix-valued rational function, that is $\mu$ is a weighted sum of finitely many Dirac measures.

Let $\Delta$ be a hybridization function of an AIM defined as (67). Since $H^0_\textrm{bath}$ is self-adjoint, its spectrum is real and (67) thus defines an analytic function on $\mathbb {C}_+$. In addition, for all $z\in \mathbb {C}_+$, $\Im (z-H^0_\textrm{bath})=\Im (z)>0$ hence $\Im ((z-H^0_\textrm{bath})^{-1}) <0$. Since the congruence preserves the sign of the imaginary part we have $\Im (\Delta (z))\le 0$. This shows that $-\Delta$ is a Pick matrix.

#### Proof of Theorem A.10

For the simplicity of the proof, note first that an equivalent definition of an impurity problem is that there exists an *impurity space* $\mathcal {H}_{{\textrm{imp}}}\subset \mathcal {H}_{ }$ such that the interacting part $\widehat{H}^I$ of the Hamiltonian $\widehat{H}$ as introduced in (58) belongs to the following subalgebra

96 $$\begin{aligned} \widehat{H}^I\in \mathcal {A}\{\widehat{a}_{{\phi }}^\dagger \widehat{a}_{{\phi '}}, \phi ,\phi ' \in \mathcal {H}_{{\textrm{imp}}}\}. \end{aligned}$$

In other words, the interacting part of the Hamiltonian is an element of the Gauge Invariant Canonical Anti-commutation Relations (GICAR) algebra generated by $\mathcal {H}_{{\textrm{imp}}}$ (see [67] for a concise introduction to GICAR algebras).

From that, the proof is a generalization of [49]. To prove the sparsity pattern of $\Sigma$, we have to prove that for all $z \in \mathbb {C}_+$, for all $\phi \in \mathcal {H}_{ }, \phi ' \in \mathcal {H}_{{\textrm{imp}}}^\perp$,

97 $$\begin{aligned} \langle \phi ,G(z)\Sigma (z)\phi ' \rangle =0 \text { and } \langle \phi ',\Sigma (z)G(z)\phi \rangle =0. \end{aligned}$$

The first equality is equivalent to

$$\begin{aligned} \langle \phi ,G(z)(z-H^0)\phi ' \rangle =\langle \phi ,\phi ' \rangle , \end{aligned}$$

and, similarly as in the previous proof, we have

$$\begin{aligned} \langle \phi ,G(z)(z-H^0)\phi ' \rangle =\langle \phi ,\phi ' \rangle&+\int _{\mathbb {R}_+}e^{izt}\left( \frac{d}{dt}\langle \phi ,i\widetilde{G}(t)\phi ' \rangle - \langle \phi ,\widetilde{G}(t)H^0\phi ' \rangle \right) dt \\&+\int _{\mathbb {R}_-^*} e^{-izt}\left( \frac{d}{dt}\langle i\widetilde{G}(t)\phi ,\phi ' \rangle -\langle \widetilde{G}(t)H^0\phi ,\phi ' \rangle \right) dt. \end{aligned}$$

Now, for all $t \in \mathbb {R}_+$, we have using the cyclicity of the trace, similarly as in (95),

$$\begin{aligned} \frac{d}{dt}\langle \phi ,i\widetilde{G}(t)\phi ' \rangle =-i \Omega \left( \widehat{a}_{{\phi }}\mathbb {H}({[{\widehat{H}},{\widehat{a}_{{\phi '}}^\dagger }]})(-t)\right) . \end{aligned}$$

To compute the commutator, note first that for all $\phi _1,\phi _2 \in \mathcal {H}_{{\textrm{imp}}}$, we have using the CAR

$$\begin{aligned} [{\widehat{a}_{{\phi _1}}^\dagger \widehat{a}_{{\phi _2}}},{\widehat{a}_{{\phi '}}^\dagger }]=\langle \phi _2,\phi ' \rangle \widehat{a}_{{\phi _1}}^\dagger =0 \end{aligned}$$

since $\phi ' \in \mathcal {H}_{{\textrm{imp}}}^\perp$, so that $\widehat{a}_{{\phi '}}^\dagger$ commutes with the generators of the algebra to which $\widehat{H}^I$ belongs, hence with $\widehat{H}^I$. Moreover, we have using (94),

$$\begin{aligned} [{d\Gamma ({H^0})},{\widehat{a}_{{\phi '}}^\dagger }]=-i\frac{d}{dt}\left( t\mapsto \mathbb {H}({\widehat{a}_{{\phi '}}^\dagger })_{H^0}\right) (0)=\widehat{a}_{{H^0\phi '}}^\dagger , \end{aligned}$$

where $\mathbb {H}({\cdot })_{H^0}$ denotes the Heisenberg picture associated with the non-interacting Hamiltonian $d\Gamma ({H^0})$, so that we end up with

$$\begin{aligned} \frac{d}{dt}\langle \phi ,i\widetilde{G}(t)\phi ' \rangle =-i\Omega \left( \widehat{a}_{{\phi }} \mathbb {H}({\widehat{a}_{{H^0\phi '}}^\dagger })(-t)\right) =\langle \phi ,\widetilde{G}(t)H^0\phi ' \rangle . \end{aligned}$$

One shows, using the same techniques, that for all $t \in \mathbb {R}_-^*$,

$$\begin{aligned} \frac{d}{dt}\langle i\widetilde{G}(t)\phi ,\phi ' \rangle =\langle \widetilde{G}(t)H^0\phi ,\phi ' \rangle \end{aligned}$$

hence the first equality of (97) is proven. The second equality can be proved similarly.

#### Proof of Proposition A.14

We stick to the case in Remark A.12, where the AIM states are Gibbs states at inverse temperature $\beta$ and chemical potential $\epsilon _F$. Now for the first case, if $U_{ }=0$, the AIM are non-interacting, hence the Green’s functions are the non-interacting Green’s functions, the self-energies $\Sigma _{\textrm{imp},p}$ are identically zero, and the hybridization functions are given by, for all $z \in \mathbb {C}_+$,

$$\begin{aligned} \Delta _p(z)=W_p\left( z-H^0_{\overline{p}} \right) ^{-1} W_p^\dagger . \end{aligned}$$

Moreover, $\Sigma _{\textrm{DMFT}}=0$ so that $G_{\textrm{DMFT}}=G^0=G$ since the original Hubbard model is not interacting in this setting.

Now if the partition $\mathfrak {P}$ is such that $\mathcal {G}_{{H}}=\bigoplus _{p=1}^P\mathcal {G}_{{p}}$, or if $T_{ }=0$, we have

$$\begin{aligned} W_p=0, \end{aligned}$$

so that the hybridization functions $\Delta _p$ are identically zero. This is equivalent to zero-dimensional baths, and all the AIMs reduce to Hubbard models defined by $(\mathcal {G}_{{p}},T_{{p}},U_{{p}})$. Hence the self-energies $\Sigma _p$ are the self-energies associated with the Gibbs states of the corresponding Hubbard models.

Moreover, the clusters do not interact in this setting: the Hamiltonian $\widehat{H}_H$ of the original Hubbard model reads

98 $$\begin{aligned} \widehat{H}_H=\sum _{p=1}^P\widehat{H}_p, \end{aligned}$$

where $\widehat{H}_p$ is the Hamiltonians of the *p*-th Hubbard model defined on the induced subgraph $(\mathcal {G}_{{p}},T_{{p}},U_{{p}})$. By assumption, these Hamiltonians pairwise commute, so that the grand-canonical Gibbs state factorizes, and the associated Green’s functions $G$ and $G^0$ read

99 $$\begin{aligned} G=\bigoplus _{p=1}^P G_p, \quad G^0=\bigoplus _{p=1}^P G_p^0, \end{aligned}$$

where $G_p$ (resp. $G_p^0$) is the Green’s functions of the Gibbs state of the *p*-th Hubbard model (resp. *p*-th non-interacting Hubbard model). Therefore $\Sigma =\bigoplus _{p=1}^P\Sigma _p=\Sigma _{\textrm{DMFT}}$, which proves the claim.

#### Proof of Theorem A.17

We start by proving the equality. On the one hand, the Källén-Lehmann representation (56) of $\widetilde{G}$ associated with the grand canonical Hamiltonian $\widehat{H}'=\widehat{H}-\epsilon _F\widehat{N}$ reads for $\phi ,\phi ' \in \mathcal {H}_{ }$ and $z \in \mathbb {C}_+$,

$$\begin{aligned} \langle \phi ,G(z)\phi ' \rangle =\sum _{\psi ,\psi ' \in \mathcal {B}}(\rho _\psi +\rho _{\psi '})\langle \psi ,\widehat{a}_{{\phi }}\psi ' \rangle \langle \psi ',\widehat{a}_{{\phi '}}^\dagger \psi \rangle \frac{1}{z+\epsilon _F+(E_\psi -E_{\psi '})}. \end{aligned}$$

On the other hand, the Källén-Lehmann representation of $\widetilde{G}^M$ reads for $\phi ,\phi ' \in \mathcal {H}_{ }$ and $n \in \mathbb {N}$,

$$\begin{aligned} \langle \phi ,G^M_n\phi ' \rangle =\sum _{\psi ,\psi ' \in \mathcal {B}} \left( \rho _\psi + \rho _\psi e^{\beta (E_\psi -E_{\psi '}+\epsilon _F)} \right) \langle \psi ,\widehat{a}_{{\phi }}\psi ' \rangle \langle \psi ',\widehat{a}_{{\phi '}}^\dagger \psi \rangle \frac{1}{i\omega _n + \epsilon _F+(E_\psi -E_{\psi '})}. \end{aligned}$$

Note now that whenever $\langle \psi ,\widehat{a}_{{\phi }}\psi ' \rangle \ne 0$, we have $N_{\psi '}=N_{\psi }+1$ and then

$$\begin{aligned} \rho _\psi e^{\beta (E_\psi -E_{\psi '}+\epsilon _F)} = e^{-\beta (E_{\psi '}-\epsilon _F(N_\psi +1))}=\rho _{\psi '}, \end{aligned}$$

yielding

$$\begin{aligned} \forall n \in \mathbb {N},\ G(i\omega _n)=G^M_{n}. \end{aligned}$$

To prove uniqueness, we use the fact that $-G$ is a Pick matrix (see Proposition A.1), and its Nevanlinna-Riesz measure is a weighted sum of finitely many Dirac measures. It follows that for all $\phi \in \mathcal {H}_{ }$, the Analytic Continuation Problem defined by $\left( i\omega _n,-\langle \phi ,G^M_n\phi \rangle \right) _{n \in \mathbb {N}}$ has no other solution thanks to Theorem B.9, which concludes.

## Uniqueness Theorem for An Interpolation Problem

The Nevanlinna-Pick Analytic Continuation Problem (ACP), which we will also call *interpolation problem*, has been studied in the beginning of the 20th century independently by Nevanlinna ([56], 1919) and Pick ([60], 1915). The results presented in Sect. B.1 are gathered in the book [73]. Many other references address this problem, such as [1, 24, 66]. In Sect. B.1, we set up the problem and then give some results that are important to our analysis. Other important results on the ACP and characterizations of the solutions are detailed in the references. For example, we will not discuss the question of extremal solutions ( [58, 69]), nor the use of Blaschke products ( [24, 57, 73]). The approaches of Nevanlinna and Pick are different, we chose here to focus on Nevanlinna’s approach.

### Introduction and Some General Properties

The Nevanlinna-Pick Analytic Continuation Problem (ACP) can be stated as follows. Let $(z_n)_{n\in I}$ be a sequence of distinct points in the open upper half-plane $\mathbb {C}_+$ and let $(w_n)_{n\in I}$ be a sequence in $\overline{\mathbb {C}_+}$. We want to answer the following question.

#### Question B.1

Is there an analytic function $f:\mathbb {C}_+\rightarrow \overline{\mathbb {C}_+}$ interpolating the given values at the prescribed points? In other words, we look for a Pick function *f* such that

100 $$\begin{aligned} \forall n \in I,\ f(z_n)=w_n, \end{aligned}$$

where *I* is a (at most) countable set.

Without loss of generality, we can assume that $I=\{1,2,\ldots \}$. We denote by $AC_{{\mathbb {C}_+}}(z_n,w_n)_{I}$ the set of solutions to this problem (we will omit the dependence on *I*, unless when needed). Both sets $\mathbb {C}_+$ and $\overline{\mathbb {C}_+}$ are invariant under the action of the subset $\mathcal {T}$ of affine transformations of $\overline{\mathbb {C}_+}$ given by $\mathcal {T} = \{ \tau : \overline{\mathbb {C}_+} \rightarrow \overline{\mathbb {C}_+}, z \mapsto az+b,\ b \in \mathbb {R},\ a > 0 \}$, and we have

101 $$\begin{aligned} \forall \tau _1,\tau _2 \in \mathcal {T},\quad AC_{{\mathbb {C}_+}}(\tau _1(z_n),\tau _2(w_n))= \tau _2 \circ AC_{{\mathbb {C}_+}}(z_n,w_n) \circ \tau _1^{-1}. \end{aligned}$$

Moreover, question B.1 can equivalently be stated in the unit disc instead of the upper half-plane:

#### Question B.2

Let $(z_n)$ and $(w_n)$ be sequences in the unit disc $\mathbb {D}=\{z\in \mathbb {C},|z|<1\}$ and the closed disc $\overline{\mathbb {D}}$ respectively. Is there an analytic function $f:\mathbb {D}\rightarrow \overline{\mathbb {D}}$ such that $\forall n \in I, \quad f(z_n)=w_n$ ?

The reason for the equivalence between both formulations is that the upper half-plane can be mapped to the unit disc through the Cayley transform, which is biholomorphic between these sets. The Cayley transform $\mathcal {W}$ and its reciprocal $\mathcal {W}^{-1}$ are given by:

$$\begin{aligned} \begin{array}{rclcrcl} \mathcal {W}: \mathbb {C}\backslash \{-i\} &  \longrightarrow &  \mathbb {C}\backslash \{1\} &  \quad &  \mathcal {W}^{-1}: \mathbb {C}\backslash \{1\} &  \longrightarrow &  \mathbb {C}\backslash \{-i\} \\ z &  \mapsto &  \dfrac{z-i}{z+i} &  \quad &  z &  \mapsto &  i\dfrac{1+z}{1-z}. \end{array} \end{aligned}$$

As the Cayley transform $\mathcal {W}$ is biholomorphic from $\mathbb {C}_+$ to $\mathbb {D}$ and maps the real line $\mathbb {R}$ to the unit circle deprived of the point 1, we have the equivalence of the following statements, with $F: \mathbb {C}_+\rightarrow \overline{\mathbb {C}_+}$, $(z_n)\subset \mathbb {C}_+$, $(w_n)\subset \overline{\mathbb {C}_+}$, $f=\mathcal {W}\circ F \circ \mathcal {W}^{-1}: \mathbb {D}\rightarrow \overline{\mathbb {D}}$, $\tilde{z_n}=\mathcal {W}(z_n)\in \mathbb {D}$ and $\tilde{w_n }=\mathcal {W}(w_n)\in \overline{\mathbb {D}}$.

$$\begin{aligned} \forall n\ge 1, \ F(z_n) = w_n \iff \forall n\ge 1,\ f(\tilde{z_n}) = \tilde{w_n}. \end{aligned}$$

In other words, denoting by $AC_{{\mathbb {D}}}(z_n,w_n)$ the set of solutions to Question B.2, we have

102 $$\begin{aligned} AC_{{\mathbb {D}}}(\mathcal {W}(z_n),\mathcal {W}(w_n))=\mathcal {W}\circ AC_{{\mathbb {C}_+}}(z_n,w_n)\circ \mathcal {W}^{-1}. \end{aligned}$$

As a matter of fact, we can answer Question B.2 when the sequence $(w_n)$ is constant and of modulus 1, as a direct consequence of the maximum modulus principle.

#### Lemma B.3

Given $$C \in \overline{\mathbb {D}} \setminus \mathbb {D}$$, $$AC_{{\mathbb {D}}}(z_n,C)= \{ C \}$$.

Combined with equation (102), we have a first statement about the set of solutions $AC_{{\mathbb {C}_+}}$ to the interpolation problem (100).

#### Corollary B.4

Given $$C \in \mathbb {R}$$, $$AC_{{\mathbb {C}_+}}(z_n,C)=\{ C \}$$.

The next definition introduces the functions $b_a$ (the notation suggests Blaschke products, see [58] and [24]), which will be useful for our analysis of $AC_{{\mathbb {D}}}(z_n,w_n)$.

#### Definition B.5

Let $a \in \mathbb {D}$. Define for $z\in \mathbb {D}$, $b_{a}(z)=\frac{z-a}{1-\overline{a}z} \in \mathbb {D}$.

These functions are in fact biholomorphic from $\mathbb {D}$ to itself and $b_a$ only vanishes in $a\in \mathbb {D}$. This zero is of order 1. The function $b_a$ can actually be extended to the closed disc $\overline{\mathbb {D}}$, and we will also denote this extension $b_a$. Considering our interpolation problem, we notice the following. If *f* is a solution to the interpolation problem stated in question B.2, we have $f(z_1)=w_1$, with $|w_1|\le 1$. If the modulus of $w_1$ is 1, then *f* is constant because of the maximum modulus principle. Suppose this is not the case and define the function $f^{(1)}$ by

103 $$\begin{aligned} f^{(1)}(z) = \frac{b_{w_1}(f(z))}{b_{z_1}(z)}. \end{aligned}$$

$f^{(1)}$ is then well-defined on $\mathbb {D}$, because the only zero of $b_{z_1}$ is $z_1$ and is of order 1, and $z_1$ is also a zero of order at least 1 of $b_{w_1}\circ f$. One can check that $f^{(1)}$ takes values in $\overline{\mathbb {D}}$, so that $f^{(1)}$ is analytic from $\mathbb {D}$ to $\overline{\mathbb {D}}$ if and only if $f=b_{w_1}^{-1}\circ (b_{z_1}f^{(1)})$ is analytic from $\mathbb {D}$ to $\overline{\mathbb {D}}$.

Now suppose $\vert {I}\vert \ge 2$ and define for all $n \in I \setminus \{ 1 \}$, $w_n^{(1)}:= \frac{b_{w_1}(w_n)}{b_{z_1}(z_n)}$. We notice that $f^{(1)}(z_n) = \frac{b_{w_1}(f(z_n))}{b_{z_1}(z_n)} = \frac{b_{w_1}(w_n)}{b_{z_1}(z_n)} = w_n^{(1)}$. This means that $f^{(1)}$ is the solution to the Nevanlinna-Pick interpolation problem

104 $$\begin{aligned} g(z_n) = w_n^{(1)}, \ \forall n\in I \setminus \{ 1 \}. \end{aligned}$$

We have proven the following lemma, which is the main element of the Schur interpolation algorithm [32].

#### Lemma B.6

(Schur iteration). Assume $\vert {I}\vert \ge 2$ and $w_1 \in \mathbb {D}$. We then have the following equivalence:

$$\begin{aligned} f \in AC_{{\mathbb {D}}}(z_n,w_n)_I \iff f^{(1)}: z \mapsto \frac{b_{w_1}(f(z))}{b_{z_1}(z)} \in AC_{{\mathbb {D}}}(z_n,w_n^{(1)})_{I\setminus \{1 \}}. \end{aligned}$$

In other words,

$$\begin{aligned} AC_{{\mathbb {D}}}(z_n,w_n)_I=b_{w_1}^{-1}\circ \left( b_{z_1} \cdot AC_{{\mathbb {D}}}(z_n,w_n^{(1)})_{I\setminus \{1 \}} \right) . \end{aligned}$$

Both previous lemmas give the intuition about the following theorem, which can be found in any reference dealing with the issue of Nevanlinna-Pick interpolation, e.g. [24, 57, 73]. Refinements of this result and characterizations of the solutions are detailed in the references given at the beginning of this section.

#### Theorem B.8

Let $(z_n)_{n\ge 1}$ and $(w_n)_{n\ge 1}$ be sequences in the unit disc $\mathbb {D}$ and the closed disc $\overline{\mathbb {D}}$ respectively. Define by induction, for $1\le l$ and $k>l$,

105 $$\begin{aligned} w_k^{(l)}:= \dfrac{w_k^{(l-1)}-w_l^{(l-1)}}{1-\overline{w_l^{(l-1)}}w_k^{(l-1)}}\dfrac{1-\overline{z_l}z_k}{z_k-z_l} = \dfrac{b_{w_l^{(l-1)}}(w_k^{(l-1)})}{b_{z_l}(z_k)}, \end{aligned}$$

with $$w_k^{(0)}=w_k$$ for $$k\ge 1$$.

There are three distinct cases.

1. If there exists $k\ge 1$ such that $|w_k^{(k-1)}|>1$, then there is no solution to the interpolation problem (100).
2. Else, if there exists $K\ge 1$ such that for all $1\le k < K$, $|w_k^{(k-1)}|<1$, $|w_K^{(K-1)}|=1$ and for all $l\ge K$, $w_l^{(K-1)} = w_K^{(K-1)}$, then there exists a unique solution to the interpolation problem (100).
3. Else, we have for all $k\ge 1$, $|w_k^{(k-1)}|<1$ and there is either 1 or infinitely many solutions to the interpolation problem.

### A Uniqueness Result for ACPs with a Rational Solution

In our mathematical framework, we need the following uniqueness theorem (Theorem B.9). In this section, we are tackling the uniqueness of the interpolation problem (100), in the case where we have already found one solution of some specific form. We assume that we have found a solution *f* such that its Nevanlinna-Riesz measure in the integral representation (10) is a finite sum of weighted Dirac measures, that is *f* is a rational function. This means that we have $f(z)= \alpha z +C+ \sum _{k=1}^K\frac{a_k}{z-\varepsilon _k}$, with $\alpha \ge 0$, $C\in \mathbb {R}$, $K\in \mathbb {N}$, the $a_k$’s are negative numbers and the $\varepsilon _k$’s are distinct real numbers. The following theorem states that it is then the only solution to the interpolation problem (100). The theorem is actually a corollary of a result by Donoghue [17], stated within the Pick formalism of Pick functions. The approach and the proof presented here are however different, and may therefore be of interest to the reader.

#### Theorem B.9

Let $f: \mathbb {C}_+\rightarrow \overline{\mathbb {C}_+}$ be a Pick function such that its Nevanlinna-Riesz measure $\mu$ is a finite sum of Dirac measures: there exist $K \in \mathbb {N}$, $a_1,\dots ,a_K < 0$ and distinct real numbers $\varepsilon _1,\dots ,\varepsilon _K$ such that

$$\begin{aligned} \mu =\sum _{k=1}^K a_k \delta _{\varepsilon _k}. \end{aligned}$$

Then, if *f* is a solution to an interpolation problem $AC_{{\mathbb {C}_+}}(z_n,w_n)_I$ with $\vert {I}\vert \ge K+2$, this problem has no other solution.

#### Proof

We prove this result by strong induction on *K*, for $K \in \mathbb {N}$. If $K=0$, the expression of *f* reads $f(z)=\alpha z+C$, with $\alpha \ge 0$ and $C\in \mathbb {R}$. Now suppose $f \in AC_{{\mathbb {C}_+}}(z_n,w_n)$. Then for all $n \in I$, we have $\alpha z_n +C= w_n$, so that

$$\begin{aligned} AC_{{\mathbb {C}_+}}(z_n,w_n)=AC_{{\mathbb {C}_+}}(z_n,\alpha z_n+C). \end{aligned}$$

If $\alpha =0$, then $AC_{{\mathbb {C}_+}}(z_n,C)=\{ C \}$ by corollary B.4. Else, we extend continuously *f* to $\overline{\mathbb {C}_+}$ into the affine transformation $\widetilde{f} \in \mathcal {T}$ and we have

$$\begin{aligned} AC_{{\mathbb {C}_+}}(z_n,w_n)= \widetilde{f} \circ \mathcal {W}^{-1} \circ AC_{{\mathbb {D}}}(\mathcal {W}(z_n),\mathcal {W}(z_n)) \circ \mathcal {W}. \end{aligned}$$

Denoting by $\widetilde{z}_n=\widetilde{w}_n=\mathcal {W}(z_n)$, we find that for all $n \in I \setminus \{1\}, \widetilde{w}^{(1)}_n=1$. Hence by Theorem B.8, $AC_{{\mathbb {D}}}(\mathcal {W}(z_n),\mathcal {W}(z_n))= \{ \textrm{id}_\mathbb {D}\}$ and $AC_{{\mathbb {C}_+}}(z_n,w_n)=\{ f \}$.

Now assume the result holds for $l \le K$ and take *f* such that its Nevanlinna-Riesz measure is a sum of $K+1$ Dirac measures. Now consider an ACP $AC_{{\mathbb {C}}}(z_n,w_n)_I$ with $\vert {I}\vert \ge K+3$ to which *f* is a solution, namely $f(z_n)=w_n$ and there exist $\alpha \ge 0$, $C\in \mathbb {R}$, $a_1,\dots ,a_{K+1} < 0$ and real numbers $\varepsilon _1<\dots <\varepsilon _{K+1}$, such that for all $z \in \mathbb {C}_+$,

$$\begin{aligned} f(z)= \alpha z + C + \sum _{k=1}^{K+1}\frac{a_k}{z-\varepsilon _k}. \end{aligned}$$

We start by making use of the property  (101) to simplify the problem. It is possible to chose two affine transformations $\tau _{z_1}$ and $\check{\tau }_{z_1}$ in $\mathcal {T}$ such that, setting $\widetilde{f}=\check{\tau }_{z_1} \circ f\circ \tau _{z_1}^{-1}$, we have

$$\begin{aligned} \widetilde{f}(z)= \widetilde{C} + \widetilde{\alpha } z + \sum _{k=1}^{K+1}\frac{\widetilde{a}_k}{z-\widetilde{\varepsilon }_k}, \end{aligned}$$

where the parameters $\widetilde{C}$, $\widetilde{\alpha }$, $\widetilde{a}_k$, and $\widetilde{\varepsilon }_k$ satisfy the same properties as their counterparts without the tildes, and such that

106 $$\begin{aligned} \tau _{z_1}(z_1)=i, \quad \Re (\widetilde{f}(\widetilde{z}_1))=0 \quad \text { and }\quad \sum _{k=1}^{K+1}\frac{-\widetilde{a}_k}{1+\widetilde{\varepsilon }_k^2}=1. \end{aligned}$$

Using (101), we have

$$\begin{aligned} AC_{{\mathbb {C}_+}}(z_n,w_n)= \check{\tau }_{z_1}^{-1}\circ AC_{{\mathbb {C}_+}}(\underbrace{\tau _{z_1}(z_n)}_{\widetilde{z}_n},\underbrace{(\check{\tau }_{z_1} \circ f\circ \tau _{z_1}^{-1})}_{\widetilde{f}}(\tau _{z_1}(z_n)))\circ \tau _{z_1}. \end{aligned}$$

For the remaining of the proof, we will then focus on $AC_{{\mathbb {C}_+}}(\widetilde{z}_n,\widetilde{f}(\widetilde{z}_n))$. We will omit the tildes for the sake of simplicity, and assume that $z_1=i$ and that (106) holds. With that being said, notice that $\Im (f(z_1))=\Im (f(i))=\alpha + \sum _{k=1}^{K+1} \frac{-a_k}{1+\varepsilon _k^2}=\alpha +1 > 0$, hence $\mathcal {W}(f(z_1)) \in \mathbb {D}$. Therefore, using Lemma B.6, we have

$$\begin{aligned} AC_{{\mathbb {D}}}(\mathcal {W}(z_n),\mathcal {W}(f(z_n)))_I=b_{\mathcal {W}(f(z_1))}^{-1}\circ \left( b_{z_1} \cdot AC_{{\mathbb {D}}}(\mathcal {W}(z_n),\mathcal {W}(f(z_n))^{(1)})_{I\setminus \{1 \}} \right) , \end{aligned}$$

and, using equation (102),

$$\begin{aligned} AC_{{\mathbb {D}}}(\mathcal {W}(z_n),\mathcal {W}(f(z_n))^{(1)})_{I\setminus \{1 \}}= \mathcal {W}\circ AC_{{\mathbb {C}_+}}\left( z_n,\mathcal {W}^{-1}([\mathcal {W}(f(z_n))]^{(1)})\right) _{I\setminus \{1 \}} \circ \mathcal {W}^{-1}. \end{aligned}$$

We now compute $g(z) = \mathcal {W}^{-1}\circ [\mathcal {W}\circ f]^{(1)}(z)$, for $z \in \mathbb {C}_+\setminus \{i\}$. Since $\mathcal {W}(z_1) = \mathcal {W}(i)=0$ and $z\ne i$, we have $b_{\mathcal {W}(z_1)}(\mathcal {W}(z))=\mathcal {W}(z)\ne 0$ and *g*(*z*) is well defined. The computation reads

$$\begin{aligned} g(z) = i\frac{(f(z_1)+i)\frac{\overline{f(z_1)}-f(z)}{z+i}+\overline{(f(z_1)+i)}\frac{f(z)-f(z_1)}{z-i}}{(f(z_1)+i)\frac{\overline{f(z_1)}-f(z)}{z+i}-\overline{(f(z_1)+i)}\frac{f(z)-f(z_1)}{z-i}}. \end{aligned}$$

We used the fact that since $\overline{\mathcal {W}(f(z_1))}\mathcal {W}(f(z)) \in \mathbb {D}$, it is not equal to 1 and that $\frac{1}{2i}(f(z)+i)\vert (f(z_1)+i)\vert ^2 \ne 0$. Now, realize that, since $z_1=i$, we have

$$\begin{aligned} \frac{f(z)-f(z_1)}{z-i}= \alpha + \sum _{k=1}^{K+1}\frac{1}{\varepsilon _k-i}\frac{a_k}{z-\varepsilon _k}, \end{aligned}$$

and similarly

$$\begin{aligned} \frac{\overline{f(z_1)}-f(z)}{z+i} = -\alpha - \sum _{k=1}^{K+1}\frac{1}{\varepsilon _k+i}\frac{a_k}{z-\varepsilon _k}, \end{aligned}$$

so that

107 $$\begin{aligned} g(z)=-\frac{\alpha (\Im (f(z_1))+1) + \sum _{k=1}^{K+1}\Im \left( \frac{f(z_1)+i}{\varepsilon _k+i}\right) \frac{a_k}{z-\varepsilon _k}}{\alpha \Re (f(z_1)) + \sum _{k=1}^{K+1}\Re \left( \frac{f(z_1)+i}{\varepsilon _k+i}\right) \frac{a_k}{z-\varepsilon _k}}, \end{aligned}$$

which holds for $z=i$ as well. As we set $\Re (f(z_1))=0$, we have

$$\begin{aligned} \Re \left( \frac{f(z_1)+i}{\varepsilon _k+i}\right) =\frac{\Im (f(z_1))+1}{1+\varepsilon _k^2} > 0 \quad \text { and } \quad \Im \left( \frac{f(z_1)+i}{\varepsilon _k+i}\right) =\varepsilon _k\frac{\Im (f(z_1))+1}{1+\varepsilon _k^2}. \end{aligned}$$

Multiplying the numerator and denominator in (107) by $\frac{1}{\Im (f(z_1))+1}\prod _{k=1}^{K+1}(z-\varepsilon _k)$, we end up with $\displaystyle g(z)=\frac{P(z)}{Q(z)}$, with

$$\begin{aligned} P(z):= \alpha \prod _{k=1}^{K+1}(z-\varepsilon _k) + \sum _{k=1}^{K+1}\varepsilon _k \frac{a_k}{1+\varepsilon _k^2} \prod _{l=1,l\ne k}^{K+1}(z-\varepsilon _l), \end{aligned}$$

and

$$\begin{aligned} Q(z):= \sum _{k=1}^{K+1}\frac{-a_k}{1+\varepsilon _k^2} \prod _{l=1,l \ne k}^{K+1} (z-\varepsilon _l). \end{aligned}$$

We have $Q(\varepsilon _k)=\frac{-a_k}{1+\varepsilon _k^2}\prod _{l=1,l \ne k}^{K+1}(\varepsilon _k-\varepsilon _l) \ne 0$ by hypothesis on the $\varepsilon _k$’s, so that

$$\begin{aligned} Q(z)=0 \iff \sum _{k=1}^{K+1}\frac{\frac{-a_k}{1+\varepsilon _k^2}}{z-\varepsilon _k}=0, \end{aligned}$$

hence *Q* admits exactly *K* distinct roots $\varepsilon '_k \in (\varepsilon _k,\varepsilon _{k+1}) \subset \mathbb {R}$ by the intermediate value theorem (as in the proof of Proposition 3.3 in Sect. 4.2), and it is unitary due to (106). The partial fraction decomposition of *g* finally reads

$$\begin{aligned} g(z)=\alpha ' z + C'+ \sum _{k=1}^K\frac{a'_k}{z-\varepsilon '_k}, \end{aligned}$$

where

$$\begin{aligned} \begin{array}{rcl} \alpha '& =& \alpha , \\ C'& =& \displaystyle \underset{ y \rightarrow \infty }{\lim }\ g(iy)-i\alpha 'y=(1-\alpha )\sum _{k=1}^{L+1}\varepsilon _k\frac{a_k}{1+\varepsilon _k^2} \in \mathbb {R}, \\ a'_k& =&  \underset{y \rightarrow 0^+}{\lim }\ iyg(\varepsilon '_k+iy) \\ &  =&  \left( \prod _{l=1,l\ne k}^K \underbrace{\left( \frac{\varepsilon '_k-\varepsilon _l}{\varepsilon '_k-\varepsilon '_l}\right) }_{>0} \right) \underbrace{(\varepsilon '_k-\varepsilon _k)(\varepsilon '_k-\varepsilon _{L+1})}_{<0}\underbrace{(\alpha + 1)}_{> 0} < 0. \end{array} \end{aligned}$$

This computation shows that the Nevanlinna-Riesz measure of *g* is a sum of *K* Dirac measures as described in the statement of the theorem. It is a solution to the Analytic Continuation Problem

$$\begin{aligned}AC_{{\mathbb {C}_+}}\left( z_n,\mathcal {W}^{-1}([\mathcal {W}(f(z_n))]^{(1)}])\right) _{I\setminus \{1 \}},\end{aligned}$$

where $\vert {I\setminus \{1\}}\vert \ge K+2$ by assumption. By the induction hypothesis, *g* is the only solution to this ACP, and therefore *f* is the only solution to the ACP $AC_{{\mathbb {C}_+}}(w_n,z_n)_I$. $\square$
